# A review of the botany, metabolites, pharmacology, toxicity, industrial applications, and processing of Polygalae Radix: the “key medicine for nourishing life”

**DOI:** 10.3389/fphar.2024.1450733

**Published:** 2024-09-18

**Authors:** Hongtuo Kuang, Lingping Kong, Ajiao Hou, Anni Yang, Hai Jiang

**Affiliations:** Key Laboratory of Basic and Application Research of Beiyao, Heilongjiang University of Chinese Medicine, Ministry of Education, Harbin, China

**Keywords:** Polygalae Radix, pharmacological effects, metabolites, applications, toxicity, processing

## Abstract

*Polygalae radix* (PR) is the dried root of *Polygala tenuifolia* Willd. and *Polygala sibirica* L. and enjoys the reputation as the “key medicine for nourishing life.” In this study, information about “*Polygala tenuifolia* Willd.,” “*Polygala sibirica* L.,” and “Yuanzhi” was retrieved from scientific databases, including Google Scholar, Baidu Scholar, Web of Science, PubMed, CNKI, and Wan Fang Data. Information from Chinese herbal medicine classics, Yaozhi Data, and the Gaide Chemical Network was also collected. Information related to botany, phytochemistry, pharmacology, toxicity, industrial applications, and processing is summarized in this paper to tap its potentialities and promote its further development and clinical application. More than 320 metabolites have been isolated from PR; saponins, xanthones, and oligosaccharide esters are the main functional metabolites. Pharmacological research shows that its pharmacological action mainly focuses on resisting nervous system diseases, and it also has the functions of anti-oxidation, anti-inflammation, anti-tumor, anti-pathogenic microorganisms and others. The gastrointestinal irritation of its saponins impeded its application, but this irritation can be reduced by controlling the dosage, compatibility with other herbs, or processing. The future progress of PR faces opportunities and challenges. More attention should be paid to the traditional application and processing methods of PR recorded in ancient books. The lack of safety and clinical studies has limited its application and transformation of achievements. Moreover, it is one-sided to take the content of only a few metabolites as the index of processing optimization and quality control, which cannot reflect the full pharmacological and toxicological activities of PR.

## Graphical Abstract

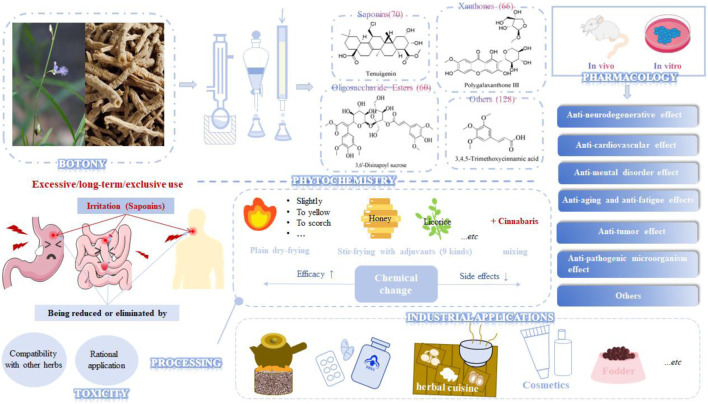

## Introduction

Polygalae Radix (PR), the dried root of *Polygala tenuifolia* Willd. or *Polygala sibirica* L., is mainly distributed in China, South Korea, Mongolia, and Russia (Jiang et al., 2016). PR is widely used in Japanese Kampo medicine (Tsukada et al., 2021), traditional Korean medicine (Kumar et al., 2013), traditional Chinese medicine (TCM), and Mongolian medicine as a tonic, expectorant, sedative, and antasthmatic. It is known as “Onji” in Japan (Kuroda et al., 2014) and “Wonji” in South Korea, and these terms refer only to the root or root bark of *Polygala tenuifolia* Willd. In China, PR is called “Yuanzhi” and ranks as the third most frequently used single herb in the clinical application of cognitive enhancement prescriptions (Peng et al., 2020a). According to the 2020 Chinese Pharmacopoeia, PR is usually harvested in spring and autumn, the fibrous root and silt are removed, and the product is dried directly or after the wood heart is removed. It possesses bitterness and pungency in flavor and warmth in nature, enters the heart and lung meridian, and holds the effects of calming the nerves and increasing intelligence, coordinating the heart and kidney, eliminating phlegm, and relieving swelling. It is usually used for treating insomnia and dreamful sleep, amnesia, palpitation, and obnubilation caused by heart-kidney imbalance, as well as ungratifying coughing of phlegm, swelling and sores, and breast pain. Other traditional applications of PR are shown in Supplementary Material.

According to Yaozhi Data, more than 800 prescriptions contain PR in diverse dosage forms, especially decoctions, pills, and powders. In 2018, three classic prescriptions containing PR, Kai-Xin-San, Di-Huang-Yin-Zi, and Gu-Yin-Jian, were included in the *Catalogue of Ancient Classic Prescriptions* (the First Batch) by the state administration of TCM. PR is praised as “the key medicine for nourishing life” by Shennong’s *Herbal Classic*. As a homologous plant of medicine and food, it shows great health-preserving potential in the fields of healthcare products and medicated diets. Owing to the continuous discovery of pharmacological activities, its application scope in the daily chemical industry tends to deepen and expand, giving it increasing commercial value.

More than 320 metabolites have been isolated from PR, including its main active ingredients—saponins, xanthones, and oligosaccharide esters, as well as other metabolites such as alkaloids, coumarins, lignins, and flavonoids (Wang et al., 2020a). Abundant research on the pharmacological activities of PR mainly focuses on its anti-neurological disease effects (Zhao et al., 2020) such as neurodegenerative diseases (NDD, such as Alzheimer’s disease (AD), Parkinson’s disease (PD), Huntington’s disease (HD), aging, learning, and memory impairment, spinal cord injury), cardio-cerebrovascular diseases (such as hypoxia-reoxygenation injury, cerebral ischemia-reperfusion, myocardial ischemia, cerebral hemorrhage, arrhythmia) and mental disorders (such as depression, insomnia, anxiety, post-traumatic stress disorder, social disorder). In addition, it has antioxidant, anti-inflammatory, anti-fatigue, anti-tumor, anti-pathogenic microorganisms and other functions (Zheng et al., 2016; Lei et al., 2020; Yu, 2020; Huang and Lin, 2021; Wang et al., 2021a; Zeng et al., 2021a; Gao, 2022; Yang et al., 2022). According to literature reports, it also has certain biological activities in eliminating phlegm, relieving cough, killing sperm, lowering blood pressure, resisting diabetes, contracting the uterus, protecting the stomach, resisting bone loss, obesity, resisting fatty liver, promoting blood coagulation, and resisting radiation.

A comprehensive review is necessary to deepen the understanding of PR, facilitate further study, and guide its rational application. In this study, we reviewed PR from the aspects of botany, phytochemistry, pharmacology, toxicology, industrial applications, and processing (*Graphic Abstract*). In addition, the limitations to current research and the possible directions of future research were also discussed. This review is of great significance for propelling the deep ongoing research and expanding the application scope of PR.

## Botany

### Morphological feature

**FIGURE 1:**
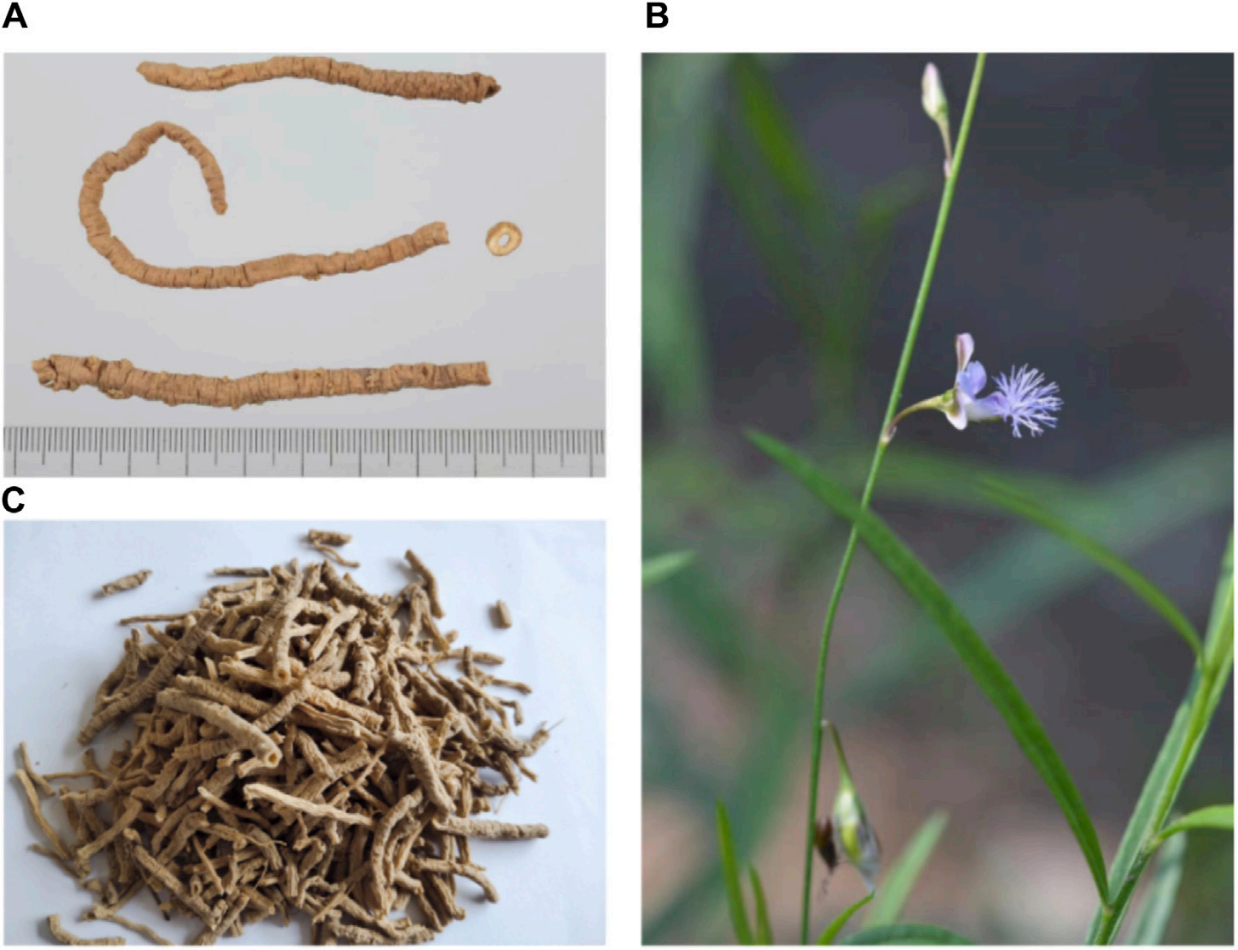
Root **(A)**, aerial part **(B)**, and medicinal pieces **(C)** of *Polygalae radix*.

PR (Figure 1) is the dried root of two perennials, *Polygala tenuifolia* Willd. and *Polygala sibirica* L. According to the Higher Plants of China (中国高等植物) and Flora of China (中国植物志), their botanical characteristics are shown in Supplementary Table 1. Saponins, PR’s main effective metabolite, mostly accumulate in its roots, stems, and leaves, with root bark having dozens of times higher content than xylem and aerial parts (Teng et al., 2009a). The root or root bark of PR is often used medicinally, and aerial parts are used in a few areas and are mainly discarded. The saponin content begins to increase at the flowering stage and peaks at the fruiting stage. The plant should be harvested in April and May, and the total saponin yield of a three-year-old PR is the highest (Teng et al., 2009b).

According to the *Commercial Grades for Traditional Chinese Medicine* distributed by the Chinese Traditional Medicine Association, the medicinal pieces of PR can be generally divided into three specifications: “Yuanzhi tong,” “Yuanzhi rou,” and “Yuanzhi gun.” “Yuanzhi tong” refers to the root bark of the thicker PR with a hollow cylinder shape, which has been loosened by kneading or other means, and its wood heart has been removed. According to the different removal rates of wood core and diameters, the “Yuanzhi tong” is subdivided into five grades, in which the diameter and removal rate of the wood core of the super ones exceed 0.4 cm and 95%, respectively, and the removal rate of the wood core of the lowest grade is ≥ 80%. “Yuanzhirou” is the ruptured root bark of narrower PR that has been smashed with a wood stick and had its wood heart removed. PR, which is too slender to remove the wood heart, is called “Yuanzhigun.” Among them, “Yuanzhitong” is considered to be the main type with superior quality; it is more expensive and is the opposite of “Yuanzhigun.”

Note that counterfeit products are rampant in the market; they are cheaper and similar in appearance to PR but are very different in flavor and efficacy. These adulterants not only degrade the quality of PR but also compromise the efficacy and safety of related compositions and formulations (Li et al., 2019). To distinguish PR from its adulterated products, we summarized common adulterants and their characteristics in Supplementary Table 2.

### Plant resources

**FIGURE 2:**
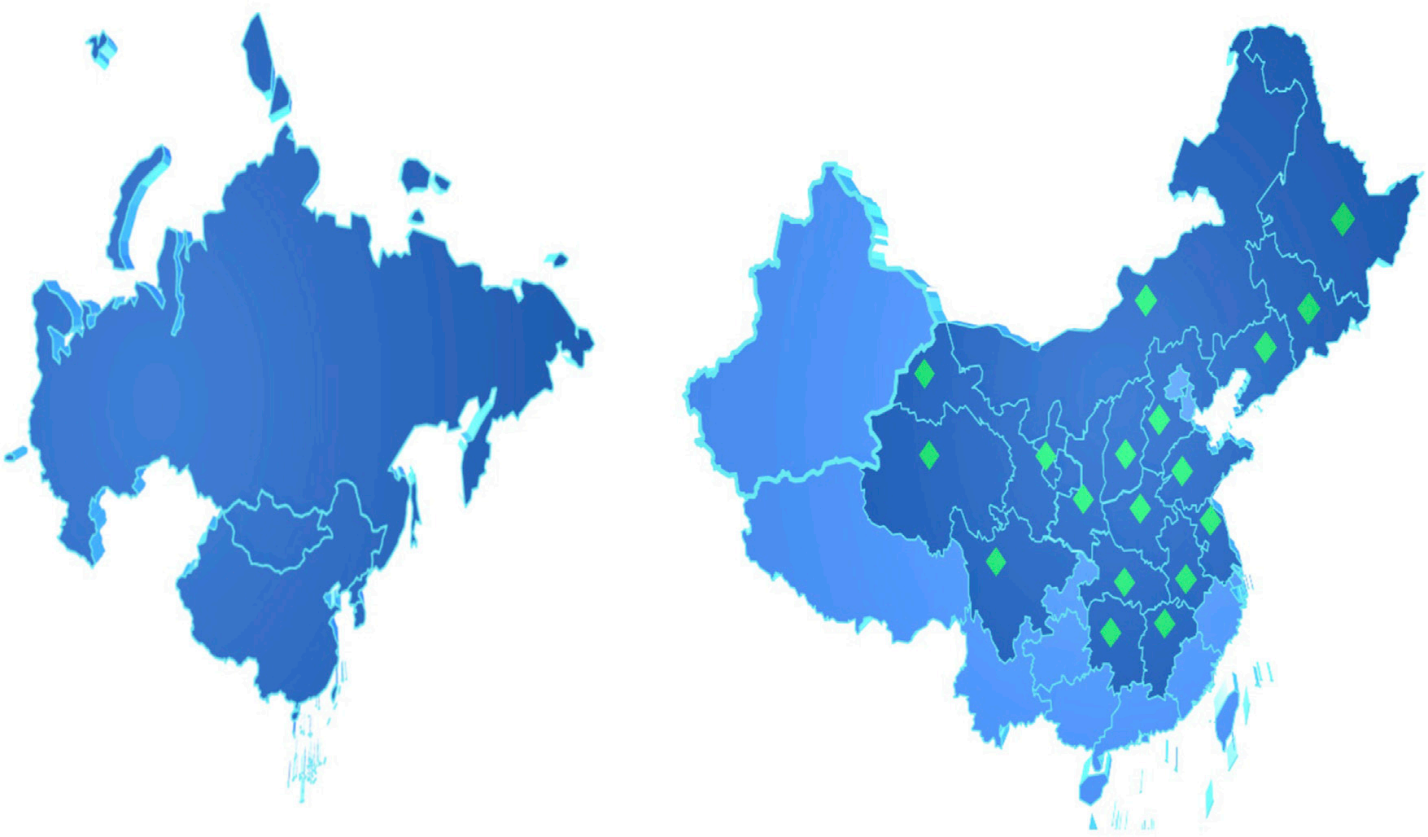
Geographical distribution of *Polygalae radix* in the world (left) and in China (right).

PR is mainly distributed in the temperate regions of East Asia and Europe (Figure 2), such as China, South Korea, Mongolia, the Korean Peninsula, and the Russian Federation. In China, Heilongjiang, Jilin, Liaoning, Inner Mongolia, Shanxi, Hebei, Shandong, Henan, Anhui, Jiangsu, Jiangxi, Hubei, Hunan, Sichuan, Qinghai, Gansu, Ningxia, and Shaanxi are all its producing areas. Shanxi and Shaanxi have the largest yield (Fan et al., 2020). On 2 April 2022, PR was included in the Catalogue of Genuine Medicinal Materials in Heilongjiang Province. Although it is one of the bulk medicinal materials and 85 traditional export herbs, the slow growth rate and finite production of wild PR cannot meet the huge market demands. As a result of over-exploitation, vegetation destruction, and global warming, wild PR resources are increasingly scarce (Liu et al., 2021). The relevant departments of the Chinese government have brought PR into the national third-class protected wild plants list and issued a policy to prohibit its harvest and trafficking. Apart from setting up nature reserves, improving the *in-situ* protection system, taking *ex situ* protection, and *in vitro* preservation when necessary, it is also a feasible measure to study the suitable growth conditions of PR and replace wild products with cultivated products (Peng et al., 2018; Deng and Jin, 2020). PR is drought-tolerant and likes cool temperatures, which are common on hillsides facing the sun, forest edges, roadsides, and ridges. PR is favorable to be grown where the warmest seasonal and annual precipitation range from 148 mm to 512 mm and 300 mm to 500 mm, respectively, the annual average temperature is between 8.4°C and 15.4°C, the total solar radiation is 502.42∼586.15 J/cm, and the altitude is between 100 m and 2,000 m.

## Metabolites

**FIGURE 3:**
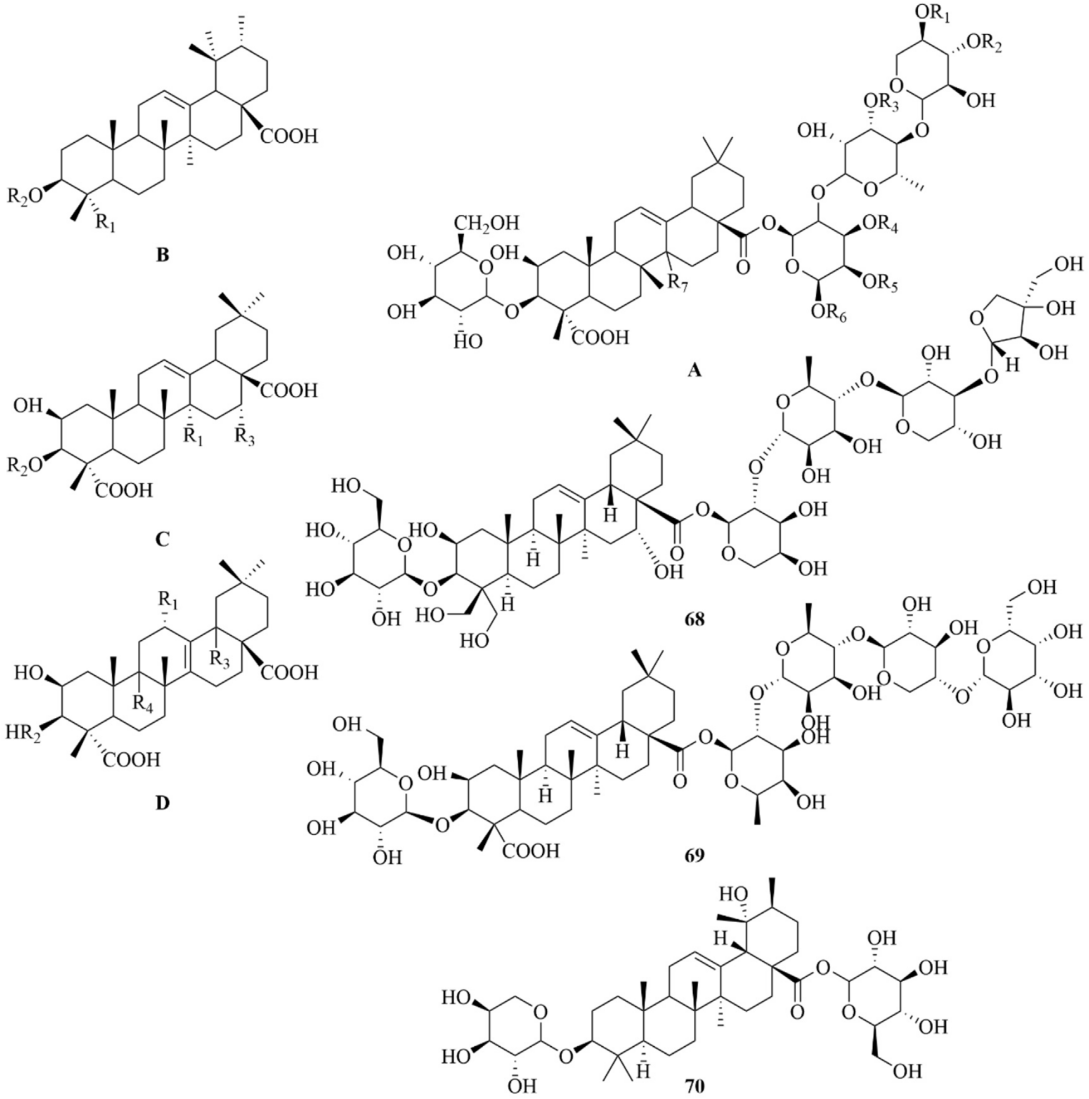
Structures of saponins [parent nucleus (A-D). 68–70] in PR.

**FIGURE 4:**
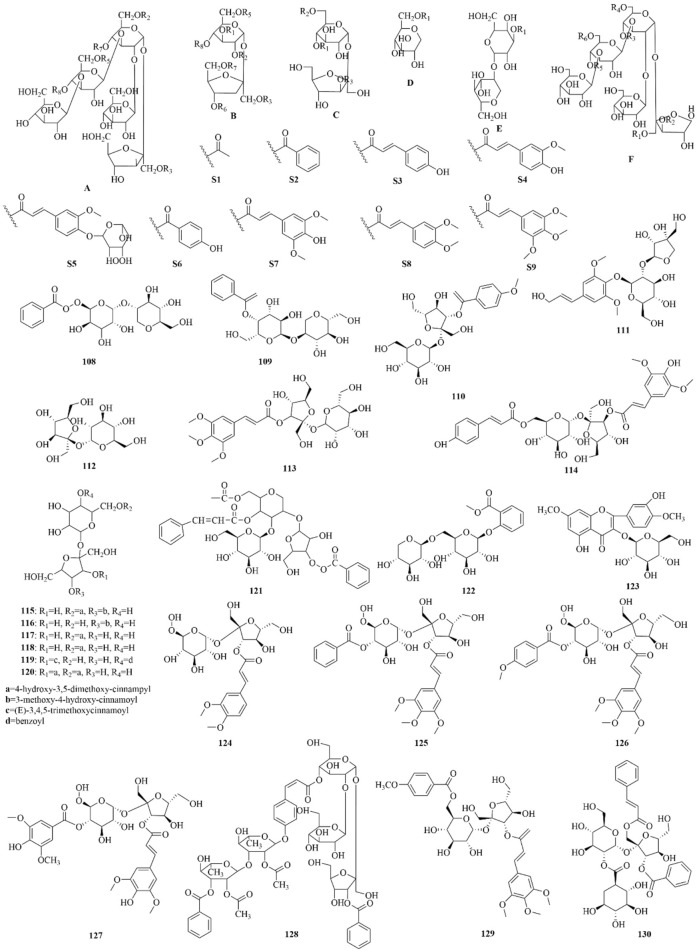
Structures of oligosaccharide esters [parent nucleus (A-F), substituent group (S1-S9), 108–130] in PR.

**FIGURE 5:**
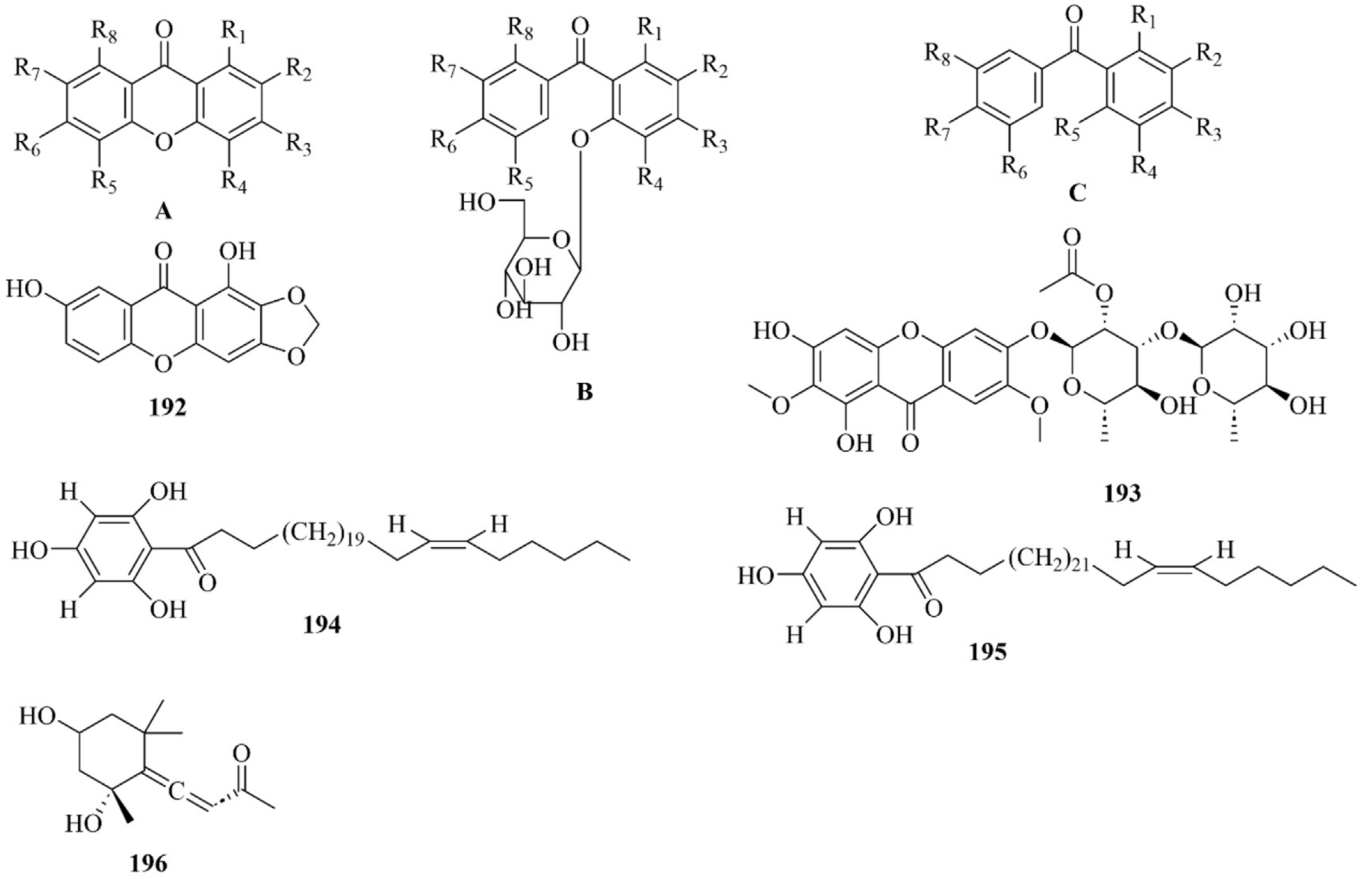
Structures of xanthones [parent nucleus (A-C), 192—196] in PR.

**FIGURE 6:**
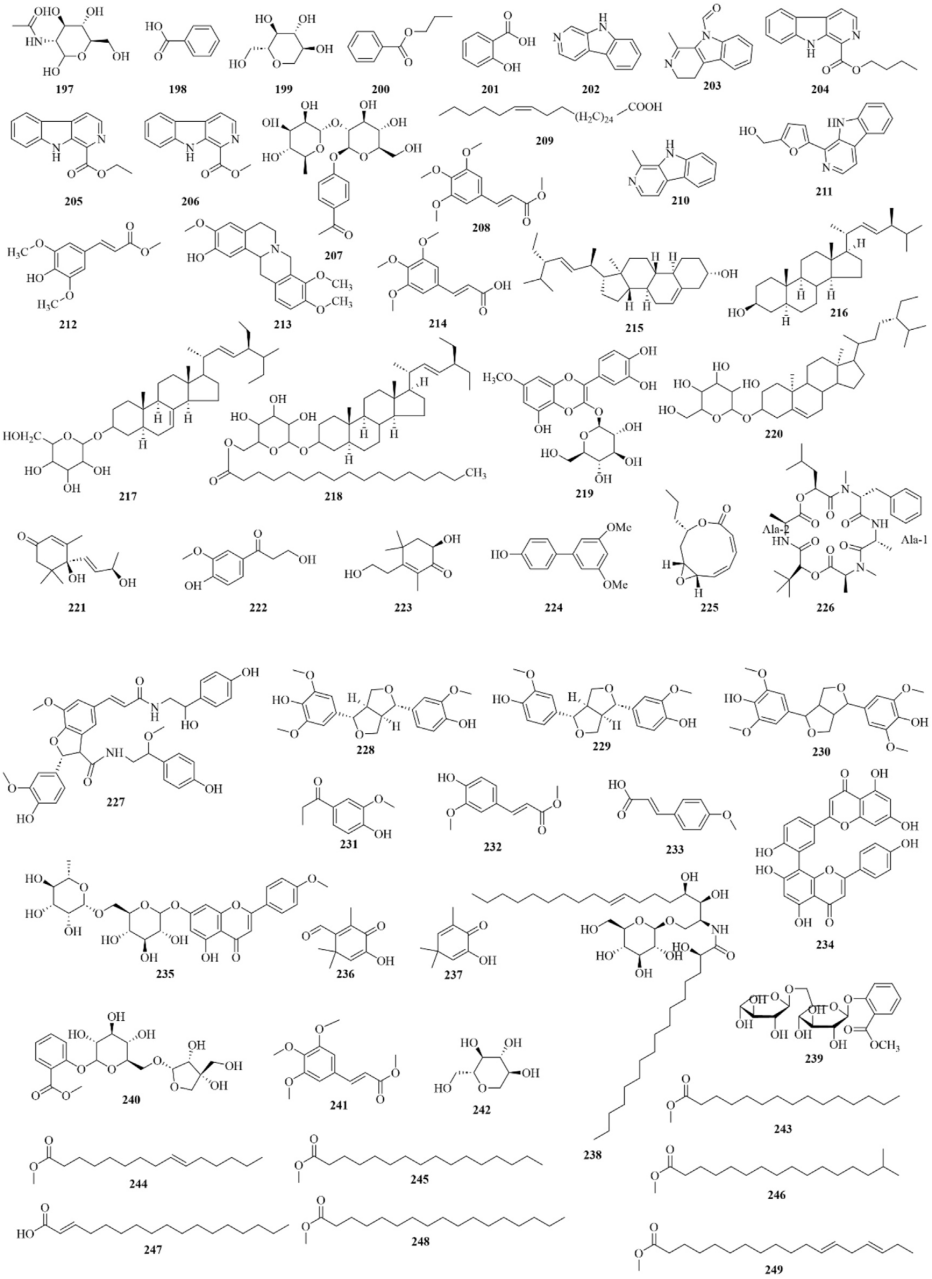
Structures of other constituents (197–249) in PR (1).

**FIGURE 7:**
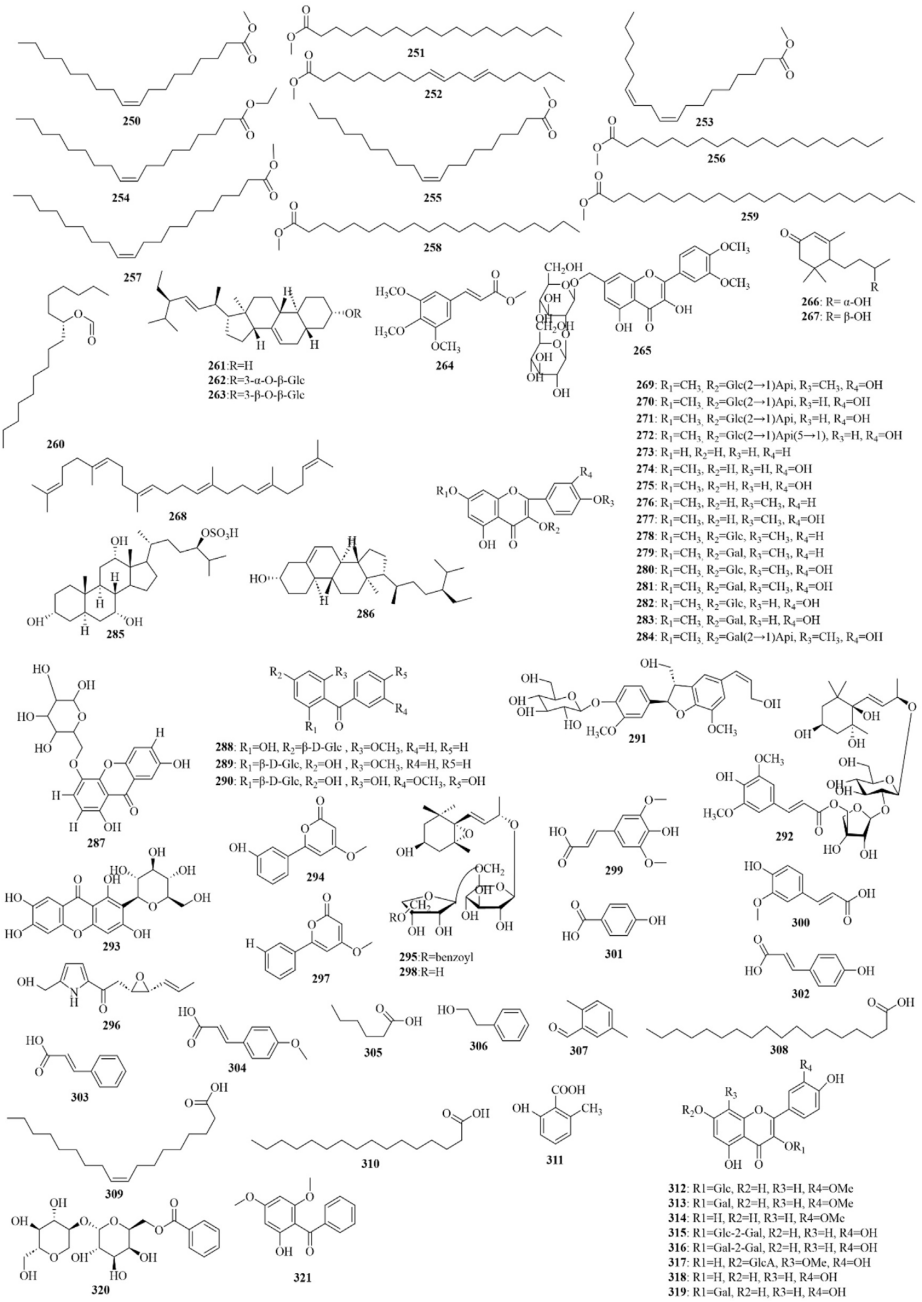
Structures of other constituents (250–321) in PR (2).

As early as 1947, researchers from Shanghai, China, isolated an amorphous acidic saponin from PR, which was hydrolyzed to obtain Tenuigenin A and B (CHOU et al., 1947). In 1964, two kinds of sugar, 5-anhydro-D-sorbitol and N-acetyl-D-glucosamine, were isolated from PR (Takiura and Honda, 1964). In 1977, 3,4,5-trimethoxycinnamic acid (TMCA), 1,2,3,7-tetramethoxyxanthone, 1,2,3,6,7-pentamethoxyxanthone, and 6-hydroxy-1,2,3,7-tetramethoxyxanthone were isolated from PR (Ito et al., 1977). At present, more than 320 metabolites have been isolated and identified from PR, including saponins, xanthones, oligosaccharides, and other metabolites. In this section, we summarize these metabolites and list them in Tables 1–3, and their structures can be seen in Figures 3–7.

**TABLE 1:** Saponins isolated from PR.

No.	Compound	Parent nucleus	R							Molecular formula	Ref.
			R_1_	R_2_	R_3_	R_4_	R_5_	R_6_	R_7_		
1	Onjisaponin A	A	Gal	H	Api	Rha	a	CH_3_	CH_2_OH	C_80_H_120_O_39_	Sakuma and Shoji (1982)
2	Onjisaponin E	A	Gal	H	H	H	b	CH_3_	CH_2_OH	C_71_H_106_O_33_	Sakuma and Shoji (1982)
3	Onjisaponin F	A	H	Ara	Api	H	b	CH_3_	CH_2_OH	C_75_H_112_O_36_	Sakuma and Shoji (1981)
4	Onjisaponin G	A	H	H	Api	H	b	CH_3_	CH_2_OH	C_70_H_104_O_32_	Sakuma and Shoji (1981)
5	Onjisaponin B	A	Gal	H	H	Rha	a	CH_3_	CH_2_OH	C_75_H_112_O_35_	Li (2008), Sakuma and Shoji (1982)
6	Onjisaponin D	A	H	H	Api	H	a	CH_3_	CH_2_OH	C_57_H_92_O_27_	Li (2008)
7	Onjisaponin J	A	H	Ara	e	Rha	a	COOH	CH_3_	C_85_H_126_O_42_	Li, 2008; Liu (2004)
8	Onjisaponin L	A	Gal	H	e	Rha	a	COOH	CH_3_	C_86_H_128_O_43_	Li, 2008; Liu (2004)
9	Onjisaponin O	A	Gal	H	H	Rha	b	COOH	CH_3_	C_77_H_116_O_37_	Li, 2008; Liu (2004)
10	Onjisaponin R	A	Gal	H	Api	H	b	COOH	CH_3_	C_76_H_114_O_37_	Li, 2008; Liu (2004)
11	Onjisaponin S	A	H	Ara	Api	H	b	COOH	CH_3_	C_81_H_122_O_40_	Li, 2008; Liu (2004)
12	Onjisaponin T	A	H	Ara	Api	Glc6d	b	COOH	CH_3_	C_83_H_124_O_42_	Li, 2008; Liu (2004)
13	Onjisaponin V	A	Gal	H	e	b	H	COOH	CH_3_	C_82_H_122_O_41_	Li, 2008; Li et al. (2008a)
14	Onjisaponin W	A	HM	Ara	e	b	H	COOH	CH_3_	C_81_H_120_O_40_	Li, 2008; Li et al. (2008b)
15	Onjisaponin X	A	H	Gal	e	Gal	b	COOH	CH_3_	C_87_H_130_O_45_	Li, 2008; Li et al. (2008a)
16	Onjisaponin Y	A	H	H	H	Rha	a	COOH	CH_3_	C_69_H_102_O_30_	Li, 2008; Li et al. (2008b)
17	Onjisaponin Z	A	H	H	H	Rha	b	COOH	CH_3_	C_71_H_106_O_2_	Li, 2008; Li et al. (2008a)
18	Onjisaponin Vg	A	Gal	H	e	H	b	COOH	CH_3_	C_82_H_122_O_41_	Li, 2008; Li et al. (2008b)
19	Onjisaponin Wg	A	H	Ara	Api	b	H	COOH	CH_3_	C_75_H_122_O_36_	Liu et al. (2007)
20	Onjisaponin Pg	A	Gal	H	e	H	H	CH_3_	CH_2_OH	C_76_H_120_O_41_	Liu et al. (2007)
21	Onjisaponin Gg	A	H	H	e	H	b	CH_3_	CH_2_OH	C_76_H_112_O_36_	Liu et al. (2007)
22	Onjisaponin Fg	A	H	Ara	e	H	b	CH_3_	CH_2_OH	C_81_H_120_O_40_	Liu et al. (2007)
23	Onjisaponin Qg	A	Gal	H	e	Rha	H	CH_3_	CH_2_OH	C_70_H_110_O_37_	Liu et al. (2007)
24	Onjisaponin Ng	A	H	H	e	Rha	a	CH_3_	CH_2_OH	C_80_H_118_O_38_	Liu et al. (2007)
25	Onjisaponin Sg	A	H	Ara	e	Rha	b	CH_3_	CH_2_OH	C_87_H_130_O_44_	Liu et al. (2007)
26	Onjisaponin Ug	A	H	Ara	e	Glc	b	CH_3_	CH_2_OH	C_87_H_130_O_45_	Liu et al. (2007)
27	Onjisaponin Tg	A	H	Ara	e	Glc6d	b	CH_3_	CH_2_OH	C_89_H_132_O_46_	Liu et al. (2007)
28	Denegins III	A	Gal	H	H	Rha	a	CH_3_	CH_2_OH	C_75_H_112_O_35_	Li et al., 2022a; Liu (2004)
29	Z-senegin III	A	Gal	H	H	Rha	f	CH_3_	CH_2_OH	C_75_H_112_O_35_	Li et al., 2022b; Liu (2004)
30	Z-senegin IV	A	Gal	H	Api	Rha	f	CH_3_	CH_2_OH	C_80_H_120_O_39_	Li et al., 2022a; Liu (2004)
31	(Z)-onjisaponin L	A	Gal	H	e	Rha	f	CH_3_	CH_2_OH	C_86_H_128_O_43_	Li et al., 2022b; Liu (2004)
32	(Z)-onjisaponin J	A	H	Ara	e	Rha	f	CH_3_	CH_2_OH	C_85_H_126_O_42_	Li et al., 2022a; Liu (2004)
33	E-onjisaponin H	A	H	H	Api	Rha	a	CH_3_	CH_2_OH	C_74_H_110_O_34_	Li et al. (2006)
34	Z-onjisaponin	A	H	H	Api	Rha	f	CH_3_	CH_2_OH	C_74_H_110_O_34_	Li et al. (2006)
35	Sibiricasaponin E	A	Glc	H	H	Api	COCH_3_	CH_3_	CH_3_	C_66_H_104_O_33_	Song et al. (2013a)
36	Tenuifolisaponin A	A	Gal6Oy	H	Api	Rha	a	CH_3_	CH_2_OH	C_85_H_126_O_42_	Sun (2005)
37	Tenuifolisaponin B	A	Gal6Oh	H	Api	Rha	a	CH_3_	CH_2_OH	C_86_H_130_O_41_	Sun (2005)
38	Polygalasaponin XXVIII	A	H	H	H	H	H	CH_3_	CH_2_OH	C_53_H_84_O_24_	Sun, 2005; Zhang et al. (1996)
39	Polygalasaponin XXIX	A	Api-Gal	H	H	H	H	CH_3_	CH_2_OH	C_64_H_102_O_33_	Zhang et al. (1996)
40	Polygalasaponin ⅩⅩⅩ	A	Gal	H	H	Glc	a	CH_3_	CH_2_OH	C_75_H_112_O_36_	Zhang et al. (1996)
41	Polygalasaponin XXXII	A	H	Ara	Api	Rha	a	CH_3_	CH_2_OH	C_79_H_118_O_38_	Liu, 2004; Sun (2005), Zhang et al. (1996)
42	Polygalasaponin XXIV	A	H	H	H	Api	H	CH_3_	CH_2_OH	C_58_H_92_O_28_	Zhang et al. (1996)
43	E-Senegasaponin A	A	Gal	H	Api	H	a	CH_3_	CH_2_OH	C_47_H_110_O_35_	Li et al. (2008a)
44	Arilloside A	A	H	H	H	d	d	CH_3_	CH_2_OH	C_57_H_72_O_27_	Song et al. (2013b)
45	Arilloside D	A	Gal	Ara	H	H	H	CH_3_	CH_2_OH	C_64_H_102_O_33_	Feng et al. (2019a)
46	Polygalasaponin LIII	A	H	Ara	Api	Glc6d	a	CH_3_	CH_2_OH	C_81_H_120_O_39_	Feng et al. (2019b)
47	Myrtifolioside A1	A	H	Gal	Api	Ara	a	CH_3_	CH_2_OH	C_79_H_118_O_39_	Feng et al. (2019a)
48	Compound 73	A	H	xyl	Api	Rha	a	CH_3_	CH_2_OH	C_79_H_118_O_38_	Lv (2007)
49	Compound 74	A	H	H	Api	H	H	CH_3_	CH_2_OH	C_58_H_92_O_28_	Lv (2007)
50	Onjisaponin MF	A	H	H	H	H	a	CH_3_	CH_2_OH	C_63_H_92_O_26_	Ling et al. (2013)
51	Onjisaponin TE	A	H	H	e	Rha	H	CH_3_	CH_2_OH	C_70_H_110_O_36_	Ling et al. (2013)
52	Onjisaponin TF	A	H	H	H	Rha	H	CH_3_	CH_2_OH	C_59_H_94_O_18_	Ling et al. (2013)
53	Onjisaponin TG	A	H	H	e	H	H	CH_3_	CH_2_OH	C_64_H_100_O_32_	Ling et al. (2013)
54	Onjisaponin TH	A	H	H	Api	H	b	CH_3_	CH_2_OH	C_65_H_96_O_28_	Ling et al. (2013)
55	Polygalasaponin XLV	A	Gal	H	H	Glc6d	c	CH_3_	CH_2_OH	C_76_H_116_O_38_	Feng et al. (2019a)
56	Sibiricasaponin A	B	COOH	g	—	—	—	—	—	C_36_H_53_O_12_	Song et al. (2013a)
57	Sibiricasaponin B	B	H	m	—	—	—	—	—	C_35_H_55_O_11_S	Song et al. (2013b)
58	Sibiricasaponin C	B	H	n	—	—	—	—	—	C_35_H_55_O_11_S	Song et al. (2013a)
59	Sibiricasaponin D	B	H	x	—	—	—	—	—	C_37_H_57_O_12_S	Song et al. (2013b)
60	Dehydroxypresenegenin	C	CH_3_	H	—	—				C_30_H_46_O_6_	Yan (2004)
61	Presenegenin	C	CH_2_OH	H	—	—				C_30_H_46_O_7_	Zhou et al. (2014)
62	Tenuifolin	C	CH_2_OH	Glc	—					—	C_36_H_56_O_12_
63	Polygalacic acid	C	—	H	OH					—	C_30_H_48_O_6_
64	Senegenic acid	D	—	H	—	H	—	—	—	C_29_H_44_O_6_	Zhang et al. (1996)
65	Fallaxsaponin A	D	—	Glc	—	—	—	—	—	C_35_H_54_O_11_	Liu et al. (2015)
66	Hydroxsenegennin	D	CH_2_OH	H	—	—	—	—	—	C_30_H_46_O_7_	Yan (2004)
67	Senegenin	D	CH_2_Cl	H	H	H	—	—	—	C_30_H_45_CIO_6_	Yan (2004)
68	Platycodin D									C_57_H_92_O_28_	Vinh et al. (2020)
69	Micranthoside A	—								C_57_H_92_O_28_	Vinh et al. (2020)
70	Zigu-glucoside I	—								C_41_H_66_O_13_	Song et al. (2012a)

Note: a = (E)-4-methoxy cinanamoyl; Api = β-D-apiofuranosyl; Ara = β-D-arabopyranosyl; b = (E)-3,4,5-trimethoxy cinnamoyl; c = (E)-3,4-methoxy cinanamoyl; d = acetyl; e = 3-hydroxy-3-mrthylmethyl-5-pentanoic acid ester-5-β-D-apiofur-anosyl; f = (Z)-4-methoxy cinnamoyl; g = D-glucopyranuronic acid; Gal = β-D-galactopyranosyl; Glc = β-D-glucopyranosyl; h = 2-hydroxy-4-methyl-pentanoic acid ester-6-β-D-gal; m = α-D-xyl-4-SO3H; n = β-D-xyl-5- SO3H; Rha = α-L-rhamnopyranosyl; x = α-D-xyl-3-acetoxy-4-SO3H; y = 4-oxide-2-hydroxy-pentanoic acid ester-6-β-D-gal.

**TABLE 2:** Xanthones isolated from PR.

No.	Compound	Parent nucleus	R								Formula	Ref.
			R_1_	R_2_	R_3_	R_4_	R_5_	R_6_	R_7_	R_8_		
131	Lancerin	A	H	H	OH	H	H	OH	Glc	H	C_19_H_18_O_10_	Liu et al. (2010), Zhou et al. (2014)
132	Onjixanthone I	A	OMe	OMe	OMe	H	H	H	OH	H	C_16_H_14_O_6_	Yukinobu et al. (1991)
133	Onjixanthone II	A	OH	OMe	OH	H	H	OH	OMe	H	C_15_H_12_O_7_	Yukinobu et al. (1991), Zhou et al. (2014)
134	Polygalaxanthone III	A	OH	Glc-6-Api	OH	H	H	OH	OMe	H	C_25_H_28_O_15_	Ikeya et al. (1994)
135	Polygalaxanthone IV	A	OH	H	OMe	—	—	Oglc-2-1Rha	OMe	H	C_27_H_32_O_15_	Jiang et al. (2011)
136	Polygalaxanthone V	A	OH	H	OH	—	—	Oglc-2-1Rha	OMe	H	C_26_H_30_O_15_	Zhou et al. (2014)
137	Polygalaxanthone VI	A	OMe	OMe	OMe	—	—	OGlc	OMe	H	C_23_H_26_O_2_	Zhou et al. (2014)
138	Polygalaxanthone VII	A	OH	OMe	OGlc2-Rha	OH	OMe	H	H	H	C_27_H_32_O_16_	Wang et al. (2005)
139	Polygalaxanthone VIII	A	OH	Glc-6-Ara	OH	H	H	OH	OMe	H	C_25_H_28_O_15_	Jiang et al. (2005)
140	Polygalaxanthone IX	A	OH	H	OGlc-2-Rha	H	H	H	OH	H	C_25_H_28_O_14_	Jiang et al. (2005)
141	Polygalaxanthone X	A	OMe	OMe	OMe	H	H	OGlc-2-Rha	OMe	H	C_29_H_36_O_16_	Jiang et al. (2005)
142	Sibiricaxanthone A	A	OH	Glc6-Api	OH	H	H	H	OH	H	C_24_H_26_O_14_	Miyase et al. (1999)
143	Sibiricaxanthone B	A	OH	Glc2-1Rha	OH	H	H	H	OH	H	C_24_H_26_O_14_	Miyase et al. (1999)
144	7-Phenyl-1-hydroxy-2,3,6-trimethoxyxanthone	A	OH	OCH_3_	OCH_3_	—	—	OCH_3_	—	phenyl	C_22_H_18_O_6_	Jia et al. (2023)
145	1,2,3-Trimethoxy-7-hydroxyxanthone	A	OMe	OMe	OMe	H	H	H	OH	H	C_16_H_14_O_6_	Li et al. (2022b)
146	1,3,6-Trihydroxy-2,7-dimethoxyxanthone	A	OH	OMe	OH	H	H	OH	OMe	H	C_15_H_12_O_7_	Zhang et al. (2016)
147	1,2,7-Trimethoxy-3-hydroxyxanthone	A	OMe	OMe	OH	H	H	H	OMe	H	C_16_H_14_O_6_	Li et al. (2022a)
148	1,2,3,7-Tetramethoxyxanthone	A	OMe	OMe	OMe	H	H	H	OMe	H	C_17_H_16_O_6_	Yukinobu et al. (1991)
149	1,7-Dihydroxy-3-methoxyxanthone	A	OH	H	OMe	H	H	H	OH	H	C_14_H_10_O_5_	Liu et al. (2010)
150	1,7-Dihydroxy-2,3-dimethoxyxanthone	A	OH	OMe	OMe	H	H	H	OH	H	C_15_H_12_O_6_	Wang et al. (2005)
151	1,7-Dihydroxy-2,3-methlendioxyyxanthone	A	OMe	OCH_2_O	OCH_2_O	H	H	H	H	OMe	C_14_H_8_O_6_	Jiang and Tu (2004)
152	6-Hydroxy-2,3,6,7-tetramethoxyxanthone	A	H	OMe	OMe	H	H	OMe	OMe	H	C_17_H_16_O_7_	Zhang et al. (2016)
153	6-Hydroxy-1,2,3,7-tetramethoxyxanthone	A	OMe	OMe	OMe	H	H	OGlc	OMe	H	C_17_H_16_O_7_	Yukinobu et al. (1991), Zhou et al. (2014)
154	1,7-Dihydroxyxanthone	A	OH	H	H	H	H	H	OH	H	C_13_H_8_O_4_	Jiang and Tu (2004)
155	1,7-Dimethoxyxanthone	A	OMe	H	H	H	H	H	OMe	H	C_15_H_12_O_4_	Li (2008)
156	1-Hydroxy-3,7-dimethoxyxanthone	A	OH	H	OMe	H	H	H	OMe	H	C_15_H_12_O_5_	Jiang and Tu (2004)
157	1-Hydroxy-3,6,7-trimethoxyxanthone	A	OH	H	OMe	H	H	OMe	OMe	H	C_16_H_14_O_6_	Jiang and Tu (2004)
158	6,8-Dihydroxy-12,4-trimethoxyxanthone	A	OMe	OMe	H	OH	H	H	H	OH	C_16_H_14_O_7_	Fujita et al. (1992)
159	6,8-Dihydroxy-1,2,3-trimethoxyxanthone	A	OMe	OMe	OMe	H	H	H	H	OH	C_16_H_14_O_7_	Fujita et al. (1992)
160	3-Hydroxy-2,8-dimethoxyxanthone	A	H	OMe	OH	H	H	H	H	OMe	C_15_H_12_O_5_	Yukinobu et al. (1991)
161	1,2,3,6,7-Pentamethoxyxanthone	A	OMe	OMe	OMe	H	H	OMe	OMe	H	C_18_H_18_O_7_	Jiang et al. (2003), Zhou et al. (2014)
162	1,3,7-Trihydroxyxanthone	A	OH	H	OH	H	H	H	OH	H	C_13_H_8_O_5_	Jiang et al. (2003)
163	1,6,7-Trihydroxy-2,3-dimethoxyxanthone	A	OH	OMe	OMe	H	H	OH	OH	H	C_15_H_12_O_7_	Jiang et al. (2003)
164	3-Hydroxy-1,2,7-trimethoxyxanthone	A	OMe	OMe	OH	H	H	H	OMe	H	C_16_H_14_O_6_	Fujita et al. (1992)
164	7-Hydroxy-1,2,3-trimethoxyxanthone	A	OMe	OMe	OMe	H	H	H	OH	H	C_16_H_14_O_6_	Zhang et al. (2016)
165	2,3,8-Trimethoxyxanthone	A	H	OMe	OMe	H	H	H	H	OMe	C_16_H_14_O_5_	Fujita et al. (1992)
166	1,3,6,7-Tetramethoxyxanthone	A	OMe	H	OMe	H	H	OMe	OMe	H	C_17_H_16_O_6_	Fujita et al. (1992)
167	1,3,7-Trimethoxyxanthone	A	OMe	H	OMe	H	H	H	OMe	H	C_16_H_14_O_5_	Jiang et al. (2003)
168	7-O-Methylmangiferin	A	OH	Glc	OH	H	H	OH	OMe	H	C_20_H_20_O_11_	Liu et al. (2010)
169	Euxanthone	A	OH	H	H	H	H	H	OH	H	C_13_H_8_O_4_	Yang et al. (2000)
170	1,6-Dihydroxy-3,7-dimethoxyxanthone	A	OH	H	OCH_3_	H	H	OH	OCH_3_	H	C_15_H_12_O_6_	Yukinobu et al. (1991)
171	1,6-Dihydroxy-3,5,7-trimethyoxyxanthone	A	OH	H	OCH_3_	H	OCH_3_	OH	OCH_3_	H	C_16_H_14_O_7_	Miyase and Ueno (1993)
172	Irisxanthone	A	H	H	OH	OCH_3_	H	OH	Glc	OH	C_20_H_20_O_11_	Chai et al. (2018)
173	Polygalaxanthone Ⅺ	A	OH	Glc-2-Api	OH	H	H	OH	OCH_3_	H	C_25_H_28_O_15_	Li (2008)
174	1,6-Dimethoxy-2,3-methylenedioxyxanthone	A	OCH_3_	OCH_2_O	OCH_2_O	H	H	H	OCH_3_	H	C_16_H_12_O_6_	Li (2008)
175	1,3-Dihydroxy-5,6,7-trimethoxyxanthone	A	OH	H	OH	H	OCH_3_	OCH_3_	OCH_3_	H	C_16_H_14_O_7_	Li (2008)
176	7-Hydroxy-1-methoxy-2,3-methylenedioxyxanthone	A	OCH_3_	OCH_2_O	OCH_2_O	H	H	H	OH	H	C_15_H_10_O_6_	Li (2008)
177	1,2,7-Trihydroxy-3,6-dimethoxyxanthone	A	OH	OH	OCH_3_	H	H	OCH_3_	OH	H	C_15_H_12_O_7_	Li (2008)
178	4-C-β-D-Glucopyrabosyl-1,3,6-trihydroxy-7-methoxyxanthone glycosides	A	OH	H	OH	β-D-Glc	H	OH	OCH_3_	H	C_20_H_20_O_11_	Wang et al. (2003)
179	4-C-[β-D-apiofuranosyl-(1→6) -β-D-lucopyrabosyl]-1,3,6-trihydroxy-7-methoxyxanthone glycosides	A	OH	H	OH	β-D-Glc-β-D-Api	H	OH	OCH_3_	H	C_25_H_28_O_6_	Wang et al. (2003)
180	1-Hydroxy-7-methoxyxanthone	A	OH	H	H	—	—	H	OCH_3_	—	C_14_H_10_O_4_	Cho et al. (2012)
181	3,6-Dihydroxy-1,2,7-trimethoxyxanthone	A	OCH_3_	OCH_3_	OH	OH	—	OH	OCH_3_	—	C_16_H_14_O_7_	Cho et al. (2012)
182	1,2,3-Trihydroxy-6,7-dimethoxyxanthone	A	OOH	OH	OH	OH	—	OOH	—	—	C_13_H_8_O_9_	Yoo et al. (2014)
183	1,2,3,6,7-Pentahydroxyxanthone	A	OH	OCH_3_	OCH_3_	OOH	—	OH	—	—	C_15_H_12_O_3_	Yoo et al. (2014)
184	4-Hydroxy-1,2,3,7-tetramethoxyxanthone	A	OOH	OCH_3_	OCH_3_	OOH	—	OOH	—	—	C_15_H_12_O_10_	Yoo et al. (2014)
185	1,3,7-Trihydroxy-2,6-dimethanoxyxanthone	A	OH	OMe	OH	—	—	MeO	OH	—	C_15_H_12_O_7_	Xu et al. (2014)
186	7-Hydroxy-1-methoxyxanthone	A	OMe	H	H	H	—	H	H	OH	C_14_H_10_O_4_	Xu et al. (2014)
187	1,7-Dihydroxy-3, 4-dimethoxyxanthone	A	OH	—	OCH_3_	OCH_3_	—	—	OH	—	C_15_H_12_O_6_	Xu et al. (2014)
188	Sibiriphenone A	B	OH	H	OH	H	H	OH	OMe	H	C_20_H_22_O_11_	Zhou et al. (2014)
189	2-Hydroxy-4,6-dimethoxybenzophenone	C	OH	H	OCH_3_	H	OCH_3_	H	H	H	C_15_H_14_O_4_	Liu et al. (2021)
190	Tenuiphenone A	C	OH	H	OCH_3_	H	β-D-Glc-α-L-rha-	H	H	H	C_26_H_32_O_13_	Jiang and Tu (2005)
191	Tenuiphenone B	C	OH	β-D-Glc	OH	β-D-Glc	OH	OH	H	H	C_25_H_30_O_17_	Jiang and Tu (2005)
192	1,7-Dihydroxy-2,3-methylenedioxyxanthone										C_14_H_8_O_6_	Shi et al. (2013a)
193	Sibiricaxanthone F										C_29_H_34_O_16_	Cho et al. (2012)
194	Tenuiphenone C										C_36_H_62_O_4_	Jiang and Tu (2005)
195	Tenuiphenone D										C_38_H_66_O_4_	Jiang and Tu (2005)
196	Grasshopper ketone										C_13_H_20_O_3_	Tan et al. (2022)

**TABLE 3:** Other components isolated from PR.

No.	Compound	Molecular formula	Ref.
197	N-acetyl-D-glucosamine	C_8_H_15_NO_6_	Wang and Xia (2012)
198	Benzoic acid	C_7_H_6_O_2_	Wang et al. (2005)
199	1,5-Anhydro-D-sorbitol	C_6_H_12_O_5_	Pei et al. (2005)
200	Propyl benzoate	C_10_H_12_O_2_	Li et al. (2005)
201	O-Hydroxybenzoic acid	C_7_H_6_O_3_	Jiang et al. (2011)
202	Norharman	C_11_H_8_N_2_	Jin and Park (1993)
203	N_9_-formylharman	C_13_H_12_N_2_O	Jin and Park (1993)
204	1-Carbobutoxy-β-carboline	C_13_H_16_N_2_O_2_	Jin and Park (1993)
205	1-Carboethoxy-β-carboline	C_14_H_12_N_2_O_2_	Jin and Park (1993)
206	1-Carbomethoxy-β-carboline	C_13_H_10_N_2_O_2_	Jin and Park (1993)
207	Sibiricaphenone	C_20_H_28_O_11_	Miyase et al. (1999)
208	(28Z)-Tetratriacontenoic acid	C_33_H_66_O_2_	Jiang et al. (2003)
209	3,4,5-Trimethoxybenzeneacrylic acid methyl ester	C_13_H_16_O_5_	Li et al. (2005)
210	Harman	C_12_H_10_N_2_	Jin and Park (1993)
211	Perlolyrine	C_16_H_12_N_2_O_2_	Jin and Park (1993)
212	4-Hydroxy-3,5-dimethoxylcinnamate	C_12_H_14_O_5_	Jiang et al. (2011)
213	Tetrahydrocolumbamine	C_20_H_23_NO_4_	Shen et al. (2004)
214	3,4,5-Trimethoxycinnamic acid	C_12_H_14_O_5_	Li et al. (2005), Zhou et al. (2014)
215	Stigmasterol	C_29_H_48_O	Jiang et al. (2003)
216	Α-Sipnasterol	C_29_H_48_O	Jiang et al. (2003)
217	Α-Sipnasterol-3-O-β-D-glucoside	C_35_H_58_O_6_	Jiang et al. (2003)
218	Α-Sipnasterol-3-O-β-D-glucoside-6′-O-paltimate	C_51_H_88_O_7_	Jiang et al. (2003)
219	Rhamnetin-3-O-β-D-glucopyranoside	C_22_H_22_O_12_	Zhou et al. (2014)
220	Β-Daucosterin	C_35_H_60_O_6_	Wang et al. (2003)
221	Vomifoliol	C_13_H_20_O_3_	Tan et al. (2022)
222	3-Hydroxy-1-(4-hydroxy-3-methoxyphenyl)-propan-1-one	C_10_H_12_O_4_	Tan et al. (2022)
223	Robinlin	C_33_H_40_O_19_	Tan et al. (2022)
224	3′,5′-Dimethoxy-biphenyl-4-ol	C_14_H_14_O_3_	Tan et al. (2022)
225	Phomolide A	C_12_H_16_O_3_	Tan et al. (2022)
226	Spicellamide A	C_32_H_48_N_4_O_8_	Tan et al. (2022)
227	Tenuifolimide A	C_37_H_38_N_2_O_10_	Chen et al. (2022b)
228	(−)-Medioresinol	C_21_H_24_O_7_	Cheng et al. (2022a)
229	(+)-Pinoresinol	C_20_H_22_O_6_	Cheng et al. (2022a)
230	Syringaresinol	C_22_H_26_O_8_	Cheng et al. (2022a)
231	4-Hydroxy-3-methoxypropiophenone	C_10_H_12_O_3_	Cho et al. (2012)
232	Methyl 4-hydroxy-3-methoxycinnamic acid	C_11_H_12_O_4_	Cho et al. (2012)
233	4-Methoxycinnamic acid	C_10_H_10_O_3_	Cho et al. (2012)
234	Amentoflavon	C_30_H_18_O_10_	Cho et al. (2012)
235	Linarin	C_28_H_32_O_14_	Cho et al. (2012)
236	2,4,4-Trimethyl-3-formyl-6-hydroxy-2,5-cyclohexadien-1-on	C_10_H_12_O_3_	Cho et al. (2012)
237	Lanierone	C_9_H_12_O_2_	Cho et al. (2012)
238	Aralia cerebroside	C_40_H_77_NO_10_	Cho et al. (2012)
239	1-O-L-arabinopyranosyl-O-(6→1)-β-D-glucopyranosyl-salicylate	C_19_H_27_O_12_	Zhou et al. (2008)
240	Canthoside A	C_19_H_26_O_20_	Zhou et al. (2008)
241	Methyl 3,4,5-trimethoxycinnamate	C_13_H_16_O_5_	Zhou et al. (2008)
242	1,5-Anhydro-D-glucitol	C_6_H_12_O_5_	Yu et al. (2014)
243	Methyl pentadecanoate	C_16_H_32_O_2_	Sun et al. (2000)
244	Methyl 9-hexadecenoate	C_17_H_32_O_2_	Sun et al. (2000)
245	Methyl hexadecanoate	C_17_H_34_O_2_	Sun et al. (2000)
246	Methyl isoheptadecanoate	C_18_H_36_O_2_	Sun et al. (2000)
247	Heptadecenoic acid	C_18_H_34_O_2_	Sun et al. (2000)
248	Methyl heptadecanoate	C_18_H_36_O_2_	Sun et al. (2000)
249	Methyl 12,15-octadecadienoate	C_19_H_34_O_2_	Sun et al. (2000)
250	Methyl cis-9-octadecenoate	C_19_H_36_O_2_	Sun et al. (2000)
251	Methyl octadecanoate	C_19_H_38_O_2_	Sun et al. (2000)
252	9,12-Octadecadienoic acid, methyl ester, (E, E)-	C_19_H_34_O_2_	Sun et al. (2000)
253	9,12-Octadecadienoic acid, methyl ester, (Z, Z)-	C_19_H_34_O_2_	Sun et al. (2000)
254	Ethyl oleate	C_20_H_38_O_2_	Sun et al. (2000)
255	(Z)-9-Nonadecenoic acid, methyl ester	C_20_H_38_O_2_	Sun et al. (2000)
256	Methyl nonadecanoate	C_20_H_40_O_2_	Sun et al. (2000)
257	(Z)-Methyl icos-11-enoate	C_21_H_40_O_2_	Sun et al. (2000)
258	Methyl arachidate	C_21_H_42_O_2_	Sun et al. (2000)
259	Methyl behenate	C_23_H_46_O_2_	Sun et al. (2000)
260	Poligapolide	C_18_H_34_O_2_	Yoo et al. (2014)
261	Chondrillasterol	C_29_H_48_O	Yoo et al. (2014)
262	3α-O-β-Pyranoglucosyl spinasterol	C_29_H_46_O_2_	Yoo et al. (2014)
263	3β-O-β-Pyranoglucosyl Chondrillasterol	C_35_H_57_O_5_	Yoo et al. (2014)
264	3,4,5-Trimethoxy methyl cinamate	C_13_H_16_O_5_	Yoo et al. (2014)
265	3′,4′-Dimethoxy-7-diglucosyl-O-methylenoxy-5-hydroxyl flavol	C_30_H_36_O_17_	Yoo et al. (2014)
266	Blumenol C	C_13_H_22_O_2_	Son et al. (2022)
267	9-Epi-blumenol C	C_13_H_22_O_2_	Son et al. (2022)
268	Squalene	C_30_H_50_	Song et al. (2009)
269	Polygalin D	C_28_H_32_O_16_	Song et al. (2013a)
270	Polygalin E	C_27_H_30_O_16_	Song et al. (2013b)
271	Polygalin F	C_27_H_30_O_16_	Song et al. (2013a)
272	Polygalin G	C_33_H_40_O_21_	Song et al. (2013b)
273	Kaempferol	C_15_H_10_O_6_	Song et al. (2013a)
274	Rhamnocitrin	C_16_H_12_O_7_	Song et al. (2013b)
275	Rhamnetin	C_16_H_12_O_7_	Song et al. (2013a)
276	Ermanin	C_17_H_14_O_6_	Song et al. (2013b)
277	Ombuine	C_17_H_14_O_7_	Song et al. (2013a)
278	Ermanin-3-O-β-D-glucopyranoside	C_22_H_24_O_12_	Song et al. (2013b)
279	Polygalin A	C_23_H_24_O_11_	Song et al. (2013a)
280	Ombuine-3-O-β-D-glucopyranoside	C_23_H_24_O_12_	Song et al. (2013b)
281	Ombuine-3-O-β-D-galactopyranoside	C_23_H_24_O_12_	Song et al. (2013a)
282	Rhamnocitrin-3-O-β-D-glucopyranoside	C_22_H_22_O_12_	Song et al. (2013b)
283	Rhamnocitrin-3-O-β-D-galactopyranoside	C_22_H_22_O_12_	Song et al. (2013a)
284	Polygalin C	C_28_H_32_O_16_	Song et al. (2013b)
285	Polygalasterol A	C_27_H_47_O_7_S	Song et al. (2012b)
286	Clionosterol	C_29_H_50_O	Le et al. (2012)
287	Tenuiside A	C_19_H_18_O_10_	Shi et al. (2013b)
288	Tenuiside B	C_20_H_22_O_9_	Shi et al. (2013b)
289	Tenuiside C	C_20_H_22_O_9_	Shi et al. (2013b)
290	Tenuiside D	C_20_H_22_O_11_	Shi et al. (2013b)
291	Tenuiside E	C_26_H_32_O_11_	Shi et al. (2013b)
292	Tenuiside F	C_35_H_52_O_17_	Shi et al. (2013b)
293	Mangiferin	C_19_H_18_O_11_	Shi et al. (2013b)
294	Polygalapyrone A	C_12_H_10_O_4_	Cheng et al. (2022b)
295	Tenuiside G	C_31_H_44_O_13_	Cheng et al. (2022b)
296	Polygalapyrrole A	C_12_H_15_NO_3_	Cheng et al. (2022b)
297	5-Methoxy-6-phenyl-2H-pyran-2-one	C_12_H_10_O_3_	Cheng et al. (2022b)
298	Premnaionoside	C_24_H_40_O_12_	Cheng et al. (2022b)
299	Sinapic acid	C_11_H_12_O_5_	Song et al. (2016)
300	Ferulic acid	C_10_H_10_O_4_	Song et al. (2016)
301	P-Hydroxybenzoic acid	C_7_H_6_O_3_	Song et al. (2016)
302	P-Coumaric acid	C_9_H_8_O_3_	Song et al. (2016)
303	Cinnamic acid	C_9_H_8_O_2_	Song et al. (2016)
304	P-Methoxy cinnamic acid	C_7_H_6_O_3_	Song et al. (2016)
305	Hexanoic acid	C_6_H_12_O_2_	Wu (2010)
306	Phenethyl alcohol	C_8_H_10_O	Wu (2010)
307	2,5-Dimethylbenzaldehyde	C_9_H_10_O	Wu (2010)
308	Stearic acid	C_18_H_36_O_2_	Wu (2010)
309	Oleic acid	C_18_H_34_O_2_	Wu (2010)
310	Palmitic acid	C_16_H_12_O_2_	Wu (2010)
311	Methylsalicylic acid	C_8_H_8_O_3_	Wu (2010)
312	Isorhamnetin-3-O-β-D-glucopyranoside	C_22_H_22_O_12_	Shi et al. (2013a)
313	Isorhamnetin-3-O-β-D-galactopyranoside	C_22_H_22_O_12_	Shi et al. (2013a)
314	Isorhamnetin	C_16_H_12_O_7_	Shi et al. (2013a)
315	Quercetin-3-O-β-D-glucopyranosyl (1→2)-β-D-galactopyranoside	C_28_H_32_O_17_	Shi et al. (2013a)
316	Quercetin-3-O-β-D-glucopyranosyl (1→2)-β-D- glucopyranoside	C_28_H_32_O_17_	Shi et al. (2013a)
317	5,7-Dihydroxy-8-methoxyflavone-7-O-β-D-glucuronoside	C_22_H_20_O_14_	Shi et al. (2013a)
318	Quercetin	C_15_H_10_O_7_	Shi et al. (2013a)
319	Quercetin-3-O-β-D- glucopyranoside	C_21_H_20_O_12_	Shi et al. (2013a)
320	Roseoside Ⅱ	C_19_H_30_O_8_	Tan et al. (2022)
321	Garcimangosone D	C_19_H_20_O_9_	Tan et al. (2022)
322	Ethyl cholestan-22-enol	C_29_H_50_O	Le et al. (2012)
323	3β-O-β-D-Glucopyranosyl ethyl cholestan-22-enol	C_35_H_60_O_6_	Le et al. (2012)
324	3-O-β-D-Glucopyranosyl clionasterol	C_29_H_50_O	Le et al. (2012)

### Saponins

Saponins are the most important active metabolites of PR and are associated with a wide range of potent pharmacological effects. To date, 70 saponins with oleanane-type pentacyclic triterpene saponins as the parent nucleus, usually linked to glucose, rhamnose, lactose, xylose, galactose, and other sugar types, have been isolated from PR (As shown in Table 1; Figure 3) (Chen and Zhang, 2019). Saponins are unstable and can easily decompose into senegenin, polygalacic acid, tenuifolin, and polygalaxanthone III (PX3) when meeting acid (Gao et al., 2022), possibly explaining the few reports of the monomer. Modern research proves that PR saponins have multiple pharmacological effects, including enhancing immunity, protecting vision, anti-tumor, anti-cardiovascular, and anti-AD (Gao et al., 2022). TLC identification and content determination based on tenuifolin are important methods for the quality control of PR.

### Oligosaccharide esters

Oligosaccharide esters in PR are a class of metabolites mainly based on sucrose, glucose, or other trisaccharide small molecules as the parent nucleus. They form oligosaccharides by linking with rhamnose or glucose through various glycosidic bonds and then further form esters with organic acid constituents such as acetic acid, benzoic acid, and phenylpropenoic acid. They have no more than five sugar molecules in their molecule. Modern research shows that oligosaccharide esters of PR have neuroprotective, antidepressant, anti-dementia, and other central pharmacological effects (Sang et al., 2017). 3′6-Disinapoyl sucrose is another quality control indicator of PR in the *Chinese Pharmacopoeia*. Oligosaccharide esters isolated and identified from PR are listed and shown in Supplementary Table 5; Figure 4.

### Xanthones

Xanthone is a general term for secondary metabolites with a tricyclic aromatic hydrocarbon system structure, also known as benzophenanthrone and dibenzo-γ-pyrone. About 70 xanthones have been identified from PR, whose parent nucleus is composed of a benzene ring paralleled with a chromone and has eight substitution sites. Xanthones in PR are mainly simple ketones, with common substituents including hydroxyl, methoxy, and methylenedioxy groups (Wang et al., 2020b). PX3 is one of the index metabolites for content determination of PR in the *Chinese Pharmacopoeia* 2020 edition. The xanthones obtained from PR are shown in Table 2; Figure 5.

### Others

Alkaloids, organic acids, volatile oils, coumarins, phenylpropanoids, and steroidals are also present in PR (Zhao et al., 2020), as shown in Table 3; Figures 6, 7. PR also contains trace elements (Li, 2008), such as Zn, Cu, Fe, Mn, K, Ca, and Mg.

Nowadays, studies of metabolite separation from the roots of PR have gradually decreased, and the existing research mostly focuses on saponins and oligosaccharide esters. The attention to xanthones and other metabolites should be improved. This may be related to the relatively low content of these metabolites, and optimizing the technical of extraction, enrichment, and purification may improve the situation. The aerial parts of PR, which have been discarded as waste, are beginning to receive attention and show potential. Tan et al. adopted silica gel, a medium-pressure octadecylsilyl (ODS) chromatographic column, and high-performance liquid chromatography to separate and purify the n-butanol part of the 70% ethanol extract of the aerial part of PR from 13 metabolites of which nine metabolites, including saponin metabolite II, were isolated from plants of *Polygalaceae* for the first time (Tan et al., 2022). Further studies are needed to investigate the differences in chemical composition and content between the aerial parts and roots of PR to further guide their rational development and utilization. Furthermore, the structure-activity relationship of active substances in PR has not been fully revealed; future research is needed to guide the development of lead metabolites, which will help to synthesize or semi-synthesize new derivatives with high efficiency, low toxicity, and safety. In addition, the current extraction and separation technologies mainly use organic reagents such as n-butanol, ethyl acetate, petroleum, and so on, which are irritating to the human body and harmful to the environment. With the advancement of analytical methods and techniques, it is believed that more advanced and environmentally friendly technologies and instruments will be applied.

## Pharmacology

Modern pharmacological studies have shown that PR has significant pharmacological activity against nervous system diseases and has anti-oxidation, anti-inflammatory, anti-tumor, and anti-pathogenic microorganism functions, as well as other effects (Zhou et al., 2022), as shown in Supplementary Table 6.

### Anti-neurodegenerative effects

#### Anti-pathologic protein aggregation effects

PR can improve the transport and clearance of Aβ by up-regulating the protein expression of low-density lipoprotein receptor-related protein 1, promoting p21, and down-regulating the receptors for advanced glycation end products and cyclinD1 and E2F1 protein expressions. At the same time, it can inhibit caspase-3, caspase-8, and Bax expression and increase Bcl-2 expression (Yu et al., 2022). The expression of two main enzymes involved in Aβ degradation, insulin-degrading enzyme (IDE) and neprilysin (NEP), can be enhanced by a pectin polysaccharide named RP02-1. RP02-1 can also inhibit Aβ aggregation, and its effect on Aβ aggregation and production made it a candidate leading metabolite for AD (Zeng et al., 2020a). Polygala tenuifolia polysaccharide, another important polysaccharide from PR, reduces Aβ deposition, activates extracellular-signal-regulated kinase (ERK) pathway-related proteins, and enhances synaptic plasticity, which allows it to improve spatial cognitive deficits in AD mice to reduce cellular damage in the hippocampal CA3 region and to maintain the balance of the cholinergic system, thus exerting an anti-AD effect *in vivo* (Li et al., 2024). It is necessary to reveal their structure-activity relationship and study safety for structural modification and optimization as well as for the development of new drugs. 3,6′-Disinapoyl sucrose (DISS) is an oligosaccharide ester of PR that can regulate the mRNA expression of antioxidation and autophagy-related genes to reduce Aβ deposition and neurotoxicity to prolong the lifespan of *Caenorhabditis elegans* (C.elegans) (Tang et al., 2022). However, the lack of positive controls prevents us from determining whether DISS has an advantage over marketed drugs. Interestingly, it has been reported that the extract of PR can prevent axon growth cone collapse and axon atrophy caused by Aβ deposition by inhibiting endocytosis in transgenic mice and cultured neurons, but it has no effect on Aβ deposition. A study shows that significant clinical benefit can be observed in clinical trials only if the Aβ plaque is reduced to a low level (∼20 centiloids) (Karran and De Strooper, 2022). This finding reminds suggests making full use of advanced technology and equipment to quantify and monitor the magnitude and velocity of Aβ clearance in future experiments to screen the metabolites that are worthy of further clinical trials, which would help transform scientific research results into clinical application achievements.

It is well known that the presence of alpha-synuclein in the cytoplasm of residual neurons is one of the recognized pathological features of PD, and repeated amplification of more than 35 CAG trinucleotides leads to a long mutant polyQ region in the Huntington protein that causes HD. The therapeutic effect of ambroxol in PD is associated with a reduction in alpha-synuclein. It is more effective in combination with PR and is safe while not affecting its absorption (Yang et al., 2021). This evidence shows that PR and its metabolites can inhibit the secretion and production of Aβ, weaken its toxicity, promote its degradation and clearance, inhibit the hyperphosphorylation of Tau protein, and accelerate the clearance of the Huntington protein and α -synuclein to inhibit the deposition of all these proteins.

#### Improving synaptic dysfunction effects

The synapse is the hub of neuronal signal transduction, and its function is influenced by neurotransmitters, calcium, presynaptic vesicle dynamics, and postsynaptic signal transduction. Its functional obstruction is one of the culprits of NDD. The therapeutic and relieving effects of PR on symptoms triggered by NDD are closely related to its effects of preventing axonal degeneration and improving synaptic plasticity. M2 microglia can inhibit inflammation and show a neuroprotective effect, and their relative preponderance over M1 can be induced by PR to protect the injured spinal cord from axonal degeneration. Sibiricose A5, DISS, PX3, sibiricose A6, and sibiricaxanthone A were detected in the spinal cord extract, and sibiricose A5 and DISS were found to be the active ingredients (Kuboyama et al., 2021). RP01-1 is a pectin from PR that showed a neurite outgrowth-inducing effect by increasing brain-derived neurotrophic factor (BDNF) expression and promoting AKT, ERK, and cAMP-response element-binding protein (CREB) and phosphorylation (Zeng et al., 2020b). Oligosaccharide esters (OEs) of PR can also promote BDNF, pAkt/Akt, and pCREB/CREB expressions (Niu et al., 2022). The dendritic length and dreblin cluster density were quantified by an *in vitro* high connotation imaging analysis system and immunocytochemical analysis, and the results showed that PR and tenuifolin could reduce dreblin cluster density by N-methyl-D-aspartic acid-type glutamate receptors, directly improving synaptic plasticity, and PR had a better effect than tenuifolin alone (Koganezawa et al., 2021). The synergistic effects of complex metabolites in PR and other monomers with this effect need further exploration and screening.

#### Maintaining protein steady-state effects

Due to the close polarity and mutual transformation of saponins, it is difficult to separate and purify the effective metabolites, and fully clarifying the synergistic effect of each metabolite in the extract and the influence of other metabolites on the pharmacokinetics of onjisaponin B. requires further work. Tenuifolin (TEN) can remove damaged mitochondria by up-regulating the mRNA expression of LC3, cathepsin D, Rab7, PTEN-induced putative kinase 1 (PINK1), Parkin, and the protein expression of LC3, PINK1, Parkin, and down-regulating the expression of p62 *in vivo* (Lu et al., 2021). When it was used to treat PC12 cells induced by Aβ25-35, it upregulated p62 expression (Li et al., 2023a). This divergence needs to be further verified and explained. A 2022 study found that TEN could remove damaged mitochondria via activating the mitochondrion phagocytosis mediated by PINK1/Parkin as well. It induced the formation of mitochondrial phagocytes and mitochondrial lysosomes, promoted the transformation from LC3 I to LC3 II, and downregulated p62 expression, and the transfer of Parkin to mitochondria was also promoted (Tian et al., 2022). In addition, PR can also inhibit autophagy protection-related proteins to treat spinal cord injury. TEN upregulated growth-associated protein 43 and neurofilament 200, decreased the Beclin-1 and LC3B-II/LC3B-I levels in the spinal cord, and suppressed the level of the PTPN1 protein, which can suppress IRS1 protein to reduce the pAkt and mTOR levels. What is more, the promoting effect of TEN on the functional recovery from spinal cord injury (SCI) rats was blocked by overexpression of PTPN1, an Akt/mTOR antagonist LY294002, and an autophagy inhibitor 3-MA. This indicates that TEN inhibits autophagy by blocking PTPN1 and modulating IRS1/Akt/mTOR signal transduction to promote the functional recovery of SCI rats (Zang et al., 2023).

#### Regulating energy metabolism effects

Energy metabolism defects are involved in the pathological process of NDD, and maintaining the steady state of energy metabolism is a necessary condition for the normal function of neurons. Mitochondria, the basic organelles of almost all cells in the human body, supply energy, synthesize reactive oxygen species, maintain calcium homeostasis, and affect cell death. In addition to improving glucose utilization, PR can also regulate mitochondrial dysfunction to improve brain energy metabolism and play an important role in treating NDD. It can reverse the phenomena of decreased numbers of mitochondria, structural swelling, vacuole degeneration, distortion or disappearance of the mitochondrial crest, decreased mitochondrial membrane potential (MMP), and enhanced Cyt c expression, apoptosis, and oxidative stress. What is more, GSH, superoxide dismutase (SOD), catalase (CAT), and glutathione peroxidase (GSH-Px) expression were increased, caspase-3 expression was downregulated, and tyrosine hydroxylase expression was upregulated. Tenuifolin has a mitochondrial protective function as well, which has been verified in many *in vitro* and *in vivo* studies. It can prevent the loss of MMP, reduce the positive rate of β-galactosidase, increase intracellular ATPase activity, inhibit the increase of inflammatory factors, downregulate the expression of NF-κB and cyclooxygenase-2 (COX-2), upregulate the expression of PPARγ and PGC-1α, and inhibit the activation of caspase-3 and caspase-9 (Wang et al., 2019; Jin et al., 2022; Li et al., 2023b). In recent years, significant changes in lipid metabolism have been observed in the brains of AD models, which may be a new target of NDD (Zhang et al., 2021), but it is not clear whether PR has an effect on this target. In order to further reveal the active mechanism of PR, laser microdissection, in combination with high-performance liquid chromatography-mass spectrometry, can be used to probe the probable lipid changes in the micro area in the brains of AD models before and after treatment.

#### Antioxidative stress effects

PR has played an anti-AD role in mouse and PC 12 cell models induced by Aβ1-42, which improved the cognitive impairment of mice, increased cell viability, and inhibited apoptosis. A mechanism study showed that PR reduced the contents of reactive oxygen species (ROS) 8-hydroxy-deoxy-guanosine (8-OHdG) and 3-nitrotyrosine (3-NT), activated the PI3K/Akt signaling pathway, and increased the ratios of P-PI3K/PI3K, pAkt/Akt and Bax, enhanced heme oxygenase 1 (HO-1), and promoted nuclear factor E2-related factor 2 (Nrf2) nuclear translocation (Ren et al., 2022). The protective effect of onjisaponin B on a 1-methyl-4-phenyl-1.2.3.6-tetrahydropyridine (MPTP)-induced PD mouse model is achieved through the antioxidant and anti-inflammatory activities mediated by the RhoA/ROCK2 signaling pathway (Peng et al., 2020b). Tenuifolin can prevent the upstream activating factor of NF-κB and the migration of NF-κB to the nucleus and inhibit the release of inflammatory factors and the expression of inducible nitric oxide synthase (iNOS) and COX-2, thus antagonizing nitric oxide (NO)-induced oxidative stress and protecting BV2 and SH-SY5Y cells from Aβ42-induced inflammatory and oxidative stress (Chen and Jia, 2020). It can also improve the behavioral performance of sleep-disordered rats induced by sleep deprivation in a Y maze, object recognition, and an avoidance test, stimulate the production of anti-inflammatory factor IL-10, reduce the production of pro-inflammatory factors IL-1β, IL-6, and IL-18, and activate microglia and increase the expression of 2-related factor 2 and heme oxygenase-1 (HMOX1). At the same time, the expressions of NLRP 3 and caspase-1 p20 were inhibited, the signal cascade of BDNF was downregulated, and the damage to the hippocampal nerve was alleviated (Jiang et al., 2023).

#### Anti-central cholinergic system dysfunction effect

The binding of tenuifolin and onjisaponin B with ChEs was studied using various spectral techniques (Gao and Du, 2022). It was found that there was mainly hydrophobic force between tenuifolin and acetylcholinesterase (AChE), which did not affect its structure, quenched its fluorescence dynamically by affecting tryptophan, and had no inhibitory activity on ChEs. The inhibitory effect of tenuifolin on AChE in these two experiments is the opposite, which may be due to the difference between the internal and external environments. More experimental evidence is needed. Onjisaponin B mainly binds ChEs by van der Waals forces and hydrogen bonds and changes their structures, which has cholinesterase (ChE) inhibitory activity by influencing the static quenching of endogenous fluorescence of tyrosine residues. Similarly, due to the difference in the internal and external environments, the enhancement potential of onjisaponin B on the central cholinergic system needs to be verified in more disease models. The aerial part of PR improved learning and memory impairment induced by D-galactose/NaNO_2_ and scopolamine in mice, increased the levels of ACh, choline acetyl-transferase (ChAT), BDNF, IL-10, glutathione, and SOD, and decreased the levels of IL-1β, AChE, and malondialdehyde (MDA) (Wang et al., 2020a). In addition, it also increased the protein and mRNA expressions of BDNF and tropomyosin receptor kinase B in the hippocampus (Zhang et al., 2020). These inspiring results show that scholars should compare the pharmacological effects and mechanisms of the aerial part of PR with its root. Expanding its application scope would be beneficial to reducing waste.

#### Anti-neuroinflammatory effects

Seventeen saponins, including onjisaponin B, can induce mitosis and inhibit NOD-like receptor thermal protein domain associated protein 3 (NLRP3) inflammatory corpuscles by up-regulating SHP-2 to activate AMPK/mTOR and PINK1/parkin signaling pathways in Aβ1–42-, A53t-α-synuclein-, or Q74-induced BV-2 cells (Qiu et al., 2022). TEN can scavenge free radicals, downregulate NO, ROS, various inflammatory factors [TNF-α, IL-1β, IL-6, COX-2, prostaglandin E2(PGE2)], matrix metalloproteinases (MMP-9 and MMP-2) and other cytotoxic factors, and upregulate transcription factors related to oxidative stress (Nrf2 and HO-1) (Pi et al., 2020). The regulation of inflammatory and oxidative stress-related transcription factors is also one of the mechanisms by which tenuifolin improves the dyskinesia of the demyelinating mouse model induced by cuprizone (CPZ), increasing the myelin content in the corpus callosum, thus alleviating demyelination and treating multiple sclerosis (Li et al., 2023c). Through a combination of network pharmacological prediction and *in vitro* and *in vivo* experiments, it was found that the anti-inflammatory and anti-apoptosis effects of PA may be realized through PPARγ/NF-κB pathway because its effects can be reversed by PPARγ inhibitor GW9662 (Zhao and Jia, 2024). The development of network pharmacology and molecular docking technology makes elucidating the mechanism of action of drugs convenient. With this help, the complex pathways and targets of PR will become clear. Another polysaccharide from PR, PTP70-2, reduces ROS overproduction and MMP dissipation, which may be related to its downregulation of MyD88 and NF-κB signals through the toll-like receptor 4 (TLR4) pathway. Co-incubation with TLR4 inhibitor TAK242 also supports this possibility (Chen et al., 2022a). In addition, the heteropolysaccharide PTBP-1-3 isolated from the crude polysaccharide alkali extract of PR, which can inhibit the activation of microglia/astrocytes induced by lipopolysaccharide (LPS), reduces the production of NO, TNF-α, and IL-1β in a manner similar to minocycline (Zeng et al., 2022). The effective mechanisms of these new polysaccharides need to be further revealed and clarified, laying the foundation for them to be lead metabolites.

#### Neuron protection and regeneration effects

One study showed the effect of DISS was better than onjisaponin B and tenuifolin (Wang et al., 2021b). Bioinformatics methods and verification and verification based on *in vitro* experiments revealed that DISS can also prevent intracellular calcium overload and abnormal calpain system, increase synaptic protein expression, reduce m-calpain expression, inhibit oxidative stress and iron death, and upregulate BDNF/tropomyosin-related kinase B signal transduction with the help of the and d-galactose combined with Aβ1—42 (GCA) in an induced AD model (Li et al., 2023d).

#### Other effects

In addition to the mechanisms mentioned above, PR can also prevent and treat NDD by exerting estrogen-like effects that regulate the intestinal microenvironment and the immune system. PR reversed these phenomena in a mouse model of estrogen exhaustion induced by 4-vinylcyclohexene diepoxide and increased the expression of Bcl-2-associated athanogene (BAG1) in the hippocampus without increasing the serum estradiol level (Han et al., 2021). The methanol metabolite of PR containing 17 saponins can reverse the abnormal increase of C3 complement protein, increase beneficial bacteria in the intestine, inhibit harmful bacteria to regulate the intestinal microenvironment, improve spatial memory of mice, and weaken neuroinflammation and aging and improve the behavior of *C. elegans* and prolong its life (Zeng et al., 2021b).

### Anti-cardiovascular effects

Modern research shows that TEN can significantly improve neurological function and reduce brain edema, hematoma volume, and hemoglobin level in an intracerebral hemorrhage (ICH) model 72 h after administration with the optimal dosage of 16 mg/kg by regulating 26 differentially expressed proteins mainly related to the complement and coagulation cascade (Wang et al., 2024). Both the root and aerial parts of PR can protect against myocardial injury induced by isoproterenol in rats by inhibiting oxidative stress. There was no significant difference between them, which once again proves that the aerial parts of PR have medicinal value (Fu et al., 2022). TEN has protective effects on brain diseases caused by hypoxia/reoxygenation and ischemia/reperfusion, and its mechanism is related to neuroprotection, inhibition of lipid peroxidation, apoptosis, and iron death. In the H/R model, it can increase the cell survival rate, enhance the expression of JNK and -c-Jun proteins, inhibit their phosphorylation, upregulate Bcl-2, SOD, GSH, GSH-Px, GPX4, SLC7A11 and MMP, inhibit Bax, caspase-3, NOX, LDH, MDA, ACSL4, and decrease intracellular ROS accumulation and Ca^2+^, Fe^2+^ levels (Liao et al., 2019; Qiu et al., 2021). Additionally, TEN could cooperate with endothelial progenitor cell transplantation to improve cardiac function in rats with acute myocardial infarction (Jin and Qiao, 2019). Generally speaking, PR has a strong therapeutic effect on cardiovascular and cerebrovascular diseases by regulating calcium channels, inhibiting cell death, regulating mitochondrial function, inhibiting oxidative stress, and inhibiting inflammation. TEN is its main effective metabolite.

### Anti-mental disorder effects

Under the influence of COVID-19, in 2020, the number of patients with major depression and anxiety increased by 28% and 26%, respectively (Collaborators, 2021). According to the WHO World Mental Health Report 2022, nearly 1 billion people in the world suffer from mental illness. Statistics show that by December 2023, there were 6.988 million registered patients with severe mental disorders in China. Mental illnesses, such as depression, anxiety disorder, schizophrenia, bipolar disorder, and post-traumatic stress disorder, can not only lead to disability and suicide but can also lead to a significant reduction in life expectancy (Chan et al., 2023). Scholars are looking for and developing drugs to prevent and treat these diseases that bring heavy economic burdens and social problems to individuals, countries, and even the world. PR has attracted much attention because of its anti-mental disorder effect, and the related effects and mechanisms are summarized as follows.

#### Antidepressant effects

Depression is the most common psychological disorder with high incidence and high mortality, and patients often have significant and persistent depression and pessimism. In addition to the effects of genetics and adverse life circumstances, the pathogenesis of depression involves multiple abnormalities of the monoamine neurotransmitter hypothalamic-pituitary-adrenal (HPA) axis, inflammatory cytokines, neuroplasticity and neurogenesis, and brain structure and function. Depression is triggered when there is a decrease in the level or function of neurotransmitters (e.g., 5-hydroxytryptamine (5-HT), DA, NE) and ACh in the synaptic gaps of the CNS that can affect the body’s sense of pleasure, wellbeing, transmission of emotions, and maintenance of learning and memory abilities. PR can inhibit the decreased levels or function of these neurotransmitters, exerting antidepressant effects similar to those of monoamine or triamine reuptake inhibitors. The effects of these two metabolites on animal models are unknown. In addition, polygalatenoside A compounds extracted from other plants have been reported to have antimelanoma potential (Kim et al., 2022). Wang et al. found that the antidepressant mechanism of tenuifolin was related to increasing the expression of 5-HT and decreasing the expression of indoleamine 2; 3-dioxygenase (IDO) in the cerebral cortex and inhibiting the AChE activity and increasing the activity of ChAT in the hippocampus of mice (Wang, 2020). PR can reverse abnormal behavior and enhance expression of LC3-II and beclin1 in chronic restraint stress (CRS) rats in the sucrose preference test, novelty inhibition feeding test, open-air test, and forced-swim test (FST), and it also reduces P62 levels in both in the cortex of mice and prefrontal cortex of rats and reverses microglia activation, astrocyte damage, NLRP3, ASC, caspase-1 protein and pro-inflammatory cytokine expression increases (Zhou et al., 2021).

The antidepressant effect of PR is also related to regulating intestinal flora, neuroprotection, and affecting metabolism *in vivo*. An extract and oligosaccharide ester of PR improved the depressive behavior of rats with chronic unpredictable mild stress (CUMS), increased the levels of NE, 5-HT, dihydroxyphenylacetic acid (DOPAC), and 5-hydroxyindoleacetic acid (5-HIAA) in the hippocampus, and decreased the levels of CRF, adrenocorticotropic hormone, corticosterone, IL-6, and LPS in serum. The results of transmission electron microscope observation show that PR can improve the structure of intestinal flora, restore the function of the intestinal barrier, reduce the release of intestinal endotoxin, and reduce the level of inflammation, thus playing an antidepressant role (Chen et al., 2021; Chen et al., 2023). Another study showed that the extract of PR could inhibit the activation of NF-κB-NLRP3 signal and intestinal inflammation, increase probiotics such as *Lactobacillus* and *Bacteroides*, regulate the content of metabolites and enzyme expression related to the metabolism of tryptophan-kynurenine in the colon, and increase the expression of tight junction protein to balance the gastrointestinal environment (Li et al., 2022a). However, it is necessary to screen the effective monomer metabolites in the extract and further explore whether the metabolites exert synergistic effects by influencing each other’s structures or generating co-assemblies. Yunfeng Zhou first discovered the disorder of metabolic pathways such as amino acids, energy, intestinal flora, and purine in the urine and hippocampus of olfactory bulbectomy rats by non-targeted metabonomics. This study confirmed that PR could enhance autophagy, correct the abnormality of the AMPK-mTOR signal pathway, inhibit neuroinflammation, improve neuroplasticity, play an antidepressant role, and improve the depressive behavior in a despair model, chronic unpredictable stress model, and an olfactory bulb resection model (Zhou, 2020). In a word, PR acts on depression through multiple channels and multiple targets, and it has great potential to be developed as an antidepressant.

#### Sedative, hypnotic, antiepileptic, and anxiolytic effects

Statistics from the WHO show that 27% of people in the world suffer from sleep disorders. Sleep disorder is not only an important pathogenic factor of NDD and mental diseases but also a complication. PR has sedative, hypnotic, antiepileptic, and anxiolytic effects and can be used to improve sleep disorders. PR plays a sedative role in zebrafish by inhibiting 5-HT reuptake, activating GABA, and improving the expression level of GABA transporter 1 (Chen et al., 2020). In the senile insomnia rat model, PR improved the animals’ weight, memory, and sleep time, increased the levels of 5-HT water and GABA, decreased Glu level, and downregulated Fuom and Pcp2, which indicated that the effects of *Polygala tenuifolia* on sleep disorders might be related to nerve and metabolic pathways in addition to the GABA signaling pathway (Ren et al., 2020). PR has been proven to regulate the disorder of circadian rhythm by regulating the CaMKII pathway and treating disorders caused by sleep delay syndrome, extreme nighttime activities, and social jet lag (Haraguchi et al., 2022). Zhang et al. (2023) used the selective antagonist of the GABA benzodiazepine (BZ) site and confirmed that TEN promoted a kind of non-rapid eye movement (NREM) sleep equivalent to physiological NREM sleep through the interaction with GABAA receptor and also confirmed the improvement of sleep disorder of PD mice by TEN. This suggests that animal models with two or more diseases could be used to explore the role of *Polygala tenuifolia*, which is conducive to further understanding its mechanism of treating different diseases with the same treatment. Recently, a study showed that two saponins of PR, YZ-I and YZ-II, had sedative and hypnotic effects, increased the concentration of 5-HT, NE, PGD2, IL-1β, and TNF-α, and inhibited its inflammation to GABAARα2, GABAARα3, GAD65/67, 5-HT1A, and 5-HT2A, as well as DPR, PGD2, iNOS, and TNF-α (Hao et al., 2024). Moreover, the purer YZ-II has a stronger effect on inflammatory factors and inflammatory mediators in a concentration-dependent manner, which suggests that it is necessary to further explore the differences in composition and mechanism of action between the two metabolites and that monomer metabolites also have important research value.

#### Anti-traumatic stress disorder effects

Note that PR plays a major role in Ren Shen Yang Rong Tang, a decoction used to treat several conditions, for improving the low sociality in NPY-KO zebrafish and increasing its time spent in the fish tank area by inhibiting the activity of HPA-, SAM-, and GABAergic neurons and down-regulating CREB signaling (Kawabe et al., 2022). Systematically screening the metabolites of PR that have therapeutic effects on social disorders, verifying their activities *in vitro* and *in vivo*, and exploring their mechanisms could prove useful in promoting their further development.

### Anti-aging and anti-fatigue effects

A study has shown that polysaccharides of PR can prolong the movement time of mice, increase the glycogen storage concentration in the liver and muscle of mice, enhance the activity of lactate dehydrogenase *in vivo*, and inhibit the production of serum urea nitrogen in tired mice, and these effects will be enhanced with the increase in dose (Xie, 2021). However, further studies on the related mechanisms and pathways are needed to reveal the connotation of polysaccharides in PR against exercise fatigue. In the weight-loaded swimming test, hypoxia tolerance under normal pressure, sodium nitrite poisoning survival test, and swimming with load and fatigue rotating rod test, the extracts of PR decreased serum CK, blood urea nitrogen (BUN) and LA, cleared sports metabolites, increased the levels of SOD, GSH, ATP, succinate dehydrogenase (SDH), and malate dehydrogenase (MDH), decreased MDA, and increased the protein expression levels of AMPK and Nrf-2, thus effectively relieving the fatigue and hypoxia of normal mice and hydrocortisone-induced kidney-yang deficiency mice (Wang, 2020c). However, the active monomers contained in PR need to be systematically screened. Overall, PR has the potential to be developed into food additives and health products because of its anti-aging and anti-fatigue effects.

### Anti-tumor effects

Research has revealed that PR is composed of Ara, Gal, and Glc (molar ratio: 2.6: 1.8: 1.0) and has α and β configurations. It can induce apoptosis of lung cancer cells, upregulate the cascade reactions of FAS, FAS-L, and FADD to express the caspase family, induce apoptosis, upregulate LC 3B-II, and downregulate P62 to induce autophagy (Yu et al., 2020). In S180 sarcoma cells and S180 tumor-bearing mice, it increased the ratio of Bax and Bcl-2, promoted cytochrome c release and caspase-9/-3 expression, revised the immune organ indexes, the activities of NK cells and lymphocytes, and increased secretion of IL-2, interferon-γ (IFN-γ) and TNF-α (Yu et al., 2021). The RP02-1 also can inhibit the growth of pancreatic cancer cells, regulate Bcl-2 and Bax, and cleave caspase-3 to inhibit the proliferation, migration, and colony formation of cancer cells *in vivo* and *in vitro* with no toxicity (Bian et al., 2020). However, the safety of these polysaccharides needs further evaluation. In addition, targeted metabonomics technology can be used to study the pharmacokinetics of polysaccharides in target organs, revealing their absorption mechanisms and structure-activity relationships, which is helpful in guiding their structural modification.

Song et al. (2019) found the complex euxanthone with Cu (Ⅱ) has an inhibitory effect on esophagus cancer cells (ECA109), stomach cancer cells (SGC7901), and cervical cancer cells (HeLa) by intercalating and damaging their DNA to inhibit their growth. A study in 2021 used virtual screening, molecular docking, and molecular dynamics simulation to screen and verify that PX3 can inhibit an X-linked inhibitor of apoptosis protein and promote the release of caspases to induce apoptosis, thus treating cancer (Opo et al., 2021). Moreover, 4-O-benzoyl-3′-O-(O-methylsinapoyl)-sucrose from PR can inhibit glucuronidase to relieve side effects such as intestinal bleeding and diarrhea caused by anticancer drugs (Kim et al., 2021). However, these studies have only been verified at the *in vitro* level and lack *in vivo* data.

### Anti-pathogenic microorganism effects

PR has an inhibitory effect on severe acute respiratory syndrome coronavirus 2 *in vitro*, with an IC_50_ value of 9.5 g/mL (Ngwe Tun et al., 2022). This shows that it has a potential therapeutic effect on COVID-19. More *in vitro* and *in vivo* experiments are needed to reveal its mechanisms and targets.

### Other effects

A chloroform extract of PR could inhibit the main target of diabetes—protein tyrosine phosphatase 1B (PTP1B), and xanthones, sterols, and fatty acids were speculated as the main active substances (Mei et al., 2021). The seed oil of PR can inhibit total cholesterol (TC) and triglyceride levels in plasma and the liver, reduce IL-6 and TNF-α levels, inhibit the NF-κB signaling pathway, and promote the inactivation of steroid regulatory element-binding protein-1 (SREBP1) and SREBP2 involved in lipogenesis (Xin et al., 2023). Therefore, it can potentially be developed as a dietary supplement for inhibiting metabolic-associated fatty liver disease.

PR can shorten the clotting time of mice, and the activity of ethyl acetate and n-butanol extracts is stronger than that of water extracts. The anticoagulant substances are mainly xanthones and OEs, and oleic acid and linoleic acid play an auxiliary role (Li et al., 2020). However, it is not clear whether PR acts on endogenous, exogenous, or common coagulation pathways, and it is necessary to further study its influence on coagulation factors. Onjisaponin B can target and regulate p65/Cas3 to resist radiation (Wang et al., 2022).

PR also has anti-inflammatory activities *in vitro* and has inhibitory effects on pro-inflammatory cytokines IL-12, p40, IL-6, and TNF-α. What is more, the sugar units at C-3 and/or C-28 of the aglycon in triterpenoid saponins and substitute groups in molecules of phenolic glycosides, especially –OH, –OCH3, and –COOH, were considered to be critical to their anti-inflammatory effect (Vinh et al., 2020). 3-O-(3,4,5-trimethoxy-cinnamoyl), 6′-O-(p-methoxybenzoyl) sucrose ester (TCMB), onjisaponin Fg, and 3,4,5-trimethoxycinnamic acid methyl ester can significantly inhibit NO and PGE2. TCMB, 3,4,5-trimethoxyxanthone, hydrocotoin, and 3,4,5-trimethoxycinnamic acid methyl ester can inhibit PGE2 most significantly. TCMB can decrease the protein levels of iNOS and COX-2 and the mRNA levels of TNF-α, IL-1β, and IL-6. In addition, the results of molecular docking show that it has an affinity for iNOS and COX-2 (Son et al., 2022). Sun et al. (2023) developed an ultra-high performance liquid chromatography-photo-diode array-electrospray ionization-quadrupole-time-of-flight-mass spectrometry-lipoxygenase-fluorescence detector (UPLC-PDA-ESI-Q-TOF-MS-LOX-FLD) method that can rapidly screen small molecular inhibitors of LOX and other enzymes and determined for the first time that tenuifoliside A, sibiricaxanthone B, 7-O-methylmangiferin, polygalaxanthone Ⅲ, onjisaponin B, onjisaponin F, and onjisaponin Z are the active anti-inflammatory metabolites. These experiments brought new anti-inflammatory metabolites of PR into our view, although they have only been verified *in vitro*. More animal experiments and clinical trials are needed to further validate these anti-inflammatory effects and provide a scientific basis for its selection as a natural anti-inflammatory metabolite for clinical treatment. Its structure can be optimized to enhance its targeting and stability, making it better applied to the development of anti-inflammatory drugs.

The extract of PR (1.2 mg/mL), consisting of eight OEs, two xanthones, and two saponins, had good 2,2-diphenyl-1-picrylhydrazyl (DPPH)·and 2,2′-azinobis-(3-ethylbenzothiazoline-6-sulphonate (ABTS+)·scavenging capacity with a scavenging rate over 80%, and the synergistic effect of oligosaccharide esters and xanthones was presumed to be the material basis (Guo et al., 2019). Future scholars will be able to study the effects of different extraction methods and processes on the antioxidant activities of these polysaccharides. The aerial part of PR can improve the learning and memory ability of model mice, reduce the MDA content in serum, heart, brain, liver, and kidney tissues, and increase SOD, CAT, GSH-Px, and total antioxidant capacity in serum and tissues (Zhang et al., 2019).

In summary, PR has broad-spectrum pharmacological activity, and its related pharmacological research has achieved fruitful results, which provides a sufficient scientific basis for its application. Its further development offers many opportunities and challenges. First, the pathogenesis of various diseases, especially nervous system diseases and cancers, is extremely complicated, and the molecular mechanisms and material basis of PR are still not clear. Advanced technologies, such as spatial chemoproteomics and the combination of deep learning and ultra-high resolution confocal laser scanning microscopy, can be used to observe the changes in cell morphology and function of PR, which is helpful to further understand its pharmacological process and find its targets. In addition, Qualcomm quantitative metabolomics can be combined with LC-MS to screen and identify effective monomer metabolites and small molecular metabolites in the extracts and effective parts of PR. Second, in recent years, with the iteration of science and technology and the development of analytical methods, an increasing number of active metabolites have been separated from PR, especially those with anti-inflammatory activity, further revealing the material basis of pharmacological activities of PR. However, the effects of some metabolites are only reflected *in vitro*; in view of the different metabolic environments *in vivo* and *in vitro*, their activities need to be verified *in vivo* in animal models. Third, disease models based on nematodes, cells, fruit flies, rats, and mice are used to study pharmacological activities at present. In the future, rhesus monkeys, domestic dogs, and other animal disease models with genetic relationships and metabolic pathways that are more similar to humans can be used for the pharmacological study of PR, which will be conducive to improving the success rate of clinical transformation. Fourth, more toxicological and clinical experiments should be carried out on some metabolites that have shown good pharmacological activities *in vitro* and *in vivo* to provide a scientific basis for ensuring their safety and effectiveness and promoting their further development and rational application. Finally, the aerial parts of PR, which are always discarded, also have rich metabolites and similar pharmacological effects. Future research can systematically compare the differences in activity and metabolites between these parts and the roots of PR and provide scientific support for their medicinal value.

## Toxicity

Although PR is rich in active ingredients and has systemic pharmacological effects, its toxic and side effects have been reported in ancient and modern literature, which limits its application to some extent. Modern pharmacological studies have found that the toxicity of PR is mainly aimed at the gastrointestinal tract. PR shows irritation to the gastrointestinal tract in rat and mouse models, which can reduce their activities and food intake, affect their hair, inhibit small intestine movement and gastrointestinal emptying, cause gastrointestinal flatulence, stomach volume expansion, intestinal wall thinning, necrosis, mucosal defects, inflammatory cell infiltration, reverse peristalsis, direction, blood vessel congestion, endothelial cell swelling, and other pathological tissue changes and deaths. The results of biochemical indexes showed that PR could inhibit the levels of gastrin (GAS), motilin, substance P (SP), PGE2, Ca2+-ATPase, pepsin activity, and intermittent cells of Cajal (ICC), cause slow waves in gastric electrogram amplitude, and increase the levels of vasoactive intestinal peptide (VIP), somatostatin (SS) and NO (Cui et al., 2021). Fortunately, the toxicity of PR can be reduced or eliminated by compatibility with other herbs or processing to promote the transformation of toxic substances or introduce new substances to counter its irritation to the gastrointestinal tract. For example, the content of magnolol in gastrointestinal fluid increases after the compatibility of PR and *Magnolia officinalis* cortex, which can inhibit the increase of NO content and VIP expression, regulate the metabolism of energy, amino acids, and fat, and reduce its gastrointestinal toxicity (Ma et al., 2019; Ma et al., 2021). Licorice-simmered PR can improve its irritation to zebrafish and rabbits by reducing DISS and PA, inhibiting the increase of inflammatory factors, and relieving the congestion and swelling of rabbit eyes as well as the damage of iris, cornea, and conjunctiva caused by raw PR (Chen et al., 2024). The aerial parts of PR have no acute toxicity (Sun et al., 2020). The existing toxicological research on PR and its active metabolites is not enough to completely prove its safety. It is necessary to carry out a series of systematic toxicological experiments, including but not limited to skin irritation experiments, mutagenic experiments, and teratogenic experiments.

## Industrial applications

PR is one of the medicinal and edible species approved by the Chinese government for use as a raw material in health foods. More than 70 health products contain PR or its extracts in the Yaozhi Data, most claiming to improve sleep quality. Other products purport to increase bone density, enhance immunity, delay aging, combat fatigue, clear throat, and improve memory. Yue. et al. developed a metabolite beverage with flavedo and polysaccharides of PR that has a good effect on exercise-induced fatigue (Yue et al., 2020). PR is also utilized for medicated diets with high nutritional value, such as Yuanzhi Lianfen Zhou, which is made of PR, polished japonica rice, and lotus seed. It has efficacy in nourishing the heart, improving intelligence, hearing, and eyesight, and relieving amnesia and insomnia.

According to Gaide Chemical Network statistics, *Polygala tenuifolia* extract and *Polygala tenuifolia* root extract are used as skin conditioners in about 207 and 394 cosmetics, respectively, with top safety and no acne risk. These products are various, including liquid foundation, toning lotion, cream, essence, hair tonic, cleansers, shampoo, and foot bath powder. For example, *Polygala tenuifolia* root extract acts as a skin conditioning agent in “The History of Whoo Cheongidan Hwa Hyun Lotion (后天气丹花献滋养乳).”

In addition, PR was made into an antibacterial sleep-aiding bamboo cellulose fiber for home textiles with the effect of helping sleep [CN202211232475.4] (Hu et al., 2023), an atomized liquid with sedative and soothing effects [CN202211087989.5] (Zhu and Liu, 2022), a composition for promoting skin regeneration and improving skin wound surface [CN202180020964.0] (Pu, 2022), a sleep-assisting tea capable of improving sleep [CN 202180020964.0] (Zhao, 2022), pills for improving intelligence and height [CN202210494926.5] (Zhou, 2022), a mask capable of whitening and removing freckles [CN202210505643.6] (Wang, 2022), a composition capable of curing the effects of drink [CN202211063577.8] (Zhang et al., 2022), and other products. Interestingly, PR can also be used as an additive in pig feed to increase the food intake and body weight of growing-finishing pigs [CN202210343455.8] (Zhan et al., 2022).

To sum up, PR has important practical and economic value and can be used to produce many products. We offer the following suggestions to maximize PR applications: First, with the awakening of public awareness of healthcare, a medicated diet shows great potential. Future research could mine medicinal diet data containing Yuanzhi from ancient and modern literature, explore effects and elucidate mechanisms via *in vivo*, *in vitro*, and clinical experiments, summarize differentiated effects across populations, and establish a unified quality control system. These efforts would solidify the foundations for industrialized mass production and market launch. In the second place, the application of PR in cosmetics is still in a marginalized position at present, and most products only utilize the cleaning effect of saponins; future development could address its antioxidant, anti-aging, and anti-inflammation effects and increase its use in products such as maintaining skin barrier stability and improving complexion. The nude mouse model can be used to investigate the effects of these metabolites on skin conditions. Next, effective metabolites and plant additives can be extracted from the aerial and other nonmedicinal parts of PR, reducing waste. Last but not least, the safety of the active ingredients and effective parts of PR should be investigated through the evaluation of the tolerance, toxicological safety, metabolism, and residue of the target animals, which can promote its application in the form of additives in health food, cosmetics, and food.

## Processing research

After processing, the chemical composition and structure and the properties of TCM have changed, and the toxicity is suppressed, which is more in line with the needs of TCM to adapt to the time, local conditions, and people. As a traditional Chinese medicine with superior efficacy and obvious toxic side effects, PR should be processed before clinical application. The ancient processing methods of PR include purification processing methods of removing the wood heart, pounding or grinding into powder, plain stir-frying methods of stir-frying slightly, stir-frying to yellow, baking, and stir-frying to scorch, as well as stir-frying with many different adjuvants (Gao et al., 2020). According to many ancient books, licorice, ginger juice, wine, wheat, black bean juice, bile, rice-washed water, honey, and other auxiliary materials are often used in PR processing. Ancient processing methods of PR are listed in Supplementary Table 3. In addition, there are records of processing PR with two or three of the above adjuvants; the method of decocting PR with black beans and licorice together was recorded in “Jing Yue’s Complete Work” (景岳全书). The common processed products of PR in modern times mainly include stir-fried PR, deep-fried PR, stewed PR, PR stir-fried with licorice juice, *Magnolia officinalis* cortex juice or honey stir-fried PR and, cinnabar mixed PR (Wu et al., 2024). Among them, PR stir-fried with *Magnolia officinalis* cortex juice and stewed PR are not included in the processing standards of Chinese herbal pieces, and few studies have examined PR mixed with cinnabar. The modern PR processing methods are shown in Supplementary Table 4.

### Removing the wood heart

Modern scholars differ on whether to remove the xylem of PR. On the one hand, they think that although the content of effective metabolites is low in xylem, it also has medicinal value (Liu et al., 2012) and should not be removed. On the other hand, they think that the xylem should be removed because the concentrations of its effective metabolites are several times lower than the phloem (Yang et al., 2018). The amino acids and small molecular organic acids contained in the xylem act on NMDA receptors by acting on 91 potential targets such as SLC6A1, GRM5, GRIA1, and SLC1A1, which makes people feel depressed or anxious (Lou, 2023). At present, the various Chinese herbal medicine quality standards have no compulsory requirement to remove the xylem, and more experimental data are required to reach a consensus on this dispute.

### Effect of processing on chemical constituents of PR

The increase of extractive substances after PR processing may be due to the introduction of liquiritin, glycyrrhizic acid, glucose, and other metabolites that increase with the addition of adjuvants, which can be used as a quality control index (Zhu et al., 2019; Cui et al., 2020). Saponins and oligosaccharide esters in PR are unstable and can be hydrolyzed during the processing. One is deglycosylated, and its glycosidic bonds in C3 or C28 were broken, and the other has its ester bonds broken, resulting in the generation of the second-level glycosides and (or) glycosides with less toxicity (Gao et al., 2021). In addition, licorice can promote the hydrolysis of some saponins in PR into onjisaponin Z and tenuifolin, and stewing PR with licorice can promote the absorption and distribution of oligosaccharides and increase their bioavailability (He et al., 2023; Xiong et al., 2023). The changes in the material basis after processing have important implications for reducing toxicity and increasing the efficiency of PR (Zhao et al., 2021). However, the potential co-assembly phenomena, regularity, and supramolecular mechanisms between various excipients and chemical metabolites of PR need further study.

### Processing technology research of PR

The *Chinese Pharmacopoeia* and local processing standards do not specify processing parameters of different PR processed products, and adjuvant dosages and terminal points are not uniform. Currently, the terminal points are judged subjectively by color or whether products are sticky to hands. With the development of analytical technology and understanding of effective substances of PR, its technological optimization indexes have evolved from single extraction content to comprehensive indexes. For example, Yuan et al. (2021) optimized the technological parameters of PR stir-frying with licorice or honey based on seven metabolites. Song et al. (2023) optimized the water addition, licorice dosage, and drying process parameters based on the comprehensive score of five metabolites and established the relationship between the color and metabolites of stewed PR, which provided an important reference for determining the terminal point. However, it is necessary to carry out pre-production tests and scale-up verifications on the optimized processing technology to adapt to modern production and form a standardized operation flow for each processing technology of PR.

## Conclusion and perspectives

PR is a natural product with a long medicinal history, rich chemical metabolites, wide pharmacological effects, and high medicinal value. Research has achieved fruitful results and provides a scientific basis for its wide application in medicine, healthcare products, and cosmetics. However, some shortcomings need to be solved in follow-up studies.

As a popular best-selling herb at home and abroad, the scarcity of wild resources and the long artificial planting cycle put PR in short supply. The aerial parts and wood heart of PR, which were often discarded in the past, have been shown to have medicinal potential, which suggests that their chemical constituents and pharmacological effects should be studied and compared with the root bark of *Polygala tenuifolia* to expand the source of PR and rationally utilize resources.

PR is widely used in medicine pairs, composition, compound prescriptions, healthcare products, and cosmetics. However, the compatibility mechanisms of related drug pairs, compositions, and compound prescriptions have not been fully revealed, and the interaction between the chemical metabolites of the prescriptions is still unclear and needs further study. In addition, its active ingredients need to be evaluated for safety in order to expand its applications in cosmetics and health products.

With the awakening of modern health awareness, the related medicated diets recorded in ancient books and documents show great potential. Future research can study the compatibility principle, proportion, and applicable populations and promote the emergence of innovative products.

PR contains more than 320 metabolites, of which saponins, oligosaccharide esters, and xanthones are the main metabolites. The low content of xanthones may be the reason for less research into these compounds, so more advanced technologies could be used to extract, enrich, separate, and purify them, and more attention should be paid to this kind of metabolite. Few studies report on the structure-function relationships of the active metabolites in PR, limiting the understanding of the potential modifications and optimizations of the structure of its lead metabolites and hindering the artificial synthesis of its active metabolites and the development of related drugs and health products.

The pharmacological effects of PR, especially its effects against nervous system diseases, have been widely studied, but the transformation from scientific research results to clinical results is rarely seen. Future research could reveal the monomer composition of its active sites, explore its synergistic effects, and conduct safety inspections and clinical research on its active metabolites.

Limited toxicological studies show that PR has no toxicity, but it causes gastrointestinal irritation, probably due to its saponins. Studying the metabolism of saponins in blood, urine, feces, bile, cerebrospinal fluid, and target organs is helpful in clarifying its toxicological mechanism. Furthermore, strengthening the research and safety analysis of the safe range of PR and its active ingredients is conducive to its development and application in drugs, health products, and cosmetics.

Only a few of the many PR processing methods reported in ancient books have been used and supported by modern research. Comparing the chemical composition and pharmacological effects of different processed products of PR recorded in ancient books is beneficial to the inheritance and innovation and provides a reference for its application. In addition, optimizing the processing technology of PR by selecting pharmacological activity indexes *in vivo* based on different medicinal purposes helps obtain targeted processed products.

To sum up, this study reviews and analyzes the botany, phytochemistry, pharmacological action, toxicity, industrial applications, and processing research of PR and looks forward to its future development, aiming at providing a reference for its sustainable development and rational application.

## References

[B1] Bian Y. Zeng H. Tao H. Huang L. Du Z. Wang J. (2020). A pectin-like polysaccharide from Polygala tenuifolia inhibits pancreatic cancer cell growth *in vitro* and *in vivo* by inducing apoptosis and suppressing autophagy. Int. J. Biol. Macromol. 162, 107–115. 10.1016/j.ijbiomac.2020.06.054 32531363

[B2] Chai S. Yang F. Yu H. Wang Y. (2018). Analysis of chemical constituents of radix Polygalae by UPLC/ESI-Q-TOF MS. Tianjin J. Tradit. Chin. Med. 35 (01), 60–64. 10.11656/j.issn.1672-1519.2018.01.16

[B3] Chan J. K. N. Correll C. U. Wong C. S. M. Chu R. S. T. Fung V. S. C. Wong G. H. S. (2023). Life expectancy and years of potential life lost in people with mental disorders: a systematic review and meta-analysis. EClinicalMedicine 65, 102294. 10.1016/j.eclinm.2023.102294 37965432 PMC10641487

[B4] Chen H. Zhong J. Li J. Zeng Z. Yu Q. Yan C. (2022a). PTP70-2, a novel polysaccharide from Polygala tenuifolia, prevents neuroinflammation and protects neurons by suppressing the TLR4-mediated MyD88/NF-κB signaling pathway. Int. J. Biol. Macromol. 194, 546–555. 10.1016/j.ijbiomac.2021.11.097 34801584

[B5] Chen Q. Jia T. Wu X. Chen X. Wang J. Ba Y. (2023). Polygalae radix oligosaccharide esters may relieve depressive-like behavior in rats with chronic unpredictable mild stress via modulation of gut microbiota. Int. J. Mol. Sci. 24 (18), 13877. 10.3390/ijms241813877 37762181 PMC10530649

[B6] Chen Q. Yu L. Zhao W. Xv R. Chen X. Wu X. (2021). Effect of Polygalae radix extract on gut microbiota of depression rat. Chin. Tradit. Herb. Drugs 52 (08), 2313–2323. 10.7501/j.issn.0253-2670.2021.08.014

[B7] Chen Q. Zhang X. (2019). Triterpenoid constituents and pharmacological activities of Polygalae. Chin. J. Ethnomed Ethnopharm. 28 (19), 49–56. 10.3969/j.issn.1007-8517.2019.19.zgmzmjyyzz201919015

[B8] Chen S. Jia J. (2020). Tenuifolin attenuates amyloid-β42-induced neuroinflammation in microglia through the NF-κB signaling pathway. J. Alzheimers Dis. 76 (1), 195–205. 10.3233/JAD-200077 32444542

[B9] Chen S. Jiang Q. Yang S. Lv B. Ma Z. Li P. (2024). Glycyrrhizae radix et rhizoma processing ameliorates adverse reactions of polygalae radix in zebra fish and rabbit models. J. Ethnopharmacol. 327, 118020. 10.1016/j.jep.2024.118020 38458341

[B10] Chen Y. Tan J. Li J. Liu K. Wang J. Wang Y. (2022b). A new lignanamide from aerial part of Polygala tenuifolia. Chin. Tradit. Pat. Med. 44 (02), 435–438. 10.3969/j.issn.1001-1528.2022.02.018

[B11] Chen Z. Peng C. Pei Z. Zhang M. Yun T. Yang Z. (2020). Effects of tenuifolin on rest/wake behaviour in zebrafish. Exp. Ther. Med. 19 (3), 2326–2334. 10.3892/etm.2020.8476 32104301 PMC7027208

[B12] Cheng M. Li C. Ko H. Ko F. Lin Y. Wu T. (2006). Antidepressant principles of the roots of Polygala tenuifolia. J. Nat. Prod. 69 (9), 1305–1309. 10.1021/np060207r 16989524

[B13] Cheng Y. Tan J. Li J. Wang J. Liu K. Wang S. (2022a). Chemical constituents from the ethyl acetate layer of aerial parts of polygala tenuifolia. J. Chin. Med. Mater 45 (02), 331–334. 10.1080/14786419.2021.2013838

[B14] Cheng Y. Tan J. Li J. Wang S. Liu K. Wang J. (2022b). Chemical constituents from the aerial part of Polygala tenuifolia. Nat. Prod. Res. 36 (21), 5449–5454. 10.1080/14786419.2021.2013838 34903137

[B15] Cho N. Huh J. Yang H. Jeong E. Kim Y. Kim J. (2012). Chemical constituents of Polygala tenuifolia roots and their inhibitory activity on lipopolysaccharide-induced nitric oxide production in BV2 microglia. J. Enzym Inhib. Med. Chem. 27 (1), 1–4. 10.3109/14756366.2011.562203 21740104

[B16] Chou T. Chu J. Mei P. (1947). The sapogenins of the Chinese drug, yüan chih, Polygala tennuifolia, Willd. J. Am. Pharm. Assoc. 36 (8), 241. 10.1002/jps.3030360805 20256463

[B17] Collaborators C. M. D. (2021). Global prevalence and burden of depressive and anxiety disorders in 204 countries and territories in 2020 due to the COVID-19 pandemic. Lancet London, Engl. 398 (10312), 1700–1712. 10.1016/S0140-6736(21)02143-7 PMC8500697 34634250

[B18] Cui Y. Wu P. Zhang D. Zhou Q. Zhen Z. Zhao X. (2020). Comparison of HPLC fingerprints between Polygala tenuifolia and stewed Polygala tenuifolia. J. Chin. Med. Mater. 43 (03), 575–581. 10.13863/j.issn1001-4454.2020.03.012

[B19] Cui Y. Zhao X. Tang Y. Zhang Y. Sun L. Zhang X. (2021). Comparative study on the chemical components and gastrointestinal function on rats of the raw product and Licorice-smmered product of Polygala tenuifolia. Evid-Based Compl Alt. Med. 10.1155/2021/8855536 PMC7810529 33505508

[B20] Deng X. Jin G. (2020). Suggestions for the artificial cultivation and developmentof Polygala radix. J. Shandong For. Sci. Technol. 50 (03), 102–106+176.

[B21] Fan X. He Y. Tian H. Chen X. Ji X. (2020). Polygalae radix industry development in Shanxi province from the perspective of industry chain based on SWOT analysis. Anhui Agr Sci. Bull. 26 (20), 30–33+73. 10.3969/j.issn.1008-0805.2020.10.029

[B23] Feng G. Liu S. Pi Z. Song F. Liu Z. (2019a). Comprehensive characterization of *in vivo* metabolic profile of Polygalae radix based on ultra-high-performance liquid chromatography-tandem mass spectrometry. J. Pharm. Biomed. Anal. 165, 173–181. 10.1016/j.jpba.2018.12.005 30551072

[B25] Fu Q. Sun Y. Shi W. Zheng Z. Wang S. Fang M. (2022). Protective effect of Polygala tenuifolia root and Polygala tenuifolia seedling on myocardial injury in rats. Chin. Tradit. Pat. Med. 44 (12), 4033–4036. 10.3969/j.issn.1001-1528.2022.12.050

[B26] Fujita T. Da-you L. Ueda S. Takeda Y. (1992). Xanthones from polygala tenuifolia. Phytochemistry 31, 3997–4000. 10.1016/s0031-9422(00)97571-x

[B27] Gao C. Du H. (2022). The interaction between two metabolites of polygala tenuifolia and cholinesterases. Protein Pept. Lett. 29 (12), 1051–1060. 10.2174/0929866529666220825143136 36028966

[B28] Gao H. Huang W. Xiong Z. Zhang X. Ye J. Xiong Y. (2020). Research progress on processing of Polygalae radix. Chin. J. Exp. Tradit. Med. Form. 26 (23), 209–218. 10.13422/j.cnki.syfjx.20201558

[B29] Gao H. Xiong X. Zhang Q. Cui J. Ye X. Peng L. (2021). Analysis of composition changes of Polygalae radix before and after processing based on UPLC-LTQOrbittrap. Trad. Chin. Drug Res. Clin. Pharmacol. 32 (12), 1845–1854. 10.19378/j.issn.1003-9783.2021.12.015

[B30] Gao L. Zhou C. Liu Q. Wan X. (2022). Research progress on Polygala saponins and their pharmacological effects. J. Beijing Union Univ. 36 (03), 58–64. 10.27253/d.cnki.gnjzu.2022.000421

[B31] Gao M. (2022). Mechanism of treatment of cognitive disorder in mice with Polygala and Senegenin. Nanjing, China: Nanjing Univ Tradit Chin Med.

[B32] Guo L. Guo R. Qin N. Wang X. Zhang X. (2019). Preparation process,composition and antioxidant activity of the Polygala tenuifolia extract. North Hortic. (13), 121–129. 10.11937/bfyy.20184422

[B34] Han G. Choi J. Cha S. Kim B. Kho H. Jang M. (2021). Effects of radix Polygalae on cognitive decline and depression in estradiol depletion mouse model of menopause. Curr. Issues Mol. Biol. 43 (3), 1669–1684. 10.3390/cimb43030118 34698102 PMC8929121

[B35] Hao K. X. Shen C. Y. Jiang J. G. (2024). Sedative and hypnotic effects of Polygala tenuifolia willd. saponins on insomnia mice and their targets. J. Ethnopharmacol. 323, 117618. 10.1016/j.jep.2023.117618 38141791

[B36] Haraguchi A. Saito K. Tahara Y. Shibata S. (2022). Polygalae Radix shortens the circadian period through activation of the CaMKII pathway. Pharm. Biol. 60 (1), 689–698. 10.1080/13880209.2022.2048863 35298359 PMC8933028

[B37] He M. Zhuang Z. Xing Y. Li H. Zhang X. Zhao X. (2023). Exploring of transformation rule of saponins in Polygalae radix before and after processing based on simulated processing technology. Chin. J. Exp. Tradit. Med. Form. 29 (24), 169–176. 10.13422/j.cnki.syfjx.20230867

[B38] Hu N. Li J. Guo W. Lu Z. Wang Y. Li X. (2023). An antibacterial sleep-aiding bamboo cellulose fiber for home textiles and preparation method thereof.

[B39] Huang Q. Lin L. (2021). Research progress of sedative Chinese medicine Polygala tenuifolia. World J. Sleep. Med. 8 (01), 183–184. 10.3969/j.issn.2095-7130.2021.01.078

[B40] Ikeya Y. Sugama K. Maruno M. (1994). Xanthone C-glycoside and acylated sugar from Polygala tenuifolia. Chem. Pharm. Bull. 42 (11), 2305–2308. 10.1248/cpb.42.2305 7859329

[B41] Ikeya Y. Sugama K. Okada M. Mitsuhashi H. (2008). Four new phenolic glycosides from polygala tenuifolia. Chem. Pharm. Bull. 39 (10), 2600–2605. 10.1248/cpb.39.2600

[B42] Ito H. Taniguchi H. Kita T. Matsuki Y. Tachikawa E. Fujita T. (1977). Xanthones and a cinnamic acid derivatives from Polygala tenuifolia. Phytochemistry 16 (10), 1614–1616. 10.1016/0031-9422(77)84043-0

[B43] Jia Y. Liu L. Yang X. Wang C. Ren H. (2023). A new xanthone from the Polygala tenuifolia Wild of northern Shaanxi. Acta Pharm. Sin. B, 1–13. 10.16438/j.0513-4870.2023-0919

[B44] Jiang H. Liu T. Lin L. Yao Z. Zhao J. (2016). Predicting the potential distribution of Polygala tenuifolia Willd. under climate change in China. Plos One 11 (9), e0163718. 10.1371/journal.pone.0163718 27661983 PMC5035090

[B46] Jiang N. Zhang Y. Yao C. Liu Y. Chen Y. Chen F. (2023). Tenuifolin ameliorates the sleep deprivation-induced cognitive deficits. Phytother. Res. 37 (2), 464–476. 10.1002/ptr.7627 36608695

[B47] Jiang Y. Duan Y. Liu Y. Fang M. Shi R. (2011). Isolation and structure identification of chemical constituent of Yuanzhi(Radix Polygalae). J. Beijing Univ. Tradit. Chin. Med. 34 (02), 122–125.

[B48] Jiang Y. Liu L. Tu P. (2003). Study on chemical constituents of Polygala tenuifolia Ⅲ. Chin. J. Nat. Med. (03), 15–18.

[B49] Jiang Y. Tu P. (2003). Tenuifoliose Q, a new oligosaccharide ester from the root of Polygala tenuifolia Willd. J. Asian Nat. Prod. Res. 5 (4), 279–283. 10.1080/1028602031000111987 14604237

[B50] Jiang Y. Tu P. (2004). Structural characteristics and spectroscopic profiles of xanthones from Polygala. J. Peking. Univ. Heal. Sci(01) 36, 94–98. 10.3321/j.issn:1671-167X.2004.01.022 14970898

[B51] Jiang Y. Tu P. (2005). Four new phenones from the cortexes of Polygala tenuifolia. Chem. Pharm. Bull. 53 (9), 1164–1166. 10.1248/cpb.53.1164 16141588

[B52] Jiang Y. Zhang W. Tu P. Xu X. (2005). Xanthone glycosides from Polygala tenuifolia and their conformational analyses. J. Nat. Prod. 68 (6), 875–879. 10.1021/np050026+ 15974611

[B53] Jin B. Park J. (1993). Studies on the alkaloidal components of Polygala tenuifolia willd. China J. Chin. Mater Med. 18 (11), 675–677. 8003228

[B54] Jin G. Yu H. Lu X. Huang Z. Yang H. (2022). Protective effects of tenuifolin on hippocampus neurons and neuronal mitochondria in APP/PS1 double transgenic mice. Chin. J. Geriatr. Heart Brain Ves Dis. 24 (04), 426–429. 10.3969/j.issn.1009-0126.2022.04.023

[B55] Jin W. Qiao X. (2019). Effect of senegenin on cardiac function in rats with acute myocardial infarction treated by transplantation of EPCs. Chin. J. Gerontol. 39 (02), 413–418. 10.3969/j.issn.1005-9202.2019.02.053

[B56] Karran E. De Strooper B. (2022). The amyloid hypothesis in Alzheimer disease: new insights from new therapeutics. Nat. Rev. Drug Discov. 21 (4), 306–318. 10.1038/s41573-022-00391-w 35177833

[B57] Kawabe M. Nishida T. Horita C. Ikeda A. Takahashi R. Inui A. (2022). Ninjinyoeito improves social behavior disorder in neuropeptide Y deficient zebrafish. Front. Pharmacol. 13, 905711. 10.3389/fphar.2022.905711 36034826 PMC9411948

[B58] Kim H. Hwang E. Park B. Kim S. (2022). Novel potential NOX2 inhibitors, Dudleya brittonii water extract and polygalatenoside A inhibit intracellular ROS generation and growth of melanoma. Biomed. Pharmacother. 150, 112967. 10.1016/j.biopha.2022.112967 35430393

[B59] Kim J. Vinh L. Hur M. Koo S. Park W. Moon Y. (2021). Inhibitory activity of 4-*O*-Benzoyl-3'-*O*-(OMethylsinapoyl) sucrose from *Polygala tenuifolia* on *Escherichia coliβ*-glucuronidase. J. Microbiol. Biotechn 31 (11), 1576–1582. 10.4014/jmb.2108.08004 PMC9705844 34528918

[B60] Koganezawa N. Sekino Y. Kawakami H. Fuchino H. Kawahara N. Shirao T. (2021). NMDA receptor-dependent and -independent effects of natural compounds and crude drugs on synaptic states as revealed by drebrin imaging analysis. Eur. J. Neurosci. 53 (11), 3548–3560. 10.1111/ejn.15231 33851450 PMC8365428

[B61] Kuboyama T. Kominato S. Nagumo M. Tohda C. (2021). Recovery from spinal cord injury via M2 microglial polarization induced by Polygalae Radix. Phytomedicine 82, 153452. 10.1016/j.phymed.2020.153452 33418139

[B62] Kumar H. Song S. More S. Kang S. Kim B. Kim I. (2013). Traditional Korean East Asian medicines and herbal formulations for cognitive impairment. Molecules 18 (12), 14670–14693. 10.3390/molecules181214670 24287997 PMC6270158

[B63] Kuroda M. Shizume T. Mimaki Y. (2014). New acylated triterpene glycosides from the roots of Polygala tenuifolia. Nat. Prod. Commun. 9 (3), 1934578X1400900–382. 10.1177/1934578x1400900326 24689222

[B64] Le T. Jeong J. Kim D. (2012). Clionosterol and ethyl cholestan-22-enol isolated from the rhizome of Polygala tenuifolia inhibit phosphatidylinositol 3-kinase/Akt pathway. Biol. Pharm. Bull. 35 (8), 1379–1383. 10.1248/bpb.b12-00426 22863942

[B65] Lei R. Yang B. Gao J. Wei Y. Hu B. Peng L. (2020). Effects of drought stress on secondary metabolite contents and antioxidant enzyme activities in callus of Polygala tenuifolia Willd. North Hortic. (22), 109–116. 10.11937/bfyy.20194366

[B66] Li C. (2008). Studies on chemical constituents and biological activities of Polygala tenuifolia Willd and Polygala glomerata Lour. Beijing, China: Peking Union Med Coll.

[B67] Li C. Gao F. Qu Y. Zhao P. Wang X. Zhu G. (2023a). Tenuifolin in the prevention of Alzheimer's disease-like phenotypes: investigation of the mechanisms from the perspectives of calpain system, ferroptosis, and apoptosis. Phytother. Res. PTR, 4621–4638. 10.1002/ptr.7930 37364988

[B68] Li C. Yang J. Yu S. Chen N. Xue W. Hu J. (2008a). Triterpenoid saponins with neuroprotective effects from the roots of Polygala tenuifolia. Planta Med. 74 (2), 133–141. 10.1055/s-2008-1034296 18256974

[B69] Li C. Yang J. Yu S. Zhang D. Xue W. Yuan Y. (2011). Triterpenoid saponins and oligosaccharides from the roots of Polygala tenuifolia Willd. Chin. J. Nat. Med. 9 (05), 321–328. 10.3724/SP.J.1009.2011.00321

[B70] Li C. Ye F. Xu C. Chang Q. Liu X. Pan R. (2022a). Effect of radix Polygalae extract on the colonic dysfunction in rats induced by chronic restraint stress. J. Ethnopharmacol. 294, 115349. 10.1016/j.jep.2022.115349 35533914

[B71] Li C. Zhu G. Chen Y. Qu Y. Bian Z. Wang X. (2023b). Protective effect of tenuifolin on HT-22 cells damaged by D-galactose synergistically with Aβ1-42 via PPARγ/PGC-1α signaling pathway. J. Biol. 40 (03), 16–21. 10.3969/j.issn.2095-1736.2023.03.016

[B72] Li J. Jiang Y. Tu P. (2006). New acylated triterpene saponins from Polygala tenuifolia Willd. J. Asian Nat. Prod. Res. 8 (6), 499–503. 10.1080/10286020500173358 16931424

[B73] Li J. Wang D. Kong R. Tian H. Zhang F. Qin X. (2019). Identification of Polygala tenuifolia No.2 by PCR allele-specific primers of rDNA ITS sequence. J. Shanxi Med. Univ. 50 (08), 1143–1148. 10.3969/j.issn.1001-1528.2019.05.044

[B74] Li J. Wu S. Li Y. Yu L. Li Q. (2008b). Determination of trace elements in flos sophorae immaturus and radix polygalae. Lishizhen Med. Mater Med. Res. (02), 332–333. 10.3969/j.issn.1008-0805.2008.02.037

[B75] Li J. Zhang X. Chen T. Wang Q. Du C. Qin X. (2020). Study on the pharmacodynamic material basis of anticoagulant effect of Polygala tenuifolia. J. Shanxi Univ. Chin. Med. 21 (04), 260–263+275. 10.19763/j.cnki.2096-7403.2020.04.08

[B76] Li P. Yan M. Li P. (2005). Isolation and identification of the chemical constituents from Polygala tenuifolia Wild. Chin. J. Med. Chem. (01), 42–45+45. 10.3969/j.issn.1005-0108.2005.01.008

[B77] Li X. Chen S. Chen W. Song J. Zhang Y. (2022b). Research progress on chemical constituents of Polygala tenuifolia and prevention and treatment of Alzheimer′s Disease. J. Chin. Pharm. 57 (01), 15–23. 10.11669/cpj.2022.01.003

[B78] Li Y. Liang X. Zhou Y. (2023c). Effect of Tenuifolin on PINK1/Parkin mediated mitochondrial autophagy in Aβ25-35-induced PC12 cells damage. Liaoning J. Tradit. Chin. Med. 50 (02), 149–152+224-225. 10.3964/j.issn.1000-0593(2023)04-1103-09

[B79] Li Y. Wu H. Liu M. Zhang Z. Ji Y. Xu L. (2024). Polysaccharide from Polygala tenuifolia alleviates cognitive decline in Alzheimer's disease mice by alleviating Aβ damage and targeting the ERK pathway. J. Ethnopharmacol. 321, 117564. 10.1016/j.jep.2023.117564 38081400

[B80] Li Y. Yang Z. Wang H. Yang L. Song L. Xiao B. (2023d). Tenuifolin reduces CPZ-induced demyelination in mice by inhibiting inflammation and oxidative stress. Lishizhen Med. Mater Med. Res. 34 (11), 2599–2605. 10.3969/j.issn.1008-0805.2023.11.09

[B81] Liao D. Zhang L. Lan Y. Wang J. Zhang J. (2019). Protective effect of senegenin on the injury of rat hippocampal neural stem cells induced by oxygen-glucose deprivation/reoxygenation culture *in vitro* . China J. TraditChin Med. Pharm. 34 (07), 2990–2993.

[B82] Ling Y. Li Z. Chen M. Sun Z. Fan M. Huang C. (2013). Analysis of multiple constituents in Cong-Ming-Tang, a Chinese herbal formula for the treatment of amnesia, by high-performance liquid chromatography with quadrupole time-of-flight mass spectrometry. Phytochem. Anal. 24 (6), 677–688. 10.1002/pca.2454 23839964

[B83] Liu J. (2004). Effective constituents of Kai-Xin-San, a basic TCM prescription for a therapy of Alzheimer's Disease. Peking. Union Med. Coll. [Doctoral thesis].

[B84] Liu J. Yang X. He J. Xia M. Xu L. Yang S. (2007). Structure analysis of triterpene saponins in Polygala tenuifolia by electrospray ionization ion trap multiple-stage mass spectrometry. J. Mass Spectrom. 42 (7), 861–873. 10.1002/jms.1210 17554809

[B85] Liu L. Feng W. Liu X. Liang Y. Li C. Wang Z. (2021). Research progress on Polygalae radix. China J. Chin. Mater Med. 46 (22), 5744–5759. 10.19540/j.cnki.cjcmm.20210518.601 34951162

[B86] Liu M. Xu W. Liang N. Fang J. Li Y. (2010). Studiy on chemical constituents of Polygala tenuifolia. Mod. Chin. Med. 12 (09), 18–21. 10.13313/j.issn.1673-4890.2010.09.004

[B87] Liu Y. Li Z. Hu H. Xu S. Chang Q. Liao Y. (2015). Tenuifolin, a secondary saponin from hydrolysates of polygalasaponins, counteracts the neurotoxicity induced by Aβ25-35 peptides *in vitro* and *in vivo* . Pharmacol. Biochem. Be 128, 14–22. 10.1016/j.pbb.2014.11.010 25444865

[B88] Liu Y. Peng D. Yang X. Shi T. Jiang Y. Tu P. (2012). Comparison of the chemical constituents and pharmacological activities between the cortexes and the roots of Polygala tenuifolia. J. Chin. Pharm. 47 (24), 1975–1979.

[B89] Lou Y. (2023). Chemical characterization of aqueous extracts of Polygala tenuifolia and study on the biological effect of "MEN. Taiyuan, China: Shanxi University.

[B90] Lu X. Jin G. Yu H. Yang H. (2021). Study on the effects of Tenuifolin on brain mitochondrial autophagy in AD model mice based on PINKI/Parkin signaling pathway. China Pharm. 32 (22), 2748–2754. 10.6039/j.issn.1001-0408.2021.22.11

[B92] Lv G. (2007). A study of the chemical composition of Polygalae radix. Changchun, China: Changchun Univ of Tradit Chin Med.

[B93] Ma R. Xie Q. Wang J. Huang L. Guo X. Fan Y. (2021). Combination of urine and faeces metabolomics to reveal the intervention mechanism of Polygala tenuifolia compatibility with Magnolia officinalis on gastrointestinal motility disorders. J. Pharm. Pharmacol. 73 (2), 247–262. 10.1093/jpp/rgaa022 33793803

[B94] Ma R. Xie Q. Wang J. Huang L. Tang J. Li W. (2019). Metabolomics study on effects of compatibility of alcohol extracts of Magnolia officinalis and Polygala tenuifolin on urine metabolites in rats. Chin. Pharmacol. Bull. 35 (06), 870–877. 10.3969/j.issn.1001-1978.2019.06.026

[B95] Mei Y. Pu Y. Chen T. Xv X. Zhang X. Zhang F. (2021). Inhibitory effect of Polygala tenuifolia extracts on PTP1B activity. J. Shanxi Univ. Nat. Sci. Ed. 44 (04), 824–831. 10.13451/j.sxu.ns.2019091

[B96] Miyase T. Iwata Y. Ueno A. (1992). Tenuifolioses G-P, oligosaccharide multi-esters from the roots of polygala tenuifolia WILLD. Chem. Pharm. Bull. 40 (10), 2741–2748. 10.1248/cpb.40.2741

[B97] Miyase T. Noguchi H. Chen X. (1999). Sucrose esters and xanthone C-glycosides from the roots of Polygala sibirica. J. Nat. Prod. 62 (7), 993–996. 10.1021/np990084t 10425123

[B98] Miyase T. Ueno A. (1993). Sucrose derivatives from the roots of Polygala tenuifolia. Jpn. J. Pharm. 47 (3), 267–278.

[B99] Ngwe Tun M. Toume K. Luvai E. Nwe K. Mizukami S. Hirayama K. (2022). The discovery of herbal drugs and natural compounds as inhibitors of SARS-CoV-2 infection *in vitro* . J. Nat. Med. 76 (2), 402–409. 10.1007/s11418-021-01596-w 35006524 PMC8743439

[B100] Niu F. Sang X. Yang Y. Tang X. Liu Y. Fang F. (2022). Effects of oligosaccharide esters of polygala tenuifolin on β-Amyloid protein 25∼35 induced SH-SY5Y cell injury and AKT/CREB/BDNF signal pathway. J. Beijing Univ. Tradit. Chin. Med. 45 (04), 414–420. 10.3969/j.issn.1006-2157.2022.04.015

[B101] Opo F. Rahman M. Ahammad F. Ahmed I. Bhuiyan M. Asiri A. (2021). Structure based pharmacophore modeling, virtual screening, molecular docking and ADMET approaches for identification of natural anti-cancer agents targeting XIAP protein. Sci. Rep. 11 (1), 4049. 10.1038/s41598-021-83626-x 33603068 PMC7892887

[B102] Pei J. Wan D. Yang L. (2005). Determining the polysaccharides from Polygala tenuifolia by Phenol-sulphate colorimetry. West China J. Pharm. Sci. (04), 337–339. 10.13375/j.cnki.wcjps.2005.04.023

[B103] Peng F. Lu L. Wei F. Wu D. Wang K. Tang J. (2020a). The onjisaponin B metabolite tenuifolin ameliorates dopaminergic neurodegeneration in a mouse model of Parkinson's disease. Neuroreport 31 (6), 456–465. 10.1097/WNR.0000000000001428 32168102

[B104] Peng L. Yan Y. Chen Y. Shen X. Gao J. Li Y. (2020b). Transcriptome analysis of Polygala tenuifolia seedlings induced by methyl jasmonate and key genes mining for triterpenoid biosynthetic pathway. Chin. Tradit. Herb. Drugs 51 (09), 2517–2529. 10.7501/j.issn.0253-2670.2020.09.029

[B105] Peng Y. Fan L. Mao F. Zhao Y. Xu R. Yin Y. (2018). Genetic diversity and population structure of a protected species: polygala tenuifolia Willd. Cr Biol. 341 (3), 152–159. 10.1016/j.crvi.2018.01.007 29477283

[B106] Pi T. Liang Y. Ou W. Zhu H. Tao Y. Jin X. (2020). Senegenin protects against lipopolysaccharide-induced neurite toxicity in a nerve cell model. Chin. J. Comp. Med. 30 (11), 52–58. 10.3969/j.issn.1671-7856.2020.11.009

[B107] Pu Y. (2022). A composition for topical use including a raw medicine extract containing a combination of longan arillus for skin regeneration and treatment or improvement of skin wounds and its use.

[B108] Qiu W. Q. Ai W. Zhu F. D. Zhang Y. Guo M. S. Law B. Y. (2022). Polygala saponins inhibit NLRP3 inflammasome-mediated neuroinflammation via SHP-2-Mediated mitophagy. Free Radic. Biol. Med. 179, 76–94. 10.1016/j.freeradbiomed.2021.12.263 34933095

[B109] Qiu Z. Zhang H. Li J. Wang H. Lu D. Qi R. (2021). Effects of senegenin on hypoxia/reoxygenation-induced ferroptosis in PC12 cells. Chin. J. Pathophysiol. 37 (06), 988–997. 10.3969/j.issn.1000-4718.2021.06.004

[B110] Ren X. Wang G. Zhang X. Wang Q. Peng Z. (2020). Sedative and hypnotic effects and transcriptome analysis of Polygala tenuifolia in aged insomnia rats. Chin. J. Integr. Med. 26 (6), 434–441. 10.1007/s11655-020-3087-6 32240473

[B111] Ren X. Zhang J. Zhao Y. Sun L. (2022). Senegenin inhibits aβ_1-42_-induced PC12 cells apoptosis and oxidative stress via activation of the PI3K/Akt signaling pathway. Neuropsych Dis. Treat. 18, 513–524. 10.2147/NDT.S346238 PMC8904946 35280979

[B112] Sakuma S. Shoji J. (1981). Studies on the constituents of the root of polygala tenuifolia WILLDENOW. II. On the structures of onjisaponins G and F. Chem. Pharm. Bull. 29 (3), 810–821.

[B113] Sakuma S. Shoji J. (1982). Studies on the constituents of the root of polygala tenuifolia WILLDENOW. II. On the structures of onjisaponins A, B and E. Chem. Pharm. Bull. 30, 810–821. 10.1248/cpb.30.810

[B114] Sang X. Yang Y. Fang F. (2017). Research progress in pharmacological activities of oligosaccharide esters from Polygala tenuifolia. J. Chin. Pharm. 52 (18), 1576–1579. 10.11669/cpj.2017.18.003

[B115] Shen X. Witt M. R. Dekemendjian K. Nielsen M. (2004). Isolation and identification of tetrahydrocolumba-mine as a dopamine receptor ligand from Polygala tenuifolia Willd. J. Hainan Med. Coll. (03), 133–135. 10.3969/j.issn.1007-1237.2004.03.002 7709740

[B116] Shi T. Li Y. Jiang Y. Tu P. (2013a). Isolation of flavonoids from the aerial parts of Polygala tenuifolia Willd.and their antioxidant activities. J. Chin. Pharm. Sci. 22 (01), 36–39. 10.5246/jcps.2013.01.004

[B117] Shi T. Wang S. Zeng K. Tu P. Jiang Y. (2013b). Inhibitory constituents from the aerial parts of Polygala tenuifolia on LPS-induced NO production in BV2 microglia cells. Bioorg Med. Chem. Lett. 23 (21), 5904–5908. 10.1016/j.bmcl.2013.08.085 24042007

[B118] Son S. Yoon Y. Hong J. Kim J. Lee K. Jang D. (2022). Chemical constituents of the roots of Polygala tenuifolia and their anti-inflammatory effects. Plants 11 (23), 3307. 10.3390/plants11233307 36501346 PMC9738712

[B119] Song J. Zhong L. Xue X. Xie Y. Li J. Wang Z. (2023). Optimization of processing technology of stewed Polygala tenuifolia and correlation analysis of its composition and color. Chin. Tradit. Pat. Med. 45 (12), 4085–4090. 10.3969/j.issn.1001-1528.2023.12.039

[B120] Song L. Yu Q. Qi X. Yang A. Zhang X. Shen R. (2019). Antitumor activity of Cu(Ⅱ) complex with bioactive constituent Euxanthone from Polygala. Tianjin J. Tradit. Chin. Med. 36 (07), 701–704. 10.11656/j.issn.1672-1519.2019.07.19

[B121] Song M. Wu P. Zhang X. Li H. Liu J. Meng Y. (2016). Comparison of 8 organic acids in three processed products of Polygalae radix. Chin. Tradit. Pat. Med. 38 (07), 1565–1569. 10.3969/j.issn.1001-1528.2016.07.027

[B122] Song Y. Jiang Y. Bi D. Tian X. Liang L. Tu P. (2012a). Chemical constituents from n-butanol extract of aerial part of Polygala sibirica. China J. Chin. Mater Med. 37 (4), 471–474. 10.4268/cjcmm20120412 22667146

[B123] Song Y. Jiang Y. Zhou S. Bi D. Tu P. (2009). Studies on xanthones from aerial parts of Polygala sibirica. China J. Chin. Mater Med. 34 (5), 574–576. 10.3321/j.issn:1001-5302.2009.05.020 19526786

[B124] Song Y. Zeng K. Shi T. Jiang Y. Tu P. (2013a). Sibiricasaponins A–E, five new triterpenoid saponins from the aerial parts of Polygala sibirica L. Fitoterapia 84, 295–301. 10.1016/j.fitote.2012.12.017 23266727

[B125] Song Y. Zhou G. Zhou S. Jiang Y. Tu P. (2013b). Polygalins D-G, four new flavonol glycosides from the aerial parts of Polygala sibirica L. (Polygalaceae). Nat. Prod. Res. 27 (13), 1220–1227. 10.1080/14786419.2012.724412 22970928

[B126] Song Y. Zhou S. Wei H. Jiang Y. Tu P. (2012b). A novel sterol sulfate and new oligosaccharide polyester from the aerial parts of Polygala sibirica. Nat. Prod. Commun. 7 (9), 1934578X1200700–1168. 10.1177/1934578x1200700914 23074897

[B128] Sun H. (2005). Isolation and evaluation of immunological adjuvant active saponins from Polygala tenuifolia Willd. Hangzhou, China: Zhejiang Univ.

[B129] Sun M. Yuan M. Wang H. Yin R. Yan C. Yin M. (2023). Development of an online UPLC-PDA-ESI-Q-TOF-MS-LOX-FLD system for rapid screening of anti-inflammatory compounds in Polygala tenuifolia Willd. J. Pharm. Biomed. Anal. 229, 115353. 10.1016/j.jpba.2023.115353 36965376

[B130] Sun X. Shi S. Yang G. (2000). Studies on chemical constituents of fat oil of Polygala tenuifolia. J. Chin. Med. Mater 23 (1), 35–37. 10.3321/j.issn:1001-4454.2000.01.017 12575116

[B131] Sun Y. Zhang D. Wang Y. Wang X. Wang L. Li R. (2020). “Experimental study on acute toxicity of aerial part of Polygalae radix,” in Shaanxi toxicology society symposium on prevention and control of epidemic infectious disease and toxic biohazard, Shaanxi and Shanxi. China.

[B132] Takiura K. Honda S. (1964). Sugar components of the root of *Polygala tenuifolia* . Yakugaku zasshi J. Pharm. Soc. Jpn. 84, 1223–1224. 10.1248/yakushi1947.84.12_1223 14266556

[B133] Tan J. Li J. Su Q. Cheng Y. Chai Z. Ma C. (2022). Chemical constituents of n-butanol fraction from aerial part of Polygala tenuifolia. Mod. Chin. Med., 1–5. 10.13313/j.issn.1673-4890.20211221006

[B134] Tang X. Zhao Y. Liu Y. Liu Y. Liu Y. Niu F. (2022). 3,6'-disinapoyl sucrose attenuates Aβ_1-42_ - induced neurotoxicity in *Caenorhabditis elegans* by enhancing antioxidation and regulating autophagy. J. Cell Mol. Med. 26 (4), 1024–1033. 10.1111/jcmm.17153 35044105 PMC8831957

[B135] Teng H. Fang M. Cai X. Hu Z. (2009a). Localization and dynamic change of saponin in vegetative organs of Polygala tenuifolia. J. Integr. Plant Biol. 51 (6), 529–536. 10.1111/j.1744-7909.2009.00830.x 19522811

[B136] Teng H. Fang M. Hu Z. (2009b). The structure of vegetative organs, and saponins histochemical localization and content comparization in Polygala sibirica L. J. Mol. Cell Biol. 42 (1), 61–69. 19306690

[B137] Tian Y. Qi Y. Cai H. Xu M. Zhang Y. (2022). Senegenin alleviates Aβ_1-42_ induced cell damage through triggering mitophagy. J. Ethnopharmacol. 295, 115409. 10.1016/j.jep.2022.115409 35640739

[B138] Tsukada M. Ikemoto H. Lee X. Takaki T. Tsuchiya N. Mizuno K. (2021). Kamikihito, a traditional Japanese Kampo medicine, increases the secretion of oxytocin in rats with acute stress. J. Ethnopharmacol. 276, 114218. 10.1016/j.jep.2021.114218 34029638

[B139] Vinh L. Heo M. Phong N. Ali I. Koh Y. Kim Y. (2020). Bioactive compounds from Polygala tenuifolia and their inhibitory effects on lipopolysaccharide-stimulated pro-inflammatory cytokine production in bone marrow-derived dendritic cells. Plants 9 (9), 1240. 10.3390/plants9091240 32962290 PMC7570142

[B140] Wang B. Qiao P. Wang W. Song W. Liu C. Wang X. (2021a). Effect of Albiziae Flos and Polygalae radix alone and their combination on depression-like behavior and CREB and NOX2 expression in Hippocampus of chronic unpredictable stress-induced rats. Chin. J. Exp. Tradit. Med. Form. 27 (17), 32–39. 10.13422/j.cnki.syfjx.20211604

[B141] Wang H. (2022). A kind of whitening and spot-removing mask and its preparation method.

[B142] Wang H. Tong Y. Ye W. Zhao S. (2003). Studies on chemical constituents from the root of Polygala tenuifolia. China J. Chin. Mater Med. (09), 38–40. 10.3321/j.issn:0253-2670.2002.10.004 15015373

[B143] Wang L. (2020). Studies on the anti-exercise fatigue and hypoxia tolerance effects of Polygala herb extract Master's Theses. Xian, China: Northeastern Univ.

[B144] Wang L. Jin G. Yu H. Lu X. Zou Z. Liang J. (2019). Protective effects of tenuifolin isolated from Polygala tenuifolia Willd roots on neuronal apoptosis and learning and memory deficits in mice with Alzheimer's disease. Food Funct. 10 (11), 7453–7460. 10.1039/c9fo00994a 31664284

[B145] Wang L. Xia J. (2012). Plant resources and traditional curative effects of medicinal Polygala tenuifolia. Heilongjiang Sci. Technol. Inf. (01), 16. 10.3969/j.issn.1673-1328.2012.01.036

[B146] Wang L. Zhu S. Li D. (2020a). Effect of tenuifolin on mental behavior in deprssion model mice. Chin. J. Public Health 36 (04), 570–573. 10.11942/j.issn1002-2767.2020.10.0093

[B147] Wang P. Shen Y. Manaenko A. Liu F. Yang W. Xiao Z. (2024). TMT-based quantitative proteomics reveals the protective mechanism of tenuigenin after experimental intracerebral hemorrhage in mice. J. Ethnopharmacol. 319, 117213. 10.1016/j.jep.2023.117213 37739103

[B148] Wang T. Hu Y. Wang X. Li Y. Zhang F. Yan Y. (2022). Targeting p65 to inhibit Cas3 transcription by Onjisaponin B for radiation damage therapy in p65+/- mice. Phytomedicine 104, 154317. 10.1016/j.phymed.2022.154317 35816993

[B149] Wang X. Liu C. Zhou J. Li M. Zhou Y. Jiang W. (2020b). Research advances in the chemical constituents and pharmacological effects of Polygalae radix(PR),a traditional Chinese medicine,and a predictive analysis of potential PR quality-makers. J. Int. Pharm. Res. 47 (07), 483–495+513. 10.27405/d.cnki.gxbdu.2020.000944

[B150] Wang X. Xiao H. Wu Y. Kong L. Chen J. Yang J. (2021b). Active constituent of Polygala tenuifolia attenuates cognitive deficits by rescuing hippocampal neurogenesis in APP/PS1 transgenic mice. BMC Complement. Med. 21 (1), 267. 10.1186/s12906-021-03437-5 PMC8543956 34696749

[B151] Wang X. Zhang D. Song W. Cai C. Zhou Z. Fu Q. (2020c). Neuroprotective effects of the aerial parts of Polygala tenuifolia Willd extract on scopolamine-induced learning and memory impairments in mice. Biomed. Rep. 13 (5), 37. 10.3892/br.2020.1344 PMC7453304 32874571

[B153] Wang Y. Yang J. Zhang J. Chen J. (2005). Studies on chemical constituents of polygala tenuifolia. Chin. Tradit. Herb. Drugs (09), 15–17. 10.3321/j.issn:0253-2670.2005.09.003

[B155] Wu H. Xv R. Zhang Q. Huang Y. (2024). Research progress on processing of Polygalae radix. Glob. Tradit. Chin. Med. 17 (02), 373–376. 10.3969/j.issn.1674-1749.2024.02.036

[B156] Wu Z. (2010). Analysis of the volatile oil components of Polygala tenuifolia Willd. by GC-MS. J. Anhui Agr Sci. 38 (09), 4562+4574. 10.3969/j.issn.0517-6611.2010.09.055

[B157] Xie F. (2021). Effects of anti-fatigue *in vivo* and anti-oxidant *in vitro* of polysaccharides from Polygala tenuifolia Willd.on exhaustive exercise mice. Sci. Technol. Food Ind. 42 (06), 332–336. 10.13386/j.issn1002-0306.2020050292

[B158] Xin M. Wang H. Wang M. Yang B. Liang S. Xu X. (2023). Attenuating effect of Polygala tenuifolia Willd. seed oil on progression of MAFLD. Front. Pharmacol. 14, 1253715. 10.3389/fphar.2023.1253715 37869756 PMC10588625

[B159] Xiong X. Lu M. Shi L. Zhong L. Ye X. (2023). Effect of Jianchang stewing method on the pharmacokinetics and tissue distribution of 4 components in Polygala tenuifolia in rats. Cent. South Pharm. 21 (05), 1149–1156. 10.7539/j.issn.1672-2981.2023.05.006

[B160] Xu L. Li C. Yang J. Luo Y. Zhang D. (2014). Chemical constituents of Polygala tenuifolia root. J. Chin. Med. Mater. 37 (9), 1594–1596. 10.13863/j.issn1001-4454.2014.09.02 25857159

[B161] Yan D. (2004). Studies on extraction of saponin and quality standard of radix Polygalae (Yuanzhi). Chengdu Univ. Tradit. Chin. Med. 10.7666/d.y623536

[B162] Yang B. Li X. Yu K. Jiang X. Wang L. Li F. (2022a). Sugar easters and xanthones from the roots of Polygala tenuifolia Willd. and their cytoprotective activity. Fitoterapia 161, 105256. 10.1016/j.fitote.2022.105256 35870664

[B163] Yang C. Peng W. Lin L. Tsai T. (2021). Protein unbound pharmacokinetics of ambroxol in the blood and brains of rats and the interaction of ambroxol with Polygala tenuifolia by multiple microdialysis. J. Ethnopharmacol. 269, 113764. 10.1016/j.jep.2020.113764 33383115

[B164] Yang F. Yu H. Chai X. Peng S. Yang J. Wu D. (2018). Illumination on "reserving phloem and discarding xylem" and quality evaluation of radix polygalae by determining oligosaccharide esters, saponins, and xanthones. Molecules 23 (4), 836. 10.3390/molecules23040836 29621185 PMC6017119

[B165] Yang S. Liu Y. Xiu M. Yang X. Yang F. He J. (2022b). Research progress on anti-aging mechanism of saponins of traditional Chinese medicine. Chin. J. Gerontol. 42 (13), 3327–3335. 10.3969/j.issn.1008-0104.2022.03.001

[B166] Yang X. Xu L. Yang S. (2000). Advances in the research of chemistry and pharmacology of xanthones extracted from polygala L. Nat. Prod. Res. Dev. (05), 88–94. 10.16333/j.1001-6880.2000.05.020

[B167] Yoo S. Le T. Jeong J. Kim D. (2014). Poligapolide, a PI3K/Akt inhibitor in immunodeficiency virus type 1 TAT-transduced CHME5 cells, isolated from the rhizome of Polygala tenuifolia. Chem. Pharm. Bull. 62 (5), 467–471. 10.1248/cpb.c13-00958 24789928

[B168] Yu H. Lu X. Jin G. Yang H. (2022). The effect of Tenuigenin on inhibiting neuronal cell cycle activation and apoptosis by regulating Aβ transport. Chin. J. Gerontol. 42 (17), 4310–4314. 10.3969/j.issn.1005-9202.2022.17.046

[B169] Yu S. (2020). Preparation,structure and antitumor activity of polysaccharides from the Polygala tenuifolia Master's Theses. Tianjin Univ. Sci. 10.27359/d.cnki.gtqgu.2020.000345

[B170] Yu S. Dong X. Ji H. Yu J. Liu A. (2021). Antitumor activity and immunomodulation mechanism of a novel polysaccharide extracted from Polygala tenuifolia Willd. evaluated by S180 cells and S180 tumor-bearing mice. Int. J. Biol. Macromol. 192, 546–556. 10.1016/j.ijbiomac.2021.10.025 34648800

[B171] Yu S. Ji H. Dong X. Liu A. Yu J. (2020). FAS/FAS-L-mediated apoptosis and autophagy of SPC-A-1 cells induced by water-soluble polysaccharide from Polygala tenuifolia. Int. J. Biol. Macromol. 150, 449–458. 10.1016/j.ijbiomac.2020.02.010 32027895

[B173] Yu X. Liu G. Zhu B. Hao K. Ling F. Wang G. (2014). *In vitro* immunocompetence of two compounds isolated from Polygala tenuifolia and development of resistance against grass carp reovirus (GCRV) and Dactylogyrus intermedius in respective host. Fish. Shellfish Immun. 41 (2), 541–548. 10.1016/j.fsi.2014.10.004 25450998

[B174] Yuan M. Yin M. Tang Z. Xv H. Chen S. (2021). Processing technology of Glycyrrhiza Polygala tenuifolia and honey-stir-baked Polygala tenuifolia by Box-Behnken response surface methodology. Cent. South Pharm. 19 (07), 1310–1315. 10.7539/j.issn.1672-2981.2021.07.008

[B175] Yue T. Huang D. Deng Y. (2020). Study on preparation and resistant exercise fatigue of compound beverage of Polygala tenuifolia Willd.and Flavedo. Food Sci. Technol. 45 (12), 73–79. 10.13684/j.cnki.spkj.2020.12.011

[B176] Yukinobu I. Kô S. Minoru O. Hiroshi M. (1991). Two xanthones from Polygala tenuifolia. Phytochemistry 30 (6), 2061–2065. 10.1016/0031-9422(91)85067-a

[B177] Zang L. Fu D. Zhang F. Li N. Ma X. (2023). Tenuigenin activates the IRS1/Akt/mTOR signaling by blocking PTPN1 to inhibit autophagy and improve locomotor recovery in spinal cord injury. J. Ethnopharmacol. 317, 116841. 10.1016/j.jep.2023.116841 37355079

[B178] Zeng H. Huang L. Tao H. Zhang Y. Ding K. (2020a). Structural elucidation of a pectin from roots of Polygala tenuifolia and its neuritogenesis inducing activity in PC12 cells. Carbohyd Polym. 236, 116048. 10.1016/j.carbpol.2020.116048 32172862

[B179] Zeng H. Li P. Zhou L. Ding K. (2020b). A novel pectin from Polygala tenuifolia blocks Aβ42 aggregation and production by enhancing insulin-degradation enzyme and neprilysin. Int. J. Biol. Macromol. 161, 35–43. 10.1016/j.ijbiomac.2020.05.212 32473218

[B180] Zeng L. Wang C. Wang S. Chen Y. Huang W. Wang Z. (2021a). Study progress on chemical constituents and biological activities of Polygala genus. For. Environ. Sci. 37 (04), 160–175. 10.3969/j.issn.1006-4427.2021.04.023

[B181] Zeng W. Wu A. Zhou X. Khan I. Zhang R. Lo H. (2021b). Saponins isolated from Radix polygalae extent lifespan by modulating complement C3 and gut microbiota. Pharmacol. Res. 170, 105697. 10.1016/j.phrs.2021.105697 34062240

[B182] Zeng Z. Chang X. Zhang D. Chen H. Zhong X. Xie Y. (2022). Structural elucidation and anti-neuroinflammatory activity of Polygala tenuifolia polysaccharide. Int. J. Biol. Macromol. 219, 1284–1296. 10.1016/j.ijbiomac.2022.08.161 36037912

[B183] Zhan N. Liu X. Ren H. (2022). A herbal feed additive for growing and fattening pigs and its preparation method.

[B184] Zhang D. Miyase T. Kuroyanagi M. Umehara K. Ueno A. (1996). Five new triterpene saponins, polygalasaponins XXVIII-XXXII from the root of Polygala japonica Houtt. Chem. Pharm. Bull. 44 (4), 810–815. 10.1248/cpb.44.810 8681413

[B185] Zhang D. Wang X. Li R. Wang L. Zhou Z. Fu Q. (2020). Extract of the aerial part of Polygala tenuifolia attenuates d-Galactose/NaNO2-induced learning and memory impairment in mice. Planta Med. 86 (18), 1389–1399. 10.1055/a-1212-3212 32797467

[B186] Zhang D. Wang Y. Wang X. Wang L. Li R. Fang M. (2019). Antioxidant effects of aqueous extract from Polygala tenuifolia Willd. seedlings on D-galactose induced aging mice. China J. Tradit. Chin. Med. Pharm. 34 (09), 4322–4326.

[B187] Zhang D. Zhang W. Deng S. Liu L. Wei H. Xue F. (2023). Tenuigenin promotes non-rapid eye movement sleep via the GABA_A_ receptor and exerts somnogenic effect in a MPTP mouse model of Parkinson's disease. Biomed. Pharmacoth 165, 115259. 10.1016/j.biopha.2023.115259 37531785

[B188] Zhang J. Han B. Wang X. Liu Z. (2022). An anti-alcoholic composition and its preparation method and application.

[B189] Zhang T. Rong W. Li Q. Bi K. (2016). Research progress on Polygalae radix. Chin. Tradit. Herb. Drugs 47 (13), 2381–2389. 10.7501/j.issn.0253-2670.2016.13.029

[B190] Zhang X. Wu C. Tan W. (2021). Brain lipid dynamics in amyloid precursor protein/presenilin 1 mouse model of early Alzheimer's disease by desorption electrospray ionization and matrix assisted laser desorption ionization-mass spectrometry imaging techniques. J. Prot. Res. 20 (5), 2643–2650. 10.1021/acs.jproteome.0c01050 33780243

[B191] Zhao T. Jia J. (2024). Polygalacic acid attenuates cognitive impairment by regulating inflammation through PPARγ/NF-κB signaling pathway. CNS Neurosci. Ther. 30 (2), e14581. 10.1111/cns.14581 38421141 PMC10851321

[B192] Zhao X. (2022). A Tea-replacing drink capable of helping sleep.

[B193] Zhao X. Cui Y. Wu P. Zhang Z. Wang Y. Zhang X. (2021). Regulatory effect of Polygala tenuifolia and Licorice-simmered *P. tenuifolia* on learning and memory, HPA axis function and neurotransimitters in rats with heart-kidney lmbalance insomnia. Chin. J. Exp. Tradit. Med. Form. 27 (11), 147–154. 10.3969/j.issn.1001-9677.2021.01.014

[B194] Zhao X. Cui Y. Wu P. Zhao P. Zhou Q. Zhang Z. (2020). Polygalae Radix: a review of its traditional uses, phytochemistry, pharmacology, toxicology, and pharmacokinetics. Fitoterapia 147, 104759. 10.1016/j.fitote.2020.104759 33069838

[B195] Zheng Y. Chen J. You F. Yang H. (2016). Research progress on neuroprotective effect and mechanisms of Polygala tenuifolia. Med. Rec. 22 (08), 1561–1564. 10.3969/j.issn.1006-2084.2016.08.031

[B196] Zhou H. (2022). A pill that has the effect of increasing intelligence and height.

[B197] Zhou H. Zhang L. Zeng J. Wang Z. Liu L. Huang J. (2022). CiteSpace knowledge map of research hotspots and frontiers of Polygalae radix. China J. Chin. Mater Med., 1–12. 10.19540/j.cnki.cjcmm.20221129.501 37005854

[B198] Zhou Y. (2020). Research on the antidepressant effects and mechanisms of radix Polygalae Doctoral Dissertations. Beijing, China: Peking Union Med Coll.

[B199] Zhou Y. Jiang Y. Wen J. Chen Y. Tu P. (2008). Chemical constituents from the roots of Polygala sibirica L. J. Chin. Pharm. Sci. (02), 148–152.

[B200] Zhou Y. Yan M. Pan R. Wang Z. Tao X. Li C. (2021). Radix Polygalae extract exerts antidepressant effects in behavioral despair mice and chronic restraint stress-induced rats probably by promoting autophagy and inhibiting neuroinflammation. J. Ethnopharmacol. 265, 113317. 10.1016/j.jep.2020.113317 32861821

[B201] Zhou Y. Zhang S. Guo Q. Chai X. Jiang Y. Tu P. (2014). Chemical investigation of the roots of Polygala sibirica L. Chin. J. Nat. Med. 12 (03), 225–228. 10.1016/S1875-5364(14)60038-8 24702811

[B202] Zhu J. Liu X. (2022). A atomized liquid with calming and sleep-aiding effect and its preparation method.

[B203] Zhu M. Li L. Yang J. Yang J. Chai X. Wang Y. (2019). Analysis of three components in radix Polygalae and its processed products by the method of differential concentration. J. Tianjin Univ Tradit Chin Med 38 (06), 598–602. 10.11656/j.issn.1673-9043.2019.06.20

