# Corrigendum: A review of the botany, metabolites, pharmacology, toxicity, industrial applications, and processing of Polygalae Radix: the “key medicine for nourishing life”

**DOI:** 10.3389/fphar.2024.1515658

**Published:** 2024-11-01

**Authors:** Hongtuo Kuang, Lingping Kong, Ajiao Hou, Anni Yang, Hai Jiang

**Affiliations:** Key Laboratory of Basic and Application Research of Beiyao, Heilongjiang University of Chinese Medicine, Ministry of Education, Harbin, China

**Keywords:** Polygalae Radix, pharmacological effects, metabolites, applications, toxicity, processing

In the published article, there was an error in the **Acknowledgments** statement. The attribution and license link of two icons used in the GRAPHICAL ABSTRACT was omitted. The correct Acknowledgments statement appears below.

