# Supplementary material for: A review of the botany, metabolites, pharmacology, toxicity, industrial applications, and processing of Polygalae Radix: the “key medicine for nourishing life”
Source: Front Pharmacol. 2024 Sep 18;15:1450733. doi: 10.3389/fphar.2024.1450733 (PMC11445616; doi:10.3389/fphar.2024.1450733)
Supplement: Supplementary file 1 [file Table1.pdf]

Supplementary Table 1 The botanical features of *Polygala tenuifolia* Willd. and *Poly gala sibirica* L.

Feature s	***Polygala tenuifolia* Willd.**	***Polygala sibirica* L.**
Tall	15-50 cm tall	10-30 cm tall
Root	principal root to 10 cm, stout, base fl eshy.	woody
Stem	much branched, caespitose, erect or i nclined, ridged and sulcate, pubesce nt.	caespitose, often erect, pubescen t.
Leaf	subsessile; leaf blade linear to linear -lanceolate, 10-30 × 0.5-1(-3) mm, p aperyglabrous or very sparsely pube rulent, base cuneate, margin entire, r ecurved, apex acuminate	lower leaves ovate, upper leaves lanceolate or elliptic-lanceolate,1 0-20 × 3-6 mm, apex obtuse, bas e cuneate, both surface pubescen t, midvein raised abaxially, impre ssed adaxially, shortly stipitate
Flower	racemes terminal, secund, 5-7 cm, s parsely few flowered; bracts 3, cadu cous; Sepals 5, persistent; outer sepa ls 3, linear-lanceolate; Petals 3, conn ate in lower 1/3, purple; lateral petal s obliquely oblong, inside hairy at ba se; keel longer than lateral petals, ap ex with fimbriate appendages: Filam ents united into a sheath below 3/4, 2 separated in the middle above 3/4, 3 united on each side	racemes extra-axillary or pseudot erminal, branches pubescent, few flowered; bracteoles 3, Sepals 5, persistent, pubescent, outer sepa ls 3, lanceolate, inner sepals 2, p etaloid, subfalcate: Petals 3, con nate in lower 2/5, blue-purple; L ateral petals obovate, keel with fi mbriate appendages: filaments u nited below 2/3 into sheaths, she aths ciliate
Fruit	Capsule orbicular, ca. 4 mm in diam, narrowly winged, eciliate	Capsule subcordate, ca. 5 mm in diam., narrowly winged, shortly ciliate, apex retuse.
Seed	Seeds black, ovoid, ca. 2 mm in dia m., densely white pubescent, with 2-lobed decurrent strophiole.	Seeds black, compressed-oblong, ca. 1.5 mm, densely white pubes cent, with white strophiole.
Fl. and f r.	Fl. and fr. May-Sep.	Fl. Apr-Jul, fr. May-Aug.

Supplementary Table 2 Counterfeit products of PR and their characteristics

Source plant	Characteristics	
	Botanical characteristics	Pharmacological characteristics
Dodartia orientalis Linn. (Yehuma)	The surface is brownish yellow with a slight bitter taste and no prick-throat feeling when chewing.	It is sweet in flavor and neutral in nature. It has the effect of clearing heat and removing toxicity, dispelling wind and arresting itching.
*Lysimachia insignis Hesml. (*Sanyexiangcao*)*	Its root is cylindrical, often curved, 5-20cm long, 2-5mm in diameter, its surface is smooth, light reddish-brown with numerous small brownish-red spots and sparse fine root marks. It tastes lightly spicy.	It is pungent and bitter in flavor and warm in nature. It has the effect of expelling wind and dredging collat, promoting qi to activate blood.
Cynanchum atratum Bge (Baiwei)	Its diameter is 0.1 ~ 0.2cm. Its surfce is brownish yellow with slight longitudinal wrinkles and without transverse wrinkles. No stinging sensation in the throat when chewed.	It is bitter and salty in flavor and cold in nature, and enters the stomach meridian, liver meridian and kidney meridian. It has the effect of clearing heat and cooling blood, inducing diuresis for treating stranguria as well as detoxifying and healing sores.
Liriope muscari (Decne.) Baily. (Shanmaidong)	It is thinly cylindrical and slightly curved. Its diameter is 0.1 ~ 0.2cm. Its surface is yellowish white with fine longitudinal wrinkles. It is sticky to chew and does not irritate the throat.	It is sweet and slight bitter in flavor and slight cold in nature, and enters the heart meridian, lung meridian and stomach meridian. It has the effect of nourishing Yin and generating body fluid, nourishing the lungs and clearing the heart.
Ophiopogon japonicus (L. f.) Ker-Gawl. (Maidong)	It is slender and curved and is 3~25cm long and 1~3mm in diameter. Its surface is yellowish white and slight yellow with thin wrinkles and transverse cracks. It is flexible in texture and its section is yellowish white and slightly transparent. It is mild, sweet and sticky to chew.	It is sweet and slight bitter in flavor and slight cold in nature, and enters the heart meridian, lung meridian and stomach meridian. It has the effect of nourishing Yin and generating body fluid, nourishing the lungs and clearing the heart.

Source plant	Characteristics	
	Botanical characteristics	Pharmacological characteristics
Polygala japonica Houtt. (Guazijin)	It is 10~30cm long and 1~3mm in diameter. Its surface is tawny with vertical and horizontal li nes as well as node.	It is pungent and bitter in flavor and neutral in n ature, and enters the lung meridian. It has the ef fect of expelling phlegm to arrest coughing, pro moting blood circulation and and detumescence, relieving swelling and detoxication.

Supplementary Table 3 The ancient processing methods of PR

Category	Adjuvant	Method	Purpose	Ref.
Purification processing	None	Pounding then remove wood heart	Purification	The Collective Notes to Canon of Materia Medica (本草经集注)
	Water or wine	Filing, washing then remove wood heart, soaking in wine, peeling off the phloem and remove wood heart		Effective Prescriptions for Universal Relief (普济本事方)
	Water or licorice	Soaking with water, soup or boiling with licorice then remove wood hear		Prescriptions People's Welfare Pharmacy (太平惠民和剂局方), Treatise on Three Categories of Pathogenic Factors (三因极一病证方论), Zhu Shi Ji Yan Yi Fang (朱氏集验方)
	Rice-washing wate	Soaking in rice-washing water, then pounding and remove wood heart.		De Pei Ben Cao (得配本草)
Plian stir-frying		Stir-frying slightly	Reducing toxicity and mitigat ing its properties	Orthodox Manual of External Diseases (外科正宗)
		Stir-frying to yellow		Effective Prescriptions for Universal Relief (普济本事方)
		Baking		Jifeng's Formularies for Universa l Relief (鸡峰普济方)
		Stir-frying to scorch	Generating hemostasis	Classified Treatment (类证治裁)

Category	Adjuvant	Method	Purpose	Ref.
Stir-frying with adjuvants	Licorice	Soaking in cooked licorice soup overnight after removing wood heart.	Relieving dryness and throat irrit ation, Enhancing lung moistenin g	Master Lei's Discourse on Drug Processing (雷公炮炙论)
		Washing by licorice soup		Zeng Guang Yan Fang Xin Bia n (增广验方新编)
		Decocting with licorice juice		Xiao Er Wei Sheng Zong Wei L un Fang (小儿卫生总微论方)
		Soaking in cooked licorice soup overnight then baking to dry		Prescriptions People's Welfare Pharmacy (太平惠民和剂局方)
		Soaking in licorice juice then steaming.		Xian Xing Zhai Guang Bi Ji (先醒斋广笔记)
Stir-frying with adjuvants	Wine	Steaming with wine	Enhancing kidney tonifying, hea rt calming and removing toxicity for detumescence	Prescriptions People's Welfare P harmacy (太平惠民和剂局方)
		Stir-frying with wine		Effective Prescriptions for Univ ersal Relief (普济本事方)
		Soaking in wine		Jifeng's Formularies for Univers al Relief (鸡峰普济方)
	Ginger	Stir-baking with ginger	Relieving gastrointestinal irritati on and enhancing expectorant.	Effective Prescriptions for Univ ersal Relief (普济本事方)
	Wheat	Stir-frying with wheat	Enhancing sedation	Pu Ji Fang (普济方)
Complex proce ssing	Licorice, gi nger	Decocting with licorice then stir-frying with ginger	Reducing toxicity and enhancing moistening lung	Effective Prescriptions for Univ ersal Relief (普济本事方)
	Black bean s, licorice	Decocting with juice of black beans and licorice	Reducing toxicity and drug resist ance, enhancing tranquilization a nd tonic	Jing Yue's Complete Work (景岳全书)

Category	Adjuvant	Method	Purpose	Ref.
	Pig bile, ginger	Decocting with pig bile and dry, then stir-frying with ginger	Relieving gastrointestinal irritation and enhancing expectorant.	Curative Measures for All Diseases (万病回春)
	Rice-washing water, licorice	Soaking in rice-washing water and licorice	Enhancing nourishing spleen and replenshing qi	De Pei Ben Cao (得配本草)
	Black beans, licorice, ginger	Decocting with juice of black beans and licorice then stir-frying with ginger	Reducing toxicity and drug resistance, enhancing tranquilization and tonic	Yi Xue Ru Men (医学入门)

Supplementary Table 4 The mordern processing methods of PR

Category	Operation	Adjuvant amount	Purpose	Ref.
stir-fryed PR	Stir-frying with slow fire until the surface is brown and yellow with slightly charred spots.	No mentioned	Reducing toxicity, mitigating bias	Processing Specification of Chinese and Tibetan Medicinal Materials in Gansu Province (Announcement in 2020-2022)
deep-fryed PR	Mixing PR with water of licorice decocted twice, absorbing water with slow fire, taking out, drying and sieving, stir-frying with strong fire until the surface is burnt black and the inside is burnt brown, spraying a little water and dry in the sun.	Every 100kg of PR, 7kg of licorice are is used.	Reducing toxicity, mitigating bias	Processing Specification of Chinese Medicinal Materials in Beijing (2023 Edition)
cinnabar mixed PR	Taking licorice stir-fryed PR, spraying appropriate amount of water, slightly suffocating for about 10 minutes, sprinkling cinnabar fine powder, mixing well and dry.	15.625g fine cinnabar powder is used for every 500g of PR.	Enhancing the nerves-calming effect	Processing Specification of Frequently-used Chinese Medicines in Gansu Province (1972 Edition)
	Taking PR, spraying appropriate amount of water, covering tightly and moistening for 1-2 hours, sprinkling with extremely fine powder of cinnabar, mixing well, spreading out and drying.	No mentioned	Enhancing the nerves-calming effect	Processing Specification of Chinese and Tibetan Medicinal Materials in Gansu Province (Announcement in 2020-2022)
	Taking licorice stir-fryed Yuanzhi Rou, spraying water and sealeding slightly, make it slightly stuffy, mixing and air-drying	1400g cinnabar powder is used for every 50kg of PR.	Enhancing the nerves-calming effect	Cutting Specification of Chinese Medicinal Materials in Beijing (First Volume)
Honey stir-fryed PR	Mixing refined honey diluted with boiling water with PR, until the honey is completely absorbed, and stir-frying with slow fire until the color is deepened and nonsticky hand.	25kg refined honey is used for every 100kg of PR.	Moistening lung, relieving cough	Processing Specification of Chinese Medicinal Materials in Hebei Province (First Batch) (2023 Edition)

Category	Operation	Adjuvant amount	Purpose	Ref.
	Frying PR according to honey-stir-frying method (China Pharmacopoeia, 2020 edition, four general rules 0213) until it doesn't stick to hands.	20kg refined honey is used for every 100kg of PR.		Processing Specification of Chinese Medicinal Materials in Shanxi Province (Announcement in 2022)
	Frying PR according to honey-stir-frying method (China Pharmacopoeia, 2020 edition, four general rules 0213) until the color is deepened and nonsticky hand.	No mentioned		Processing Specification of Chinese Medicinal Materials in Chongqing (First Batch) (2022 Edition)
	Mixing refined honey diluted with boiling water with PR, sealeding thoroughly, and stir fry with slow fire to a specified degree.	25kg refined honey is used for every 100kg of PR.		Processing Specification of Chinese Medicinal Materials in Ningxia
Stewed PR	Putting licorice and PR together in a medicine pot, add water, and stir-fry with bran fire.	Every 100 g of PR is added with 5 times of water and 7 g of licorice.	Eliminating irritation to throat, enhancing intelligence and calming nerves	(Song et al., 2023)

Supplementary Table 5 Oligosaccharide esters isolated from PR

NO.	Compounds	Parent nucleur	R								Molecular formula	Ref.
			R_1	R_2	R_3	R_4	R_5	R_6	R_7	R_8		
71	Tenuifoliose A	A	-	a	e	b	a	-	i	a	$C_{62}H_{76}O_{35}$	(Li, 2008; Li et al., 2011)
72	Tenuifoliose B	A	-	H	e	b	a	-	i	a	$C_{60}H_{74}O_{34}$	(Li, 2008; Li et al., 2011)
73	Tenuifoliose C	A	-	H	e	b	a	-	i	H	$C_{58}H_{72}O_{33}$	(Li, 2008; Li et al., 2011)
74	Tenuifoliose D	A	-	a	e	b	a	-	i	H	$C_{60}H_{74}O_{34}$	(Zhao et al., 2020)
75	Tenuifoliose E	A	-	a	e	b	a	-	i	H	$C_{58}H_{72}O_{33}$	(Zhao et al., 2020)
76	Tenuifoliose F	A	-	a	e	b	a	-	m	a	$C_{68}H_{86}O_{39}$	(Zhao et al., 2020)
77	Tenuifoliose G	A	-	a	e	b	a	-	m	H	$C_{66}H_{84}O_{38}$	(Li, 2008; Miyase et al., 1992)
78	Tenuifoliose H	A	-	a	e	b	a	-	e	a	$C_{61}H_{74}O_{34}$	(Miyase et al., 1992)
79	Tenuifoliose I	A	-	a	e	b	a	-	e	H	$C_{59}H_{72}O_{33}$	(Li, 2008; Miyase et al., 1992)
80	Tenuifoliose J	A	-	H	e	b	a	-	e	a	$C_{59}H_{72}O_{33}$	(Miyase et al., 1992)
81	Tenuifoliose K	A	-	H	e	b	a	-	e	H	$C_{57}H_{70}O_{32}$	(Li, 2008; Miyase et al., 1992)
82	Tenuifoliose L	A	-	a	e	b	a	-	n	a	$C_{67}H_{84}O_{38}$	(Miyase et al., 1992)

NO.	Compounds	Parent nucleur	R								Molecular formula	Ref.
			R_1	R_2	R_3	R_4	R_5	R_6	R_7	R_8		
83	Tenuifoliose M	A	-	a	e	b	a	-	n	H	$C_{65}H_{82}O_{37}$	(Miyase et al., 1992)
84	Tenuifoliose N	A	-	a	i	b	a	-	i	a	$C_{63}H_{78}O_{36}$	(Miyase et al., 1992)
85	Tenuifoliose O	A	-	H	i	b	a	-	i	a	$C_{61}H_{76}O_{35}$	(Miyase et al., 1992)
86	Tenuifoliose P	A	-	H	i	b	a	-	i	H	$C_{59}H_{74}O_{34}$	(Miyase et al., 1992)
87	Tenuifoliose Q	A	-	H	e	b	a	-	n	a	$C_{65}H_{82}O_{37}$	(Jiang and Tu, 2003)
88	3,6'-Disinapoyl sucrose	B	H	H	H	j	j	H	H	H	$C_{34}H_{42}O_{19}$	(Miyase et al., 1999)
89	Sibiricose A1	B	H	H	H	H	j	H	H	H	$C_{23}H_{32}O_{15}$	(Miyase et al., 1999)
90	Sibiricose A2	B	H	H	H	H	l	H	H	H	$C_{24}H_{34}O_{15}$	(Jiang et al., 2011; Miyase et al., 1999)
91	Sibiricose A3	B	H	H	H	g	H	H	H	H	$C_{19}H_{26}O_{13}$	(Miyase et al., 1999)
92	Sibiricose A4	B	H	H	H	j	H	H	H	j	$C_{34}H_{42}O_{19}$	(Miyase et al., 1999)
93	Sibiricose A5	B	H	H	H	i	H	H	H	H	$C_{22}H_{30}O_{14}$	(Miyase et al., 1999)
94	Sibiricose A6	B	H	H	H	j	H	H	H	H	$C_{23}H_{32}O_{15}$	(Jiang and Tu, 2003; Miyase et al., 199 9)
95	Tenuifoliside A	B	H	H	H	l	g	H	H	H	$C_{31}H_{38}O_{17}$	(Ikeya et al., 2008)
96	Tenuifoliside B	B	H	H	H	j	g	H	H	H	$C_{30}H_{36}O_{17}$	(Ikeya et al., 2008)
97	Tenuifoliside C	B	H	H	H	j	j	H	H	H	$C_{35}H_{44}O_{19}$	(Jiang et al., 2011; Zhou et al., 2014)

NO.	Compounds	Parent nucleur	R								Molecular formula	Ref.
			**R_1**	**R_2**	**R_3**	**R_4**	**R_5**	**R_6**	**R_7**	**R_8**		
98	Tenuifoliside E	B	H	a	H	h	a	H	H	a	$C_{41}H_{42}O_{22}$	(Ikeya et al., 1994)
99	Tenuifoliside F	C	S6	H	S7	-	-	-	-	-	$C_{30}H_{36}O_{17}$	(Yang et al., 2022)
100	Tenuifoliside G	C	H	S7	S8	-	-	-	-	-	$C_{34}H_{42}O_{18}$	(Yang et al., 2022)
101	Tenuifoliside D	D	l	-	-	-	-	-	-	-	$C_{18}H_{24}O_9$	(Ikeya et al., 2008)
102	Sibiricose A7	D	x	-	-	-	-	-	-	-	$C_{17}H_{22}O_9$	(Wang et al., 2005; Zhou et al., 2014)
103	Polygalatenoside B	E	b	-	-	-	-	-	-	-	$C_{20}H_{18}O_{11}$	(Cheng et al., 2006)
104	Tenuifoliose W	F	S3	S2	S4	S1	S1	H	-	-	$C_{60}H_{74}O_3$	(Yang et al., 2022)
105	Tenuifoliose X	F	S3	S2	S5	S1	S1	H	-	-	$C_{66}H_{84}O_{38}$	(Yang et al., 2022)
106	Tenuifoliose Y	F	S4	S2	H	S3	H	H	-	-	$C_{56}H_{70}O_{32}$	(Yang et al., 2022)
107	Tenuifoliose Z	F	S4	S2	H	S5	H	S3	-	-	$C_{72}H_{88}O_{39}$	(Yang et al., 2022)
108	Polygalatenoside A					-					$C_{20}H_{18}O_{11}$	(Cheng et al., 2006; Zhou et al., 2014)
109	Polygalatenoside C					-					$C_{20}H_{18}O_{11}$	(Cheng et al., 2006; Zhou et al., 2014)
110	Polygalatenoside D					-					$C_{20}H_{29}O_{13}$	(Cheng et al., 2006)
111	Polygalatenoside E					-					$C_{22}H_{32}O_{13}$	(Cheng et al., 2006)
112	Sucrose					-					$C_{12}H_{22}O_{11}$	(Liu et al., 2010)
113	Glomeratose A					-					$C_{24}H_{34}O_{15}$	(Sun, 2005)

NO.	Compounds	Parent nucleur	R								Molecular formula	Ref.
			R1	R2	R3	R4	R5	R6	R7	R8		
114	Glomeratose B					-					$C_{32}H_{38}O_{17}$	(Sun, 2005)
115	Arillanin A					-					$C_{33}H_{40}O_{18}$	(Li, 2008)
116	Arillanin B					-					$C_{22}H_{30}O_{14}$	(Li, 2008)
117	Arillanin C					-					$C_{23}H_{32}O_{15}$	(Li, 2008)
118	β-D-(3-O-sinapoyl)-fructofuranosyl-α-D-(6-O-sinapoyl)-glucopyranoside					-					$C_{34}H_{42}O_{19}$	(Ikeya et al., 2008)
119	3’-O-3, 4, 5-trimethoxycinnamoyl-6-O-4-methoxy benzoyl sucrose					-					$C_{31}H_{38}O_{16}$	(Li, 2008; Zhou et al., 2014)
120	α-D-(6-O-sinapoy)-glucopyrabosyl (1→2)-β-D-(3-O-sinapoyl)-fructofuranos					-					$C_{34}H_{42}O_{19}$	(Li et al., 2005)
121	β-D-3-O-benzoyl-6-O-fructofuranosyl-[6-O-acetyl]-[3-O-β-D-glucopyrabosyl-(4-O-benzoyl)-α-D-glucopyranoside					-					$C_{36}H_{44}O_{19}$	(Li, 2008)
122	2-(6-O-β-D-xylopyranosyl-β-D-glucopyrabosyl)-benzoic acid methyl ester					-					$C_{19}H_{26}O_{12}$	(Li, 2008)

NO.	Compounds	Parent nucleur	R								Molecular formula	Ref.
			R1	R2	R3	R4	R5	R6	R7	R8		
123	5,3'-dihydroxy-7,4'-dimethoxyflavonol-3-O-β-D-glucopyranoside					-					$C_{23}H_{24}O_{12}$	(Zhou et al., 2014)
124	3′-O-(O-methylferuloyl) sucrose					-					$C_{20}H_{24}O_{15}$	(Vinh et al., 2020)
125	4-O-Benzoyl-3′-O-(O-methylsinapoyl)-sucrose					-					$C_{28}H_{30}O_{17}$	(Vinh et al., 2020)
126	6-O-(O-methyl-p-benzoyl)-3′-O-(O-methylsinapoyl) sucrose					-					$C_{28}H_{29}O_{18}$	(Vinh et al., 2020)
127	6,3′-di-O-sinapoylsucrose					-					$C_{29}H_{32}O_{20}$	(Vinh et al., 2020)
128	Polygalasibiricose I					-					$C_{57}H_{70}O_{30}$	(Song et al., 2012)
129	3-O-(3,4,5-trimethoxy-cinnamoyl),6′-O-(p-methoxybenzoyl) sucrose ester					-					$C_{34}H_{44}O_{15}$	(Son et al., 2022)
130	1-O-(cinnamoyl),3-O-(benzoyl),2′-O-(6-O-acetyl-β-d-glucopyranosyl) sucrose ester					-					$C_{35}H_{42}O_{18}$	(Son et al., 2022)

Note: a=acetyl; b=benzoyl; c=acetone; d=p-methylbenzoyl; e=(E)-p-coumaroyl; g=p-hydroxylbenzoyl; j=(E)-sinapoy; i=(E)-feruloyl; f=benzoyl; k=3,4-dimethoxylcinnamoyl; l=(E)-3,4,5-trimethoxycinnamoyl; m=4-O-α-L-rhamnopyranosyl-(E)-feruloyl; n=4-O-α-L- rhamnopyranosyl-(E)-p-coumaroyl; p=3,4-dimethoxylcinnamoyl; q=cinnamoyl; x=a-hydroxy-3,5-dimethoxycinnamoyl.

Supplementary Table 6 Pharmacological effects of PR

Diseases	Drug administration	Model	Study design	Positive control	Result	Mechanism	Ref.
Anti-AD	PSM-04, 0.1, 0.5, or 1 μg/mL, 12h	L-Glu, $A\beta_{1-42}$ and H_2O_2 treated Rat Primary Cortical Neuron	In vitro	200 ng/ml BDNF	Inhibited cell death and ROS production,	Reducing apoptosis and ROS production	(Park et al., 2019)
	PSM-04, 5 or 10 mg/kg, p.o., 2mth	Tg mice/WT mice	In vivo	Donepezil	Increased memory index, reduced Amyloid plaques in the cortex and gliosis, increased SOD-2 and BDNF protein expression		
Anti-AD	TEN, 50 — 400 μmol/L, 100 μmol/L was the best dosage, 4h	20 μmol/L $A\beta_{25-35}$ treated PC12 cell	In vitro	-	Increased LRP1 expression, decreased RAGE expression, inhibited cell cycle activation, increased Bcl-2 mrna expression, decreased Bax, Caspase-3, and Caspase-8 mrna expression and enzymatic activity	Accelerating the transport and clearance of Aβ to resist Aβ neurotoxicity.	(Yu et al., 2022)
Anti-AD	RP02-1, 0 — 8.62μM 24h	CHO/APP BACE1 cell	In vitro	-	Inhibited $A\beta_{42}$ production and aggregation, enhanced IDE and NEP expression to promote $A\beta_{42}$ degeneration	Attenuating $A\beta_{42}$ production and inhibiting $A\beta_{42}$ aggregation	(Zeng et al., 2020b)
Anti-AD	PTPS, 50 or 100 mg/kg, i.g., 60d	SAMP8 mice	In vivo	1 mg/kg donepezil hydrochloride	Improved cognitive disorder, reduced cellular damage in the CA3 region, reduced AChE content, increased ACh expression, decreased Aβ content in the hippocampus, increased IDE, inhibited apoptosis, enhanced density of dendritic branches and spines, upregulated BDNF and TrkB protein expression, p-ERK/ERK ratio, CREB and p-CREB proteins content, mediated ERK protein expression	Activating ERK pathway and inhibiting Aβ damage	(Li et al., 2024)
Anti-AD	DISS, 0, 5 or 50 μM, 10d	$A\beta_{1-42}$ transgenic C. Elegans	In vitro	-	Increased life span and fertility, decreased paralysis, reduced ROS production, lipofuscin accumulation and Aβ deposition, increased mrna expression of daf-16, sod-3, gst-4, lgg-1 and bec-1 gene, decreased that of decreased that of daf-2 and daf-15.	Regulating expression of genes related to antioxidation and autophagy to reduce Aβ deposition and neurotoxicity	(Tang et al., 2022)
Anti-AD	TEN, 8 mg/kg, i.p., 8w	APP/PS1 double-transgenic mice	In vivo	-	Improving learning ability, increased PSD-95 expression, decreased p-tau Ser231、Ser214、Ser396 protein levels	Reducing Aβ deposition and tau overphosphorylation	(Wang et al., 2020c)
Anti-AD	2% TEN, 5d	Tau-transgenic Drosophila	In vivo	-	Increased climbing index, entering the barrier-free centrifugal tube number, half-death time, average lifespan and maximum lifespan	Not mentioned	(Lu et al., 2021)
Anti-AD	2% TEN, 5d	Tau-transgenic Drosophila	In vivo	-	Increased half-death time, average lifespan and maximum lifespan, the protein and mrna epressions of PI3K and AKT	Modulating PI3 K/AKT signaling pathway	(Ren et al., 2022)

Diseases	Drug administration	Model	Study design	Positive control	Result	Mechanism	Ref.
Anti-AD	0.5%,1%,2%, 4%,8% TEN, 7d	Tau-transgenic Drosophila	In vivo	-	Increased climbing index, entering the barrier-free centrifugal tube number, dtor mrna and protein expression	Modulating dtor signaling pathway	(Song et al., 2022)
Anti-SCI	Water extract, 10 μg/ml or its constituents 0.01, 0.1, or 1 μM, 24h	10 ng/ml IFN-γ or TNF-α treated Microglia	In vitro	-	Increased M2 number and M2: M1 ratio	Resisting inflammation and preventing axonal degeneration	(Kuboyama et al., 2021)
Anti-neurological diseases	RP01-1, 0, 100, 500 or 1000 μg/mL 72h	PC12 cells, primary cortical neuron	In vitro	25 ng/ml NGF	Promoted the neuritogenesis, induced phosphorylation of AKT, ERK, CREB and BDNF expression	Modulating AKT, ERK, CREB signaling pathways	(Zeng et al., 2020a)
Anti-AD	OE, 50, 100, 200 and 400 mg/L, 24h	$A\beta_{25-35}$ induced SH-SY5 Y cell	In vitro	-	Increased survival rate, decreased apoptosis, increased BDNF, pakt/Akt, pcreb/CREB expressions	Activating Akt/CREB phosphorylation and promoting the expression of BDNF	(Niu et al., 2022)
Anti-AD	80, 200, 300 and 800 μg/ml PR and 0 — 1,000 μM tenuifolin	Hippocampal cells from pregnant Wistar rats	In vitro	-	Increased filamentous drebrin, dcreased rebrin cluster densities (EC_{50} of tenuifolin = 481 μM, 95% confidential interval: 175 — 3,600 μM, EC_{50} of PR = 26.1 μg/ml, 95% CI: 2.75–111 μg/ml), and the effect of PR depend on NMDAR partially	Improving synaptic plasticity	(Koganezawa et al., 2021)
Anti-AD	TEN, 20, 40, 80 mg/kg/d, i.g., 3mth	APP/PS1 double-transgenic mice	I vivo	-	Increased LC3 IOD values, LC3, Cathepsin D, Rab7, PINK1, Parkin mrna expressions in in brain mitochondria, and LC3, PINK1 and Parkin protein expressions, decreased p62 mrna and protein expression, its effect was blocked by autophagy inhibitor	Activating PINK1/Parkin signaling pathway and improving lysosome function	(Lu et al., 2021)
Anti-AD	Tenuifolin, 40 μmol/L, 24h	20 μM $A\beta_{25-35}$ induced PC 12 cell	In vitro	-	Downregulated MMP, reduced PINK1, Parkin and LC3II/I protein expressions, upregulated P62 protein expression	Modulating PINK1-Parkin mediated granular autophagy pathway	(Li et al., 2023c)
Anti-NDD	TEN, 0, 20, 40 and 60 μM, 24h	30 μM $A\beta_{1-42}$ treated HT22 cells	In vitro	-	Inhibited cell demage, increased cell viability and MMP, reduced apoptosis and ROS, induced mitophagosomes and mitolysosomes formation, promoted conversion of LC3 I to LC3 II, downregulated p62 expression, promoted full-length PINK1 accumulation and Parkin to mitochondria translocation, decreased mitochondrial matrix protein HSP60 expression to activating mitophagy activated by PINK1/Parkin	Modulating PINK1/Parkin signaling pathway to induce mitophagy	(Tian et al., 2022)

Diseases	Drug adminis tration	Model	Stu dy desi gn	Positive control	Result	Mechanism	Ref.
Anti-SC I	TEN, 60 mg/k g/d, i.g., 7d	SCI SD rats	In v ivo	-	Reduced autophagy, restored locomotor functions,	Blocking PTPN1 and resc uing the IRS1/Akt/mtor si gnaling to inhibit autopha gy	(Zang et al., 2023)
Anti-AD	Tenuifolin, 20, 40, or 80 mg/ kg, i.g., 90d	APP/PS1 double-tran sgenic mice	In v ivo	-	Improved cell morphology, increased T-AOC surum level, CAT, GSH-Px a nd SOD activities, MMP, Bcl-2 expression and Bcl-2/Bax ratio, reduced M DA level and Bax expression	Protecting mitochondrial f unction, resisting oxidatio n and apoptosis	(Jin et al., 20 22)
Anti-AD	Tenuifolin, 10, 20 or 40μmol/ L, 26h	10 μmol/L Aβ_{1-42} an d D-galactose treated HT-22 cell	In v itro	-	Increased cell survival rate, reudced Cyt c positive expression, increased int racellular atpase activity, enhanced MMP, reduced TNF-α, IL-6 levels, inhi bited NF-κB and COX-2 expression, increased PPARγ, PGC-1α expression	Upregulating PPARγ/PG C-1α signaling pathway, i mproving mitochondrial f unction and blocking infla mmation	(Li et a l., 202 3b)
Anti-PD	Onjisaponin B, 20 or 40 40 mg/kg/d, 12d	MPTP treated C57BL /6J mice	In v ivo	120 mg/k g/d mado par	Prevented degeneration of DA neurons, improved motor impairment, inhibi ted microglia over-activation, decreased secretion of TNF-α, IL-1β and IL-6, reduced excessive lipid epoxidation, increased antioxidant enzyme activi ty, inhibited p65 subunit of NF-κB expression, reduced rhoa and ROCK2 pr oteins expression	Modulating rhoa/ROCK2 signaling pathway to resis ting oxidative stress and n flammation	(Peng et al., 2020)
Anti-AD	TEN, 1, 5 or 1 0 μM, 2h	Aβ1 — 42 itreated B V2 microglial cells	In v itro	-	Inhibited TNF-α, IL-1β and IL-6 release, inos and COX-2 expression, inhib ited activation of NF-κB and its translocation to the nucleus	Downregulating NF-κB si gnaling pathway to resisti ng oxidative stress and inf lammation	(Chen and Ji a, 202 0)
Anti-SD induced cognitiv e disorde r	Tenuifolin, 10 and 20 mg/kg, i.g., 28d	Sleep deprivation C57 BL/6J mice	In v ivo	0.1 mg/k g Huperz ine A	Improved memory impairments, increased IL-10 geneation, reduced IL-1β, IL-6 and IL-18 generation, activated microglia, increased NRF2 and HO-1 expressions, decreased NLRP3 and caspase-1 p20 expressions, upregulated BDNF signaling cascade, reduced impaired hippocampal neurogenesis	Resisting oxidative stress and inflammation, protect ing neuron	(Jiang et al., 2023)
Anti-AD	50% (v/v) etha nol extract of aerial parts, 2 5, 50, 100 mg/ kg/d, 14d	Scopolamine injected KM mice,	In v ivo	Piraceta m, 2 mg/ kg/d i.p.	Improve learning and memorying, increased ach and ChAT levels in the hip pocampus and prefrontal cortex, BDNF and IL-10 levels, SOD and GSH co ntent, reduced IL-1β and AchE levels, and MDA level	Regulating cholinergic act ivity, promoting BDNF an d inhibiting neuroinflamm ation and oxidative stress.	(Wang et al., 2020b)

Diseases	Drug adminis tration	Model	Stu dy desi gn	Positive control	Result	Mechanism	Ref.
Anti-AD	0% (v/v) ethan ol extract of a erial parts, 25, 50, 100 mg/k g/d, 46d	D-gal and nano2 120 and 90 mg/kg, i. P., K M mice	In v ivo	200 mg/k g piraceta m	Improve learning and memorying, increased ach and ChAT levels, decrease d ChAT levels, reduced IL-1β and MDA levels, increased IL-10, GSH and SOD activities, increased protein and mrna expressions of TrkB, BDNF	Modulating cholinergic ac tivity, inhibiting neuroinfl ammation and oxidative st ress, and regulating the B DNF and TrkB signaling p athway.	(Zhang et al., 2020)
Anti-ND D	Polygala sapo nins (PSS), 5, 10 or 20 mg/ L, 24h	$A_{\beta 1-42}$, A53T-α-synu clein-, or Q74-induce d BV2 cells	In v itro	-	Increased co-localization of LC3 and mitochondria and number of autophag ic vacuoles surrounding the mitochondria, upregulated SHP-2 to activate A MPK/mtor and PINK1/parkin signaling pathways, inhibited activation of N LRP3 and was blocked by autophagy inhibitor	Modulating SHP-2-media ted mitophagy to inhibit th e NLRP3 inflammasome	(Qiu et al., 20 22)
Anti-ND D	TEN, 1.5, 2, 2. 5 or 3 μmol/ L, 2h	200 ng/m L LPS treat ed BV2 microglia cell s	In v itro	10 μmol/ L Dexam ethasone	Inhibited NO concentration, reduced COX-2 mrna expression and protein l evel	Inhibiting inflammatory f actors release and express ion	(Pi et a l., 202 0)
Anti- N DD	Tenuifolin, 30 mg/kg/d, i.g., 2w	0.2% CPZ, p.o., C57B L/6J mice	In v ivo	-	Enhanced motor coordination and myelin content, inhibited microglia activ ation and inflammation, reduced IL-1β and TNF-α levels, increased Nrf2 an d HO-1 protein expressions	Inhibiting microglia activ ation, infllammation and o xidative stress	(Li et a l., 202 3d)
Anti-AD	Polygalacic ac id, 6 or 12 mg/ kg/d, i.g., 3w	410 pmol/5 μL $A\beta_{42}$ i njected C57BL-6 AD mice	In v ivo	-	Improved cognitive function, reduced TNF-α and IL-1β levels, increased P PARγ expression, decreased phosphorylated NF-κB/NF-κB and phosphoryl ated IκBα/IκBα expressions	Modulating PPARγ/NF-κ B pathwa to reduce inflam mation	(Zhao and Ji a, 202 4)
Anti-ND D	PTP70-2, 3, 6 or 12 μM, 2h	1 μg/ml LPS treated B V2 microglial cells	In v itro	5 μM mi nocycline	Inhibited TNF-α、IL-6、IL-1β、COX2 and inos mrna levels and inos and COX2 protein expressions, reduced TLR4 and myd88 expressions, inhibite d NF-κB p65 translocation to nucleus, inhibited its NF-κB signaling	Modulating TLR4-mediat ed myd88/NF-κB signalin g pathway	(Chen et al., 2022)
Anti-AD	Crude polysac charide (PTB), 600 mg/kg/d, i.v., 14d	50μg LPS injected SD rats	In v ivo	2 mg/kg/ d HA	Improved cognitive function, reduced NO, TNF-α, IL-1β, and IL-6 in seru m, inhibited microglia activation, inhibited neurotoxicity	Inhibiting neuroinflamma tion	(Zeng et al., 2022)
Anti-AD	DISS, 20, 40 a nd 60 μM, 24h	APP- NSCs	In v itro	-	Increased cell viability, inhibited LDH release, increased brdu+ cells, DISS and Tenuifolin increased diameters of APP- NSCs, DISS recovered migrati on of APP-nscs, DISS and onjisaponin B rescued the differentiation defects of APP- NSCs, DISS performed best among them	Increasing hippocampal n eurogenesis	(Wang et al., 2021)
Anti-AD	Tenuifolin, 10 μM, 2 h	GCA treatedht-22 cell s	Invi tro	-	Prevented intracellular calcium overload, abnormal calpain system and BD NF/TrkB signaling downregulation, decrease of cell viability was prevented by calpeptin and ferroptosis inhibitor instead of ferroptosis	Inhibiting ferroptosis and neuronal apoptosis, maint aing the balance of calpai n system	(Li et a l., 202 3a)

Diseases	Drug adminis tration	Model	Stu dy desi gn	Positive control	Result	Mechanism	Ref.
Anti- me nopause cognitiv e dysfun ction	70% ethanol e xtracts, 1, 10 o r 100 mg/kg, 3 w	160 mg/kg VCD, i.p., female C57BL/6 mice		100 μg/k g E2	Improved learning and memory, increased AChE, BDNF, and Bcl-2-associa ted athanogene expression	Exerting estrogen-like eff ects	(Han e t al., 2 021)
Anti-age ing relat ed cogni tive imp airment	RPS	400 μM H_2O_2 treated Neuro-2a cell	In v itro	-	Reduced C3 complement protein in aged mice, enhancd beneficial bacteria and suppressed harmful bacteria, regulated multiple molecular markers rela ted to neuroinflammation and aging, improved the behavior and extends the lifespan of C. Elegans	Anti-aging and regulating intestinal flora	(Zeng et al., 2021)
Anti-IC H	TEN, 8, 16, or 32 mg/kg, 3d	Collagenase injected C57BL/6J ICH mice	In v ivo	-	Improved neurological functions, reduced brain water content, haematoma volume and hemoglobin content, inhibited MBL-C and Car1 expression	Modulating the compleme nt system	(Wang et al., 2024)
Anti-my ocardial damage	The root and a erial part of P R, 2.4, 1.2, or 0.6 g/kg, i.g., 7d	100 mg/kg isoprotere nol s.c., wistar rats	In v ivo	-	Inhibited ST segment elevation of electrocardiogram, reduced the cardiac i ndex, ctnt and CK-MB levels in serum, increased SOD and CAT activities, reduced MDA level	Resisting oxidation	(Fu et al., 20 22)
Sedative and hyp notic eff ects	TMCA, 50, 10 0 or 200 μg/ra t, i.c.v.	repeated cold exposur e or intracerebroventr icular injection of cort icotrophin-releasing h ormone induced stress	In v ivo	-	Prolonged sleeping time, decreaseing NE content in LC	Inhibiting NE conten in L C	(Kawa shima et al., 2004)
Sedative and hyp notic eff ects	YZ-II, 20, 40, 80 mg/kg, i.p., 10d	PCPA induced ICR m ice	In v ivo	2.0 mg/k g, Diazep a i.p.	Reduced immobility time, prolonged sleep time, enhanced concentrations o f 5-HT, NE, PGD2, IL-1β and TNF-α, regulated GABAARα2, GABAARα 3, GAD65/67, 5-HT1A and 5-HT2A, as well as levels of DPR, PGD2, iNO S and TNF-α	altering serotonergic, GA BAergic and immune syst ems	(Hao e t al., 2 024)
Anti-oxi dation	70% ethanol e xtract, 0.2, 0. 4, 0.6, 0.8, 1.0 or 1.2 g/L	scavenging experime nts of DPPH· and AB TS+·	In v itro	2 mL Vit amin C	The clearance rates of DPPH· = 97.13% and ABTS+·= 81.45%	Resisting oxidation	(Guo e t al., 2 019)

Diseases	Drug administration	Model	Study design	Positive control	Result	Mechanism	Ref.
Anti-fatigue and oxidation	Polysaccharide, 0.10, 0.20 or 0.40mg/g/d, 30d	Exhaustive Mice exercise model	In vivo	0.10 mg/g·d, Panax quinquefolium L.	Prolonged weight-loaded swimming time, increased concentrations of LG and MG, LDH activity, decreased BLA and BUN content, The clearance rates of OH· = 61.3%, DPPH = 79.5%	Not mentioned	(Xie, 2021)
Anti-ageing	Water extract of aerial part, 25, 50 or 100 mg/kg, i.g. 45 d	100mg/kg/d D-Glu injected mice	In vivo	100mg/kg Vitamin E	Enhanced learning ability, reduced MDA content, increased SOD, CAT, GSH-Px and T-AOC activity	Inhibiting peroxide production	(Zhang et al., 2019)
Anti-inflammation	TCMB, 6.25, 12.5, 25 or 50 μM, 1h	100 ng/mL LPS treated RAW 264.7 cell	In vitro	-	Reduced mRNA expression levels of iNOS, COX-2, TNF-α, IL-1β, and IL-6, downregulated protein levels of iNOS and COX-2	Inhibiting inflammation	(Son et al., 2022)
Anti-lung cancer	PTP, 0, 50, 100, 200, 400, 600 or 800μg/mL, 24, 48 or 72 h	SPC-A-1 cell	In vitro	-	Upregulated expression of FAS, ligand FAS-L, FADD and LC 3B-II, down regulated P62	Induceing FAS/FAS-L-mediated apoptosis and autophagy	(Yu et al., 2020)
Anti cancer	PTP, 0, 100, 200, or 400 μg/ml, 24h	S180 cell	In vitro and in vivo	-	Induced apoptosis, decreased MMP, induced ROS production, increased PS translocation, increased ratio of BAX and Bcl-2, promoted CytC release, upregulted caspase-9/-3, regulated immune organ indexes,	Inducing apoptosis and activating immunoregulation mechanism	(Yu et al., 2021)
Anti cancer	Euxanthone Cu (II), 0 — 50 μg/mL	ECA109, SGC7901, Hela	In vitro	-	Inhibited cell growth	Inducing DNA damage in cancer cells	(Song et al., 2019)
Anti-virus	Water decoction, 0 — 50 μg/mL, 1h	SARS-CoV-2 treated Vero E6 cell	In vitro	remdesivir	IC_{50} = 9.5 μg/ml, CC_{50} = 186.3 μg/ml, SI = 19.7, reduced viral titer, inhibited virus at full-entry, entry, and post-entry treatments	Inhibiting SARS-CoV-2	(Ngwe Tun et al., 2022)
Anti-MAFLD	seed oil, not mentioned, 8w	HFD-induced KM mice	In vivo	-	Inhibited plasma and hepatic levels of TC and TG, reduced, improved hepatic lipid accumulation, reduced IL-6 and TNF-α levels	Inhibiting lipid accumulation and NF-κB signaling pathway	(Xin et al., 2023)
