# Supplementary material for: A review of the botany, metabolites, pharmacology, toxicity, industrial applications, and processing of Polygalae Radix: the “key medicine for nourishing life”
Source: Front Pharmacol. 2024 Sep 18;15:1450733. doi: 10.3389/fphar.2024.1450733 (PMC11445616; doi:10.3389/fphar.2024.1450733)
Supplement: Supplementary file 2 [file DataSheet1.docx]

**ETHNOPHARMACOLOGY**

**Ancient records**

PR has been used in China for centuries and is favored by ancient and modern doctors. “Er Ya”, the first dictionary in China, has the earliest record about the name of PR: “Yaorao, Jiyuan”, which Guo Pu (A.D. 276-324) annotated as: “It refers to the present “Yuanzhi”, which looks like ephedra, with red flowers and sharp, yellow leaves, and its leaves were called Xiaocao”. Tao Hongjing reported another name of PR: “Xicao” in Variorum of Shennong’s Classic of Materia Medica (本草经集注) (Southern and Northern Dynasties, A.D. 480-498). By the Song Dynasty, “Tu Jing Ben Cao”（图经本草）mentions that “Nowadays, doctors only use ‘Yuanzhi’ and rarely use ‘Xiaocao’ “, which indicated that “Yuanzhi” has been established as the name of PR and its root was considered to be its main medicinal part(Feng, Ge et al. 2019).

PR was first recorded in Shen Nong’s herbal classic (神农本草经), which shows that it has a medicinal history of thousands of years. In this work, PR was listed as the top grade and considered to be warm in nature, bitter in taste and non-toxic, could treat cough caused by the lung Qi upward reversal replenish the body's deficiency, open orifice, eliminate pathogenic factors, improve hearing and vision, reinforce intelligence and treat forgetfulness, strengthen will and body, Long-term eating can make the body relaxed and not aging.

Supplementary Records of Famous Physicians (名医别录) written in the late han dynasty, states that it is non-toxic, can calm the mind, stop palpitation, improve the essence, remove the diaphragm Qi under the heart, remove the burning sensation in the skin and sallowness on the face. Long-term consumption of it can improve the complexion.

“Yaoxing Lun” (药性论) (A.D. 618-907) accounts that it can cure the distraction of mind and amnesia, tonify yang and treat nightmares.

Wang Haogu, a famous Chinese doctor in Yuan Dynasty, said that it could enter the kidney meridian and could be used to treat diseases caused by kidney cold ascending.

Materia medica of Southern Yunnan (滇南本草): “It enters the heart, liver and spleen meridian. It can nourish heart and blood, relieve convulsion, dispel phlegm and saliva. It can also treat opisthotonos, convulsion, foam at mouth, hands tremble, fainting, loss of consciousness, stranguria due to chyluria, red and white turbidity as well as spermatorrhea”.

Compendium of Materia Medica (本草纲目) (A.D. 1552-1578) mentions that it could cure all kinds of carbuncles and abscess.

“Bencao Jingshu” (本草经疏) (Ming Dynasty, A.D. 1625) describes that it is bitter and slight pungent in flavour and warm in nature.

“Yuqiu Yaojie” (玉楸药解) (Qing Dynasty, A.D. 1754) accounts that it enters the heart meridian of hand Shao Yin and the kidney meridian of foot Shao Yin. It can be used to treat necrosis, breast carbuncle and all kinds of sores and swelling.

“Bencao Zaixin” (本草再新) (Qing Dynasty, A.D. 1841) reports that it has the effect of promoting qi circulation and relieving depression, and it is good at eliminating phlegm.

Records of Tradition Chinese and Western Medicine in Combination (医学衷中参西录) (A.D. 1909): Yuanzhi, whose sour taste can astringe, pungent taste can disperse, so its nature is effective at regulating the lungs. It can make the lungs open and close freely, so that the breathing in the lungs can be regulated and phlegm can also be dissolved, then the cough stops. If it used with licorice as its adjuvants, it is really an important medicine to nourish the lungs. The astringent effect of its sour taste can converge and regulate liver fire after entering the liver meridian; it can enter the kidney meridian with the effect of inducing astringency and arresting discharge; it can also help the production of gastric acid when it enters the stomach meridian, and make people have a good appetite.

Dictionary Of Traditional Chinese Pharmacy (中药大辞典）states that people with excessive yin deficiency and heart and kidney fire should avoid taking it.

In the Chinese Pharmacopoeia 2020 edition, PR was introduced to be bitter and pungent in flavour and warm in nature, and it can enter the heart, kidney as well as lung meridian. At the same time, PR has the functions of soothing the mind and enhancing intelligence, coordinating the heart and kidney, eliminating phlegm and swelling. It is usually used in the treatment of insomnia and dreaminess, forgetfulness and palpitations, mental trance, cough and phlegm, sores and swelling, and breast ache caused by heart and kidney disorders. Single PR can be processed into polygala liquid extract, and the polygala liquid extract can be mixed with a certain concentration of ethanol in a certain proportion to make Yuanzhi Ding, both of them are expectorants.

Based on the above ancient and modern records, it can be summarized that with the development of history and the accumulation of medication experience, our understanding of the pharmacological effects of PR is getting more and more comprehensive and profound, its medicinal scope has been expanded, and most of its pharmacological effects have been verified by modern science. However, little attention has been given to its improvement of hearing and vision, removal of skin burning and sallow on face, as well as the treatment of urinary system diseases. The lack of evidence in basic research limits its application, therefore, these neglected traditional applications should be paid attention to in future research.

**Application in herb pair**

Herb pair refers to the relatively fixed combination of two drugs commonly used in clinical practice of traditional Chinese medicine, which is the smallest unit of prescription. It is simpler than the composition of compound prescription, and retains its advantages in enhancing curative effect, reducing toxicity and side effects. According to Variorum of Shennong’s Classic of Materia Medica (本草经集注), PR can eliminate the toxicity of Aconiti Lateralis Radix Praeparata. After long-term clinical practice and experience summarizing, PR is often combined with Acori Tatarinowii Rhizoma, Magnoliae Officinalis Cortex, Ziziphi Spinosae Semen, Glycyrrhizae Radix Et Rhizoma, Ginseng Radix Et Rhizoma and so on in order to achieve different medicinal purposes.

A study provided evidence for the potential synergy between PR and Acori Tatarinowii Rhizoma: the pharmacologically active substances of their composition increased and a substance with benzene ring was missing(Wang, Chang et al. 2012). Furthermore, their compatibility promotes the absorption of PR metabolites(Fang, Li et al. 2010). Their effects on nervous system diseases may related to anti-inflammation, anti-oxidative stress and involve a protein kinase B (AKT)1, PTGS2, TNF, and nuclear factor-kappa B (NF-κB) inflammation pathway(Su, Chen et al. 2022). Magnoliae Officinalis Cortex and PR used together with a ratio of 1:2 can regulate energy, amino acid and fatty acid metabolisms to abate the gastrointestinal inhibitory effect of PR(Hakim 1986). PR combined with Ziziphi Spinosae Semen can modulate amino acid metabolism and neurotransmitter to play a sedative role(Luo, Sun et al. 2020). The water extracts of PR and Ginseng Radix Et Rhizoma can promote hippocampal neurogenesis and the brain-derived neurotrophic factor (BDNF) signaling pathway activation so that act like antidepressants(Jiang, Wang et al. 2021).

There are few experimental studies on herb pairs of PR and the curent research only focus on a few drug pairs. It is suggested that more attention should be paid to PR herb pairs, and the changes of material basis, pharmacological effects and pharmacokinetics before and after compatibility, as well as the effects of different compatibility ratios and preparation methods on its biological activity, which will be helpful for promoting the development and application of these herb pairs and the modernization of the theory of traditional Chinese medicine drug pairs.

Component-based medicine refers to the compatibility of effective components of TCM under the guidance of TCM theory, which is indicated by Academician Boli Zhang as “an important direction of modernization of TCM”. PR related Component-based medicine together with their efficacy and mechanism are listed in Table 5. Comparatively speaking, the current researches on pharmacological mechanism of the composition provides data support for its application, but in general, there is still a long way to go due to the lack of feedback from clinical observation as well as in vivo metabolism and safety research.

**Application in Prescription**

According to Collected Works of Materia Medica (本草汇言) (Ming Dynasty, A.D. 1624), Zeshi Shen said that PR could tonify heart when combined with Ginseng Radix Et Rhizoma, poria and Atractylodis Macrocephalae Rhizoma, nourish spleen when combined with astragali Radix, Glycyrrhizae Radix Et Rhizoma and Atractylodis Macrocephalae Rhizoma, reinforce kidney when combined with Rehmanniae Radix, Lycii Fructus and Dioscoreae Rhizoma, tonify liver when combined with Paeoniae Radix Alba, Angelicae Sinensis Radix and Chuanxiong Rhizoma, tonify lung when combined with Ginseng Radix Et Rhizoma, Ophiopogonis Radix and Adenophora Stricta. The compound preparation obtained by the compatibility of more than two Chinese herbal medicines like this has the characteristics of multi-components and multi-targets, and is part of the main ways of dialectical medication in traditional Chinese medicine. PR is contained in more than 800 traditional Chinese medicine formulae and 300 Chinese patent medicine, which involves pills, granules, tablets, capsules, pills, oral liquid, ointment, syrup, mixture, powder, wine, granules. About 30 prescriptions are included in the Chinese Pharmacopoeia (2020 edition) and three most classical prescriptions: “Kaixin san”, “Rehmannia drink” and “Guyin jian”(Weng, Zhao et al. 2020). The dosage range of PR applied in decoction is 1.104-30g, the usual dosage is 10-15g, and its dosage in pills and powders is 0.31-1.035g(Yu and Zhao 2022).

Here are some suggestions about the future study of PR related prescriptions: (1) There are multitudinous Chinese patent drugs and prescriptions containing PR in Chinese antique books, but their therapeutic effects need the support of scientific research. The secondary development of them by modern technical means and analysis methods such as network pharmacology, molecular docking and bioinformatics analysis will facilitate to clarify its active components and mechanism of action, provide a basis for its effectiveness, guarantee its safety, explore new targets and curative effects, broaden its application scope and promote the the advent of innovative traditional Chinese medicine products. (2) Complex pathogenic factors lead to differences in symptomatology among different patients, the study of the pharmacological effects and mechanisms of the same prescription on different symptomatic models of the same disease which is in favor of optimizing the proportion of each herb in compound prescription and providing a reference for which is holistic diagnosis and treatment in clinic. (3) The most common dosage form of PR when applied in compound prescriptions is soup, and reforming its dosage form is conducive to modernized production to achieve the effect of saving herbs and facilitating processing and taking.

**REFERENCES**

Fang, M., Li, Y., Zhang, W., Wang, Y., Zheng X. and Wang S. (2010). Effect of Acorus Tatarinowii on Pharmacokinetics of Polygala tenuifolia. J Northwest Univ (Natural Science Edition). 40(01): 85-88.

Feng, J., Ge, J. Zhao, R. Xv, L. Xie, M. and Kang, T. (2019). Textual research of radix Polygala. Chin J Ethnomed Ethnopharmacy. 28(11): 35-39.

Hakim, A. (1986). Cerebral acidosis in focal ischemia: II. Nimodipine and verapamil normalize cerebral pH following middle cerebral artery occlusion in the rat. Journal of cerebral blood flow and metabolism : official journal of the International Society of Cerebral Blood Flow and Metabolism 6(6): 676-683.

Jiang, N., Wang, H., Li, C., Zeng, G., Lv, J., Wang, Q., Chen, Y., Liu, X. (2021). The antidepressant-like effects of the water extract of Panax ginseng and Polygala tenuifolia are mediated via the BDNF-TrkB signaling pathway and neurogenesis in the hippocampus. J ethnopharmacol. 267: 113625.

Luo, H., Sun, S., Wang, Y., Wang, Y. (2020). Revealing the sedative-hypnotic effect of the extracts of herb pair Semen Ziziphi spinosae and Radix Polygalae and related mechanisms through experiments and metabolomics approach. BMC complement med. 20(1): 206.

Su, S., Chen, Y., Yang, H., Liu, H., Han, L., Wang, H., Li, W., Wang, Q. (2022). Exploration on mechanism of Polygalae Radix and Acori Tatarinowii Rhizoma in treating Alzheimer's disease based on network pharmacology and experimental verification. China J Chin Mater Med. 47(12): 3348-3360.

Wang, Y., Chang, L., Zhao, X., Meng, X. and Liu, Y. (2012). Gas chromatography-mass spectrometry analysis on compounds in volatile oils extracted from yuan zhi (radix polygalae) and shi chang pu (acorus tatarinowii) by supercritical CO2. J Tradit Chin Med. 32(3): 459-464.

Weng, Q., Zhao, J., Zhang, Y., Jin, Y., Zhang, W., Peng, H., Cai, Q., Li, B., Chen, Z., Yang, H., Zhang, H. and Zhan, Z. (2020). Textual research on classical prescription of radix Polygala. Mod Chin Med. 22(08): 1238-1244.

Yu, T. and Zhao, L. (2022). Clinical application and dose-effect relationship of milkwort root. J Changchun Univ Chin Med. 38(08): 847-850.
